# TINA manual landmarking tool: software for the precise digitization of 3D landmarks

**DOI:** 10.1186/1742-9994-9-6

**Published:** 2012-04-05

**Authors:** Anja C Schunke, Paul A Bromiley, Diethard Tautz, Neil A Thacker

**Affiliations:** 1 Max Planck Institute for Evolutionary Biology, Department for Evolutionary Genetics, August-Thienemann-Str. 2, 24306 Plön, Germany; 2 Imaging Science and Biomedical Engineering, School of Cancer and Imaging Sciences, University of Manchester, Stopford Building, Oxford Road, Manchester M13 9PT, UK

## Abstract

**Background:**

Interest in the placing of landmarks and subsequent morphometric analyses of shape for 3D data has increased with the increasing accessibility of computed tomography (CT) scanners. However, current computer programs for this task suffer from various practical drawbacks. We present here a free software tool that overcomes many of these problems.

**Results:**

The TINA Manual Landmarking Tool was developed for the digitization of 3D data sets. It enables the generation of a modifiable 3D volume rendering display plus matching orthogonal 2D cross-sections from DICOM files. The object can be rotated and axes defined and fixed. Predefined lists of landmarks can be loaded and the landmarks identified within any of the representations. Output files are stored in various established formats, depending on the preferred evaluation software.

**Conclusions:**

The software tool presented here provides several options facilitating the placing of landmarks on 3D objects, including volume rendering from DICOM files, definition and fixation of meaningful axes, easy import, placement, control, and export of landmarks, and handling of large datasets. The TINA Manual Landmark Tool runs under Linux and can be obtained for free from http://www.tina-vision.net/tarballs/.

## Background

There is an increasing level of interest in morphological and morphometric analyses, both for combination with molecular or ecological data and for a more thorough understanding of forms, e.g. investigations of shape spaces or functional morphology as well as phylogeny reconstruction (e.g. [1-7]). This is supported by modern data acquisition methodologies, mainly high resolution CT scans, which provide a multitude of characters on outer and inner surfaces. However, the approaches to landmark assignments have to be adjusted to the special situation of 3D data. 2D images of specimens allow for intersections of a structure, e.g. a suture, and the background of the image, while 3D objects have more degrees of freedom in rotation, so the same points would need a description such as e.g. being the anterior most point of a suture. A good software tool should therefore have additional options for navigating in the 3D space, but this is not fully provided by any of the currently available software packages (see software evaluation in Additional file 1). Here we describe such software which enables the user to load a stack of DICOM files, the standard output of medical imaging techniques, to calculate a volume rendering and to use a number of convenient tools for finding the optimal position for each landmark.

## Results

### Surface rendering versus volume rendering

**Figure 1:**
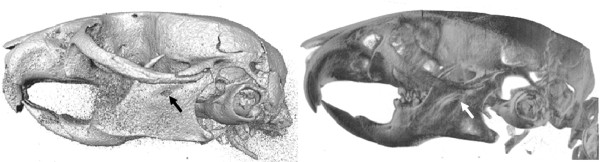
**Differences of surface rendering and volume rendering**. Two different representations of the same skull rendered with two methods. Surface rendering (left) from Amira 5.2., volume rendering (right) from the TINA Manual Landmarking Tool. Note the pseudofenestration e.g. at the end of the incisor in the mandible in the surface rendering (arrows).

Most current programs for digitization of 3D landmarks (e.g. [8]) rely on surface rendering rather than volume rendering. Surface rendering requires the definition of a threshold, i.e. a specification of the intensity of the surface of interest, and then renders a smooth surface passing through all voxels with the defined intensity. This process is based on the simplified model in which a single intensity can be used to specify all points on a specific surface. When this simplified model fails, for example because of varying properties of the surface (e.g. bone density), errors will be introduced into the rendering. These take typically the form of high noise when the threshold is too low, or non-physical holes through the rendered surface (pseudo-fenestrations) where the threshold is locally too high (Figure 1, left).

In contrast, volume rendering operates by assigning each voxel an opacity based on its intensity, and a reflectivity based on an intensity gradient, and then modelling the passage of multiple light rays through the volume. In this way, every voxel is taken into account, mitigating problems with pseudo-fenestrations and noise, and providing the user with a more informative representation of the data (Figure 1, right). Although the problems of surface rendering are well understood, it is often preferred, because it requires less computing power. Volume rendering has traditionally been too slow to allow real-time user interaction; however, advances in accelerated 3D graphics hardware and in rendering techniques, such as the shear-warp algorithm [9] implemented in the Volpack library [10] used by the TINA Manual Landmark tool, have largely removed this limitation.

### The working of the software

**Figure 2:**
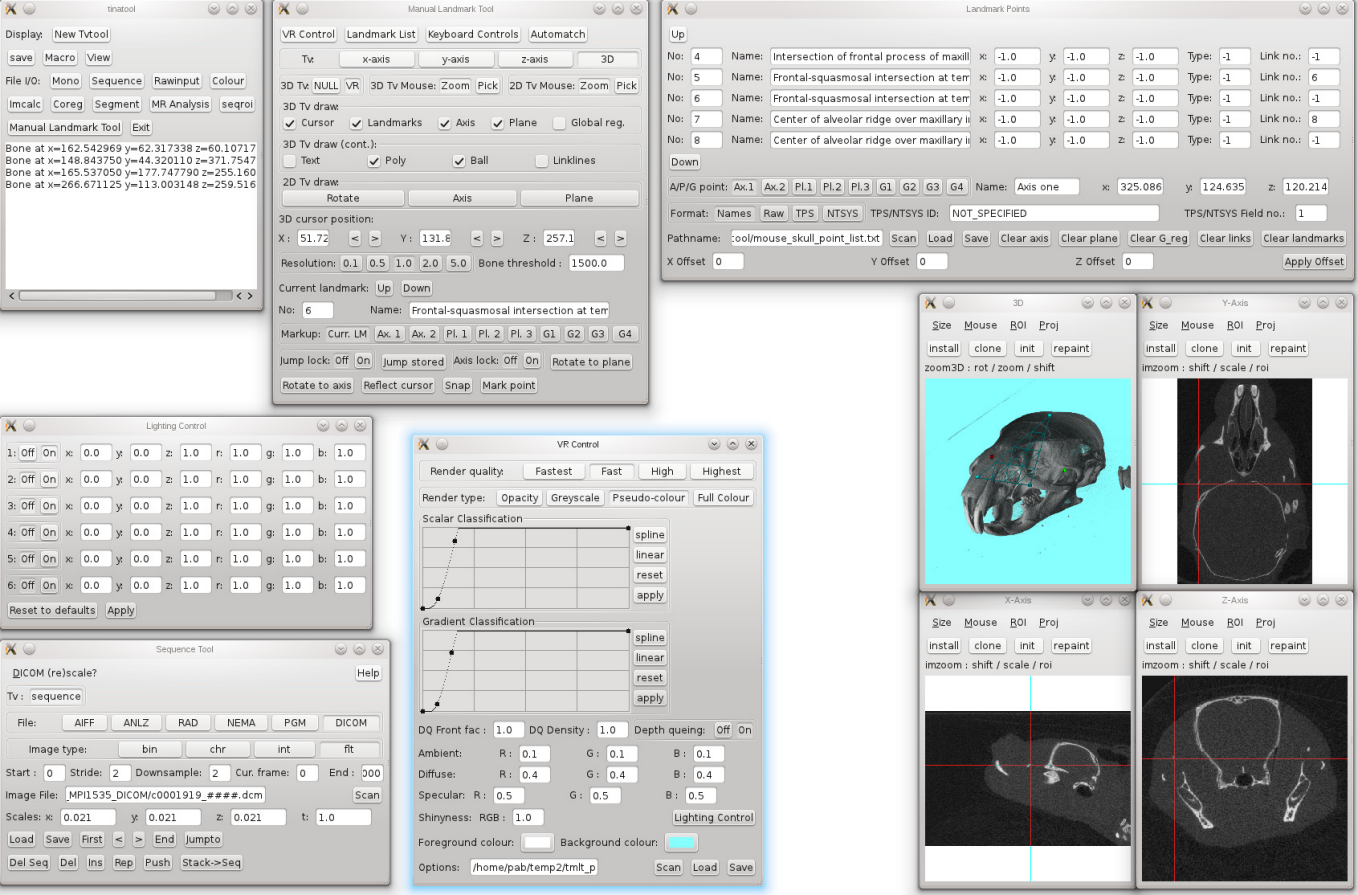
**User interface of the TINA Manual Landmarking Tool**. Screenshot of the TINA Manual Landmarking Tool during digitizing session. See text for more details.

The TINA Manual Landmarking Tool (described in detail in Additional files 2 and 3) imports stacks of DICOM files or any subset thereof. The loaded data can be down-sampled by averaging across neighbouring voxels. For high-resolution datasets, down-sampling increases both the speed of the volume rendering and, through noise reduction, the quality of the rendered images. The scanned specimen can be displayed on four different windows, so-called Tv's, one giving a 3D visualization and the other three orthogonal 2D cross-sections (Figure 2, Additional file 4). The 3D Tv has several options for the volume rendering itself as well as for additional settings. For example, it is possible to render any specific surface, or to produce a translucent rendering of all surfaces simultaneously, to change the direction, intensity, and colour of the lighting, set the background colour, and use several other criteria for the display of specimens.

**Figure 3:**
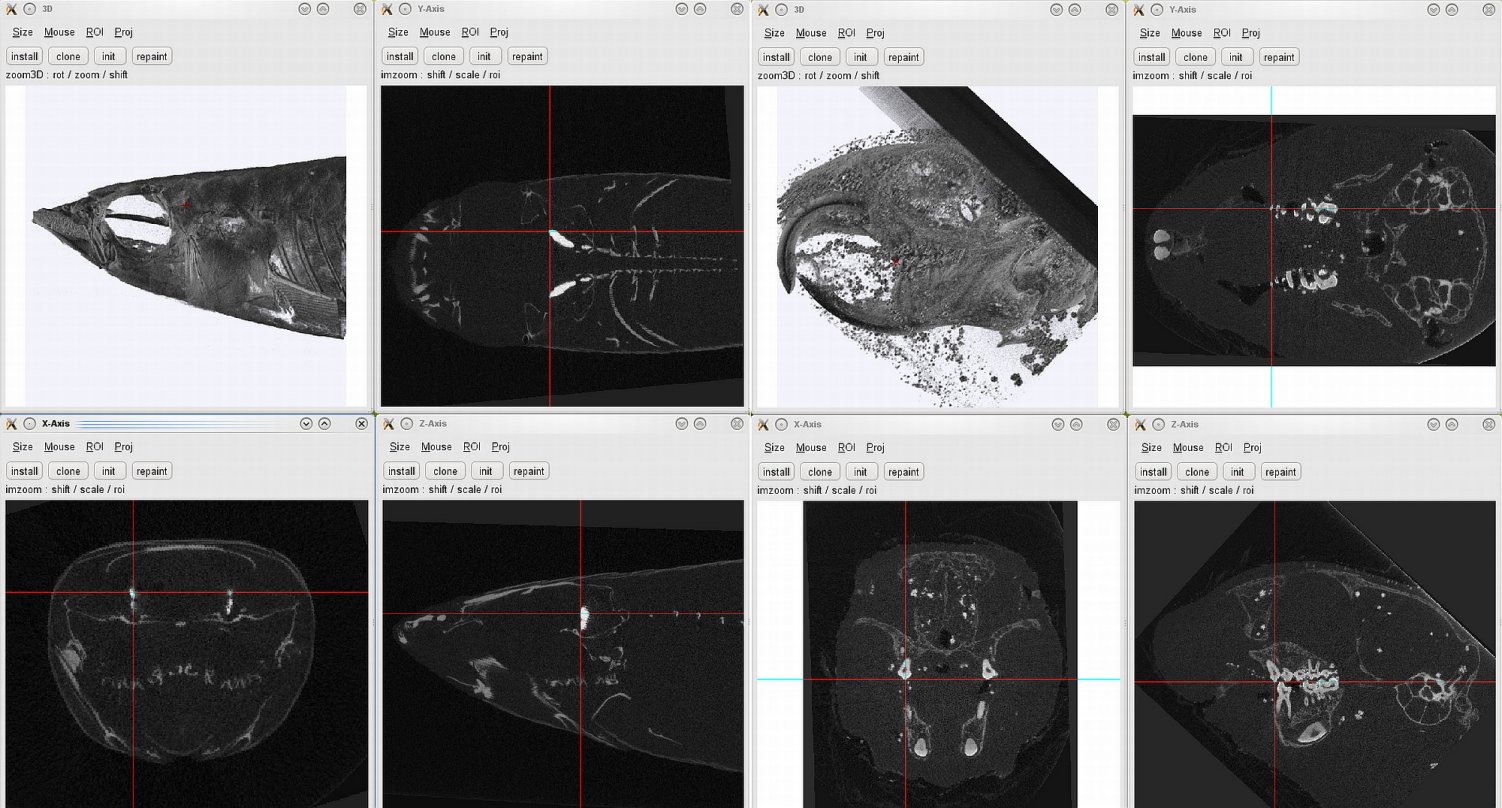
**Examples of multilayered objects**. Screenshots of the 3D and 2D Tv tools. Left: Skull of a small fish, anterior tip of otolith selected. Right: Decalcified skull with precipitations in surrounding tissue of a European Ground Squirrel, first tip of molar teeth selected.

The simultaneous display of the three 2D Tv's has an additional advantage in the use for multilayered structures, e.g. fish skulls, where the inner structures are covered by the outer ones, or noisy data, e.g. from specimens fixed in unbuffered formalin (Figure 3).

If previous landmarks are available, they can be imported from a text file with running numbers and descriptions, both being visible during the digitization process (Additional file 5). Landmarks and their numbers can also be displayed in the 3D Tv. For bilaterally symmetric structures it is possible to load another file containing information about points on the sagittal plane and paired points on both sides, thus enabling the drawing of linking lines between corresponding landmarks. The latter two options also allow a quick, visual check that the points have been marked in correct order.

All four Tv's have a synchronized cursor, indicating the same position in all of them, which enables the user to very accurately pick extreme points, like the anterior most or dorsal most position of any structure, by moving the cursor into the desired direction in one window and then marking the first pixels of the structure to appear or disappear in another Tv. The software also supports the definition of an arbitrary plane and axis to increase the precision with which extremal points are identified (Additional file 6). A plane is defined by three landmarks, e.g. in the sagittal plane of a bilaterally symmetric structure, an axis by two end points that may or may not also be used as plane points. When both plane and axis are defined, the object can be rotated accordingly, thus allowing views from precisely defined angles in all spatial directions, as well as a more reproducible digitization of extreme points relative to the defined geometry.

Landmarks identified using the 3D Tv of the TINA Manual Landmark Tool will automatically be placed on top of the first structure whose density is above a user-defined threshold. The default setting corresponds to that of mouse skull bone in a CT scan, but it can be changed to any desired value. So, if e.g. a skull is scanned in a live specimen, the landmark would pass through air and soft tissue and would be placed on the first bony structure it meets, if the threshold is set accordingly. Although this is a very convenient supplementary feature for placing such landmarks, the use of a threshold in this way could introduce errors or bias, since the location of the landmark would be dictated by pixel intensity, with no account taken of the image noise or local variations in surface intensity. Therefore, the location can then be refined using the 2D windows by moving the cursor with mouse or arrow keys.

After all landmarks are placed, the resulting coordinates can be saved and exported as TPS or NTSYS files, which allow all further statistical analyses such as principal component analyses or canonical variates analyses using e.g. the IMP series [11], MorphoJ [12], or R [13].

The majority of functions can either be conducted with the mouse buttons or with keyboard shortcuts. Default options for both are provided, but they can be customized to a large extent, e.g. for left-handed users.

## Discussion

The TINA Manual Landmarking Tool was developed in collaboration between computational scientists and a morphologist. This ensures on the one hand that computing power is optimally used and thus allows the implementation of the computational demanding, but for practical purposes superior volume rendering procedure on normal PCs. It ensures also that the tools that are offered reflect the needs of a practicing morphologist. To assess the performance of the software, we have compared the time and precision with which landmarks can be placed in positions corresponding to different types of landmarks ([14], Additional file 7), using either volume rendering plus the orthogonal cross-sections and defined axes in the TINA Manual Landmarking Tool (Additional file 8) or a typical surface rendering without defined axes in the commercially available software Amira 5.2 [8]. We found that a new user needs approximately the same time for locating landmarks in both software tools, but the precision of repeatedly identifying the same landmark is higher for landmarks placed at extremal points (type 3 landmarks according to [14]) with the TINA Manual Landmarking Tool (Additional files 9, 10).

## Availability

A Knoppix CD version for testing purposes (i.e. to be run directly from the CD - no need for installation) can be downloaded from http://www.tina-vision.net/tina-knoppix/iso/ with a demo version including a mouse skull dataset with all appropriate settings, giving an impression of how the program works. This version supports also loading of datasets e.g. from a USB stick, although necessarily with relatively long loading times. Nonetheless, this enables a potential user to check whether the intended datasets work with the tool before performing a full installation of the software. Further information on new developments on this software is continuously updated at http://www.tina-vision.net.

## Authors' contributions

ACS listed the requirements for precise and reproducible placement of landmarks, tested earlier versions of the software and suggested improvements, and drafted the manuscript. PAB developed the TINA Manual Landmarking Tool. DT and NAT initiated and supervised the project. All authors read and approved the final manuscript.

## Supplementary Material

Additional file 1 **Landmark Analysis Software Review**. This includes a comparative evaluation of available landmark analysis software, describing the various features and problems. Written by by H. Ragheb, N.A. Thacker and P.A. Bromiley. Click here for file

Additional file 2 **The TINA Manual Landmarking Tool**. This file is the manual for the Manual Landmarking Tool by P. A. Bromiley. It contains detailed information on the download, installation and use of the software. Updates are available at http://www.tina-vision.net/docs/memos.php, file 2010-007. Click here for file

Additional file 3 **Tina 5.0 User's Guide**. The file gives additional information on the TINA software in general, including features not used for the landmarking procedure. Click here for file

Additional file 4 **Movie of loading and volume rendering procedure**. This video shows the process of starting the TINA Manual Landmark tool and loading data. The user first starts the Manual Landmark tool and the Volume Rendering dialog box, opens a number of Tv's (graphics display windows), and starts the Sequence tool, which is used for loading 3D medical images. Four of the Tv's are associated with the various 2D graphics output streams of the Manual Landmark tool and the Sequence tool. The user then loads a DICOM image volume containing a 3D micro-CT image of a *Mus musculus *skull with a size of 658 × 658 voxels × 1000 slices, setting the down-sampling factor to 2 on all axes of the volume. Once the data has been loaded, the tool automatically initialises the 3D cursor (the red cross-hair) at the centre of the volume and displays three orthogonal slices through this point, aligned with the major axes of the volume. The user then switches the volume renderer on, and displays its output on the remaining Tv. The parameters of the volume renderer are then adjusted to produce an image showing the bone surface. Finally, two of the volume renderer options files distributed with the software are loaded. The first gives a pseudo-colour output, in which a grey scale rendering is given a coloured background (the foreground colour can also be adjusted in this mode to tint the grey scale rendering). The second gives a full colour rendering with multiple coloured, directional light sources. Click here for file

Additional file 5 **Movie of landmarking procedure**. This video follows on from the previous one and shows the process of identifying a landmark point. The user first loads a simple text file containing a list of landmark names and numbers; in this case the list contains only one point. The 3D rendering is then rotated so that the left-hand coronoid process is visible, and the mouse interaction is switched from "zoom" mode, which allows the rendered image to be rotated, into "pick" mode, which allows landmark points to be identified. The user places the mouse cursor approximately above the tip of the coronoid process, and left-clicks on that point. The software calculates the vector passing through the data beneath the mouse cursor, and identifies the first point along this vector with an intensity higher than the threshold specified in the Manual Landmark tool. The 3D cursor (shown by the red cross-hair) is moved to this point and the three 2D Tvs are automatically updated to show projections through the new 3D cursor position, aligned with the major axes of the volume. The user then selects the 2D Tv's and, using keyboard interaction, moves the position of the 3D cursor so that it lies exactly on the tip of the coronoid process, before clicking on the "mark point" button to store the coordinates of the 3D cursor in the list of landmark points. The 3D Tv display options are adjusted so that identified landmark points are displayed, and the 3D cursor is moved to an arbitrary point on the top of the skull so that the landmark point (the green sphere) can be seen clearly. Finally, the landmark point coordinates are output to a file in TPS format. Click here for file

Additional file 6 **Movie of geometry functions**. This video follows on from the previous one and provides a simple example of the use of the geometry functions implemented in the software. The user follows the same landmark point identification procedure demonstrated in the previous video. However, rather than identifying the points in the landmark list, the selection in the "markup" choice list is adjusted so that the coordinates of the identified points are stored in the three "plane points". The user identifies three points on the plane of bilateral symmetry, and then adjusts the 3D Tv view properties to display the plane points (the three blue spheres) and a grid representing the plane defined by these three points (the blue grid). The user then demonstrates one possible use of the geometry functions. The 3D cursor is moved to the position previously identified as a landmark point on the tip of the left-hand coronoid process, by selecting "current landmark" in the markup choice list and pressing the "jump stored" button. The 3D cursor is then projected through the plane specified by the three plane points using the "reflect cursor" function, so that it lies on the opposite side of the volume. In this way, the 3D cursor is placed close to the tip of the right-hand coronoid process; this functionality can be used to accelerate the landmark identification process when the landmark point list contains points arranged symmetrically about the plane of bilateral symmetry. The geometry functions also support the definition of an arbitrary axis defined by two "axis points". The "rotate to plane" and "rotate to axis" buttons will adjust the viewing direction of the 3D Tv so that it lies along the plane normal and the axis lies along the x-direction of the Tv. These functions facilitate the identification of landmark points that are defined as extremal points on specific structures from a specific viewing direction. Click here for file

Additional file 7 **List of landmarks used for test of accuracy**. Landmarks were assigned to the three landmark types 1, 2, and 3 (see text for details). The first three landmarks were used to define the median plane (PL1-3), landmarks 4 and 5 to define the horizontal axis (A1-2). Click here for file

Additional file 8 **Landmarks used for accuracy test**. Skulls, landmarks, planes, and axes used in the test for accuracy of repeated measurements. Left: *Microtus *(lateral view), middle: *Mus *(dorsal view), right: *Pachyuromys *(lateral view). Click here for file

Additional file 9 **Time needed for taking twelve landmarks in ten repetitions**. This plot shows the increase of speed in repeatedly taking a set of twelve landmarks on three different skulls for an experienced (left) and an inexperienced user (right). Grey: landmarks taken based on surface rendering (SR), black: landmarks taken using volume rendering (VR). Y-axis: time in minutes. Click here for file

Additional file 10 **Precision of landmarks with different methods**. The distance in voxels from the median of the respective landmark position was taken and grouped for each landmark type (see text for details). Left: experienced user, right: inexperienced user; SR: surface rendering, VR: volume rendering. Click here for file
