# Supplementary material for: TINA manual landmarking tool: software for the precise digitization of 3D landmarks
Source: Front Zool. 2012 Apr 5;9:6. doi: 10.1186/1742-9994-9-6 (PMC3353871; doi:10.1186/1742-9994-9-6)
Supplement: Additional file 1 — Landmark Analysis Software Review. This includes a comparative evaluation of available landmark analysis software, describing the various features and problems. Written by by H. Ragheb, N.A. Thacker and P.A. Bromiley. [file 1742-9994-9-6-S1.PDF]

Tina Memo No. 2009-006
Internal Report: Survey conducted for Max Planck Institute of Evolutionary Biology, Plön.

Landmark Analysis Software Review

H. Ragheb, N.A. Thacker and P.A. Bromiley

Last updated
06 / 07 / 2009

Imaging Science and Biomedical Engineering Division,
Medical School, University of Manchester,
Stopford Building, Oxford Road,
Manchester, M13 9PT.

Landmark Analysis Software Review

H. Ragheb, N.A. Thacker and P.A. Bromiley
Imaging Science and Biomedical Engineering Division
Medical School, University of Manchester
Manchester, M13 9PT, UK
neil.thacker@manchester.ac.uk

Abstract

We have studied a number of software tools dealing mainly with morphometric analysis of images reccommended by our collaborators[1]. The study not only helps us to be aware of the existing tools in the field, but also points the way towards developing our own software. The aim is to cover the project's requirements based on use in scientific analyses.

1 Introduction

There are a variety of image processing software packages which are used in the fields of medical and biomedical imaging. However, the problem with most of them is that they are not based on quantitative analysis. In some cases, they have graphical tools which are included mainly to make their list of features longer and sell well rather than tackling problems of general utility. In this paper we define criteria of interest in order to compare a number of software packages which are recommended in the field. Detailed information regarding usage are available from the references provided [5, 2, 4, 7, 1, 9] and from the software manuals. Our main goal here is to investigate whether or not any of these software could be used in the context of an automated 3D landmark detection system. The criteria used to assess this software are as follows.

- 3D visualisation tools
- 3D mark up tools
- Statistical landmark analysis tools
- Windows/Linux based
- Commercial (cost - transparency)

Among these criteria, the top three identify necessary stages in landmark identification and analysis, while the choice of operating system or the commercial origin of software are of less direct interest. We specify them for a number of practical reasons, for example Windows based software is difficult to use as part of an otherwise (Linux based) open source distribution. As full integration between Windows and Linus software is impossible, the only way to combine such software is if their input and output formats are compatible. The problem with the commercial software is that they are not only often expensive (particularly when a variety of tools are bundled together), but also not computationally transparent (hindering scientific uses). Linux based software can be provided to Windows users via KNOPPIX, which our group has some expertise with.

2 Software Studied

The software under our study are as follows.

- Landmark
- etdips
- Amira

[1]Chris Kingenberg and the Max Planck Institute of Evolutionary Biology (Plön).

- Analyze
- edgewarp
- EVAN

Below we provide some sample screen-shots together with a short description of each software package. The aim is to generate a specification for our own analysis software chain, which maximises utility within the context of existing packages.

2.1 Landmark

The goal of Landmark is to put landmark points on surfaces and to match landmark points on different surfaces to each other [9, 10]. It facilitates to quick and easy placement of landmarks on many surfaces using an atlas. Its typical inputs are 3D polygonal surfaces (.ply/.stl) with landmark points as outputs. The main idea is to use the 3D morphing tool of the software in order to study how different 3D objects (mainly skulls) vary either through time or among different regions. See figures 1 -4.

2.2 etdips

Etdips is an exploratory two and three dimensional image processing system [7, 8]. The software was co-developed by the National Institutes of Health (NIH) and the National University of Singapore (NUS) under CRADA. This is a multi-dimensional volume visualisation and analysis software package. Specifically, it supports DICOM file format as input, and, in addition to mark up points, one may output 3D surface file format (.stl). The 3D surface is the outcome of the volume rendering tool. This 3D surface may be used as input to the Landmark software too. Moreover, its inputs and outputs are compatible to those in Analyze software. See figures 5-6. One practical problem identified with this software is that it seems incapable of dealing with data-sets with the dimensions required for the mouse skull project.

2.3 Amira

Amira is a powerful, multifaceted software platform for visualising, manipulating, and understanding life science and biomedical data coming from all types of sources and modalities [1]. A long list of its features are given in the figures. Despite its coverage of many graphical tools, Amira does not add much to what the two free software, Landmark and etdips, can do for us. This therefore raises questions relating to its cost. See figures 7-12. Use of this package for manual landmark generation has identified several limitations with the interface which make it's application time consuming and restrictive. For example, iso-surface construction is often fragmented on micro-CT data, the surface intersection process for placing of landmarks is awkward, and there is no mechanism to check or correct the ordering of landmark features.

2.4 Analyze

Analyze provides interactive display, manipulation, and measurement of multi-dimensional biomedical images (CT, MRI, SPECT, PET, ultrasound and digital microscopy). It benefits from a powerful software architecture that allows multiple volume images to be simultaneously accessed and processed by multiple programs in a multi-window interface [4]. Specifically, it is entirely built upon a toolkit of optimised functions that are organised into a software development library called AVW. The AVW library is a collection of over 600 functions that are accessible to software developers to build advanced image-based application solutions. It provides full DICOM support, direct comparison of two volume images and associate object maps, volume rendering, 3D voxel and surface registration, and measurement modules. Despite providing all these features, in practice, it has failed to meet the researchers requirements at the university of Manchester. Indeed much research at Manchester over the last decade was aimed at solving problems inherent to the approaches taken to quantitative analysis in this package. See figures 13-21.

2.5 Edgewarp

Edgewarp is a package for visualisation and digitisation of solid images [2, 3]. It implements a variety of spline based morphometric techniques, including sliding landmarks on curves and surfaces navigation of multiple images

at the same time. It provides very fast navigations of volumes and has been used as part of the software being developed by EVAN [5]. What is disappointing is that it provides no multivariate statistical procedures beyond the simplest form of landmark-driven shape averaging, the Procrustes average. See figures 22-27.

2.6 EVAN

EVAN is an ongoing project which is scheduled to end in 2009 [5, 6]. Hence, its final software tools which are based on similar methods used in Edgewarp are not finalised (or available). The projects' mission is *the integration of new morphological analysis methods from bio-mathematics, computer science and bioengineering in the context of interdisciplinary studies of anatomical variability in humans, their ancestors, and their close relatives for the purpose of applications in the academic, clinical, industrial, and, private and public research sectors.* Some new methods are integrated in a network with a rich infrastructure from medical imaging, 3D-digitisation, visualisation, software programming and modelling to interlink a new generation of scientists from diverse disciplines with industrial partners and clinics. The main research goals are: 1. generation of methods, tools and data including a) methodological integration and extension (toolboxes for Virtual Anthropology, Geometric Morphometrics and Rapid Prototyping, extended methods for size-shaping analysis), and b) data archiving, and, 2. generation of applications in biology, medicine and industry. See figures 28-30.

3 Summary

In Table 1 we show how each package (listed in the left-most column) does (Y) or does not (N) correspond to the criteria listed on the top row of the table. Apparently, Edgewarp and EVAN seem to be the ones falling well in our criteria and might be expected to be the most promising. The situation is slightly more complicated than this as will be explained below.

software	3D Graphics	3D mark up	statistical analysis	Linux-based	non-commercial
Landmark	Y	Y	Y	N	Y
etdips	Y	Y	Y	N	Y
Amira	Y	Y	Y	Y	N
Analyze	Y	N	Y	Y	N
Edgewarp	Y	Y	Y	Y	Y
EVAN	Y	Y	Y	Y	Y

Table 1: A comparison between the capabilities and features of the software under study; Y (Yes); N (No).

4 Conclusions

From our investigation we have come to several conclusions. These are listed here briefly.

- DICOM images seem to be the standard image format for this work, Etdips takes DICOM, our software should also support this.
- The use of Windows as the base system precludes direct use of the Etdips package on this project. Additional problems have been encountered with current versions regarding data size.
- Exiting software produce 3D landmark points together with a 3D representation of the volume as .stl file. This seems to be standard. One may use these .stl files as input to Landmark to apply mark-up/morphometric tools. We must consider doing the same, should surface extraction become a necessity during any stage of analysis (currently we believe there are good reasons to avoid this).
- Amira might cover many functionalities but it is not only expensive but not directly relevant to landmark analysis. Previous experience with this package has shown it to be cumbersome for manual mark-up. Standard features of the data (where bone is thin) cause display problems.
- Analyze is not as comprehensive as Amira and does not have mark up tools. Moreover in practice it failed to satisfy researchers at the University of Manchester on technical grounds (such as the treatment of partial volume data).

- If Edgewarp were actually to find a reliable full non-linear warp between two 3D volumes it would provide an alternative solution to the problem of 3D feature location. We see the purpose of the current project as to find a more direct solution for the identification of key points.
- Edgewarp is an open-source software and is used in EVAN. Ideally we need to know the format used to pass landmarks between these packages, and support it in our work. As the software project has not yet delivered we must remain aware of developments.

As there seem reasons in each case why we cannot make use of the existing packages directly we will instead aim for data compatibility. We can also learn from the existing packages with regard to graphical utility while developing our own tools for manual and automatic mark-up of landmarks. In this respect, we believe that a package with similar capabilities to Etdips but for Linux will be required. A display utilising volume rendering rather than surface rendering might be more reliable for fine structures. We can also attempt to avoid the user interface restrictions found in Amira. We have started to investigate the possibilities.

With regard to analysis, we expect that 3D landmark locations generated by the automatic detection methods will be output to existing statistical analysis packages (such as MORPHOJ). However, we note that the Procrustes method, and Bookstein's equivalent techniques, currently fail to account for anisotropic measurement errors. The standard justification for this is that these errors are small in comparison to biological variability and can therefore be neglected [11]. While this argument is true for classification, it is not true for the process of parameter estimation, as can be witnessed with the difficulties encountered with poorly localised landmarks. Here, the standard method in morphometry for taking account of poorly constrained features seems to be based on the use of semi-landmarks, which move around curves (and quantify position in only one direction). For this project we intend to provide automatically measured landmarks with associated measurement co-variances. This provides an appropriate mathematical framework for handling arbitrary points defined as landmarks by users. Although we would regard this approach to be conventional use of Likelihood, this may require the development and evaluation of some statistical analysis methods new to the area of morphometry (Appendix). We expect to evaluate this approach during this project.

References

[1] Amira, http://www.amiravis.com/index.html

[2] Edgewarp, http://brainmap.stat.washington.edu/edgewarp/

[3] F Bookstein, P Gunz, H Ingeborg, P Mitteroecker, H Prossinger, K Schaefer, C Unteregger, B Wimmer, and H Seidler, Using singular warps to study morphological integration, AMERICAN JOURNAL OF PHYSICAL ANTHROPOLOGY (Suppl. 34): 46-47, 2002.

[4] Analyze, http://www.analyzedirect.com/default.asp

[5] EVAN, http://www.evan.at/

[6] S. Katina, Shape analysis in the light of simplicial depth estimators, In S. Barber, P.D. Baxter and K.V.Mardia (eds), Systems Biology and Statistical Bioinformatics, pp. 51-54, 2007, Leeds, Leeds University Press.

[7] etdips, http://www.cc.nih.gov/cip/software/etdips/index.html

[8] R. Mullick, S. V. Warusavithana, V. Shalini, and P. Pang, Plug-Ins: A Software Model for Biomedical Imaging and Visualization Research, Biomedical Imaging Symposium: Visualizing the Future of Biology and Medicine, National Institutes of Health (NIH), June 1999.

[9] Landmark, http://www.idav.ucdavis.edu/research/EvoMorph

[10] David F. Wiley, Nina Amenta, Dan A. Alcantara, Deboshmita Ghosh, Yong J Kil, Eric Delson, Will Harcourt-Smith, F. James Rohlf, Katherine St. John, Bernd Hamann, Evolutionary Morphing, in: Proceedings of IEEE Visualization, 2005.

[11] Ian L. Dryden and Kanti V. Mardia, Statistical Shape Analysis, John Wiley and Sons, 1998.

Appendix: Procrustes and Likelihood Shape Descriptions.

The Procrustes method for shape analysis involves finding the scale s_k, rotation R_k and translation t_k of a complex shape vector w_k using a least squares approach which minimises;

$$Q = \sum_k (m_0 - m_k)^T (m_0 - m_k)$$

with

$$m_0 = \frac{1}{K} \sum_k^K m_k$$

and

$$m_k = s_k R_k w_k + t_k$$

individual shapes can then be approximated as a linear model of eigen-vector e_j.

$$m_k \approx s_k m_0 + \sum_j^J \lambda_{jk} e_j$$

where λ_{jk} are linear constants, and the linear shape descriptors are normally esitmated for the space tangent to m_0 using PCA, or something equivalent.

The scale factor can be eliminated if the analysis task is required to be sensitive to specific processes (eg: biological growth). In this case the most significant eigen-vectors might be expected to describe the scaling process.

The simplest justification for this approach is to assume an isotropic distribution for the variation in m_k. However, we might well have some features which are poorly localised in one dimension. Also, by using m_0 as the target shape for alignment we cannot expect to obtain a good transformation. The resultant errors in rotation lock correlations into the resultant residuals for each m_k. Consequently, if the data had been generated by a linear model we would not expect to regenerate the parameters of that model from this analysis.

Taking acount of the data making use of the measurement distribution and constructing a self consistent model description is more complicated and only tractable if we can assume a common covariance structure C for all m_k. This is likely to be a good approximation, as the localisation error for each landmark is expected to be a function of local structure, which might be expected to be consistent across a group of shape samples.

Then, the eigen-vector analysis can be carried out in a 'whitened' tangent sub-space of the rotated data

$$u_k = W(m_k - s_k m_0)$$

in order to satisfy PCA's requirements of IID.

A Likelihood based approach can be written as follows;

$$log(P) = \sum_k^K (x_k - m_k)^T C^{-1} (x_k - m_k)$$

ie: the target for alignment is the model generated shape x_k rather than the mean.

$$x_k = m_0 + W^{-1} \sum_j^J \lambda_{jk} e_j$$

where W is a whitening matrix which follows from the measurement covariance C. The effects of the common data covariance cancel when taking the weighted mean so as before

$$m_0 = \sum_k^K m_k C^{-1} [\sum_k C^{-1}]^{-1} = \frac{1}{K} \sum_k^K m_k$$

Alternatively, by setting the first eigen-vector to the mean we will get an equivalent mathematical description of the data (ie: the subspace defined by the set of eigen-vectors will be equivalent for a good model).

These equations are only true at the solution, so optimisation of the Likelihood needs to be iterative, and designed to exploit the convergence properties of expectation maximisation ie: optimisation of the Likelihood with a fixed

distribution assumption, and re-estimation this distribution with fixed parameters. This method would proceed by initialising the scale, orientation and location of each k, and estimating the eigenvectors which establish the current estimate of data density. The process could then be iterated following re-estimation of the transformations.

As this analysis can be interpreted as performing a Procrustes analysis in a whitened data space, all previous theoretical results pertaining to the use of this method which are limited to an assumption of homogenous covariance should now apply. Notice also that we are basing the new Likelihood formulation on the measurement distribution (which is known), and not upon the distribution induced in the sample due to shape variability (which is arbitrary and the subject of our analysis).

In order to monitor the progress for the entire analysis, we can compute an estimate of the measurement covariance $\hat{C}$.

$$\hat{C} = \frac{1}{K} \sum_k (x_k - m_k)(x_k - m_k)^T$$

For a good model (eg: a sufficient number of eigen-vectors J) $\hat{C} \approx C$. For a poor model $\hat{C} >> C$. We can make this observation more quantitative by assessing the capability of the model to account for the original data distribution using probability overlaps, specifically analysis of the distribution of residuals using Bhattacharrya fitting. In this way we could expect to determine the optimal linear model order as part of the process of parameter estimation.

Clearly, as the generated model will be driven by measurement considerations and choice of landmarks, the (possibly degenerate) eigen-vectors extracted during this analysis will have no use beyond describing the subspace of a specific data sample. Quantitative scientific use of the results, which require a conclusion without reference to shape, are limited to making statements regarding equivalence of distributions (ie: object recognition). This too is one sided, as absence of measured difference is not evidence of absence of genetic difference.

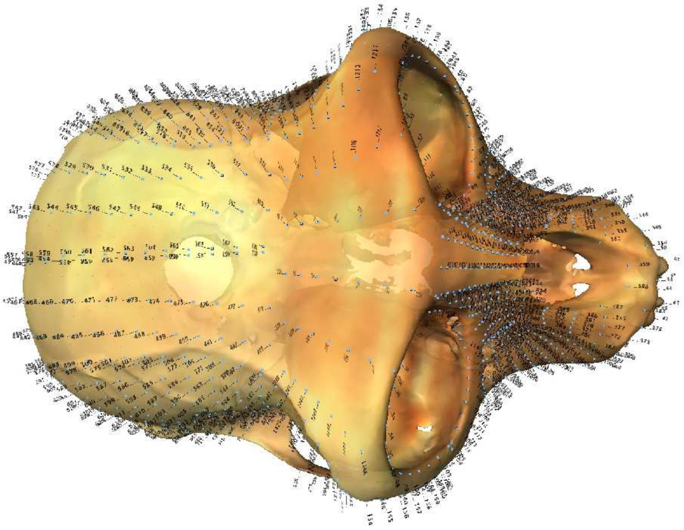

Figure 1: Landmark: mark up tool

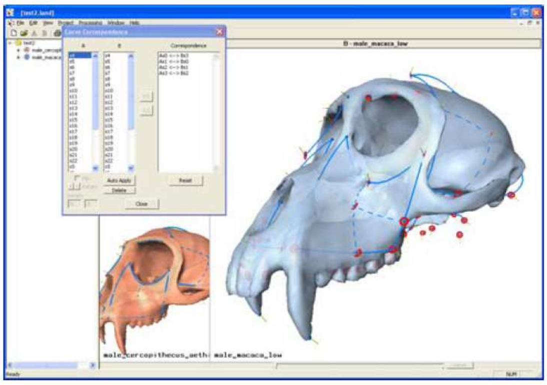

Figure 2: Landmark: semi-automatic mark up tool

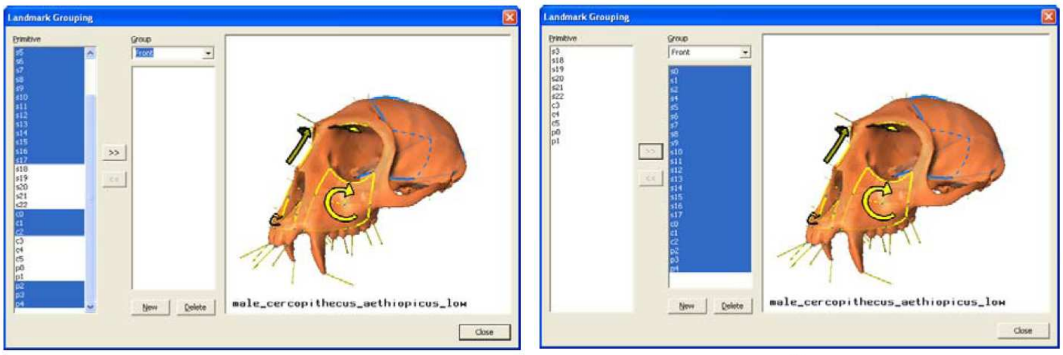

I II

Figure 3: Landmark: grouping landmarks tool

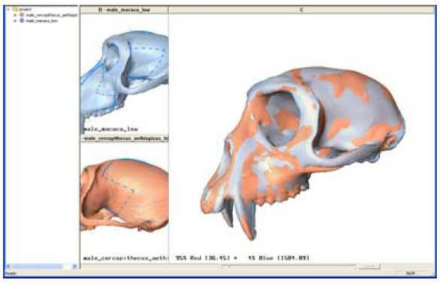

Figure 4: Landmark: morphing tool

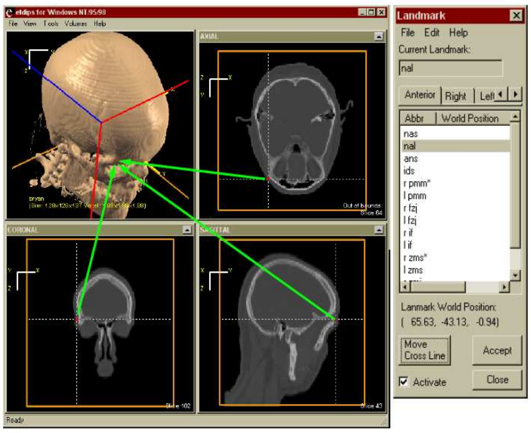

Figure 5: etdips: mark up tool

SURFACE RENDERING MODE

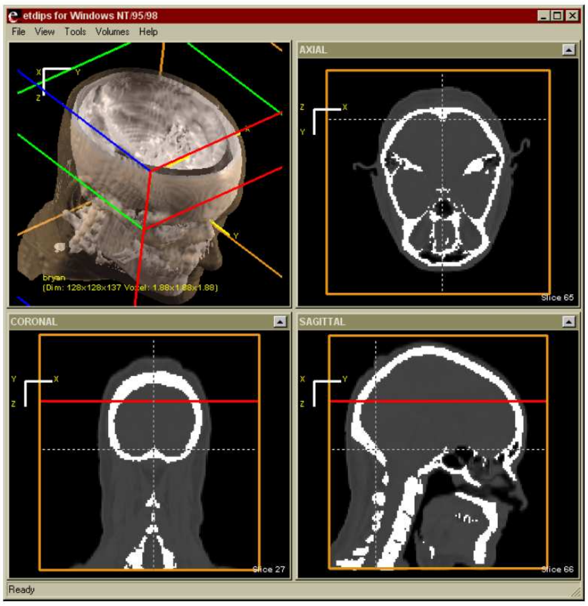

Figure 6: etdips: volume rendering tool

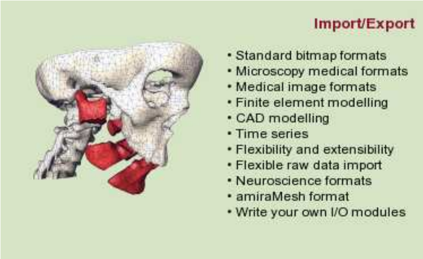

Figure 7: Amira: import/export

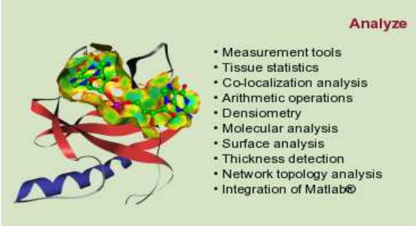

Figure 8: Amira: analyze

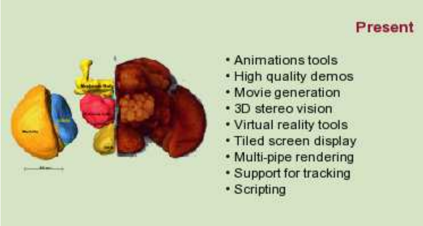

Figure 9: Amira: present

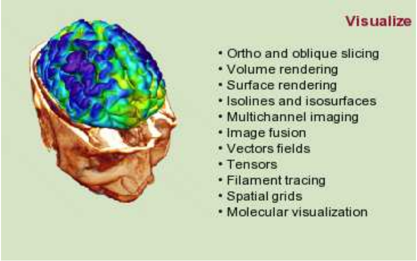

Figure 10: Amira: visualize

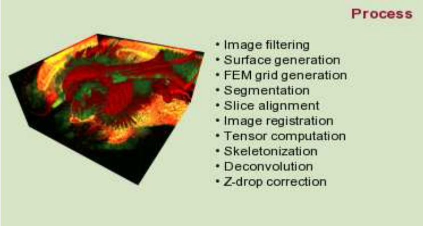

Figure 11: Amira: process

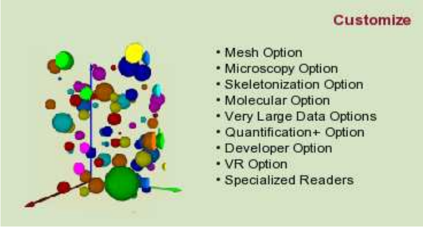

Figure 12: Amira: customize

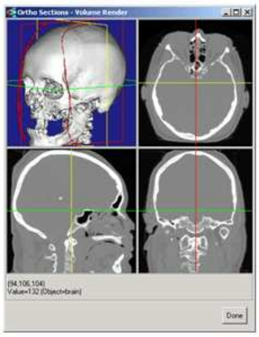

Figure 13: Analyze: volume rendering

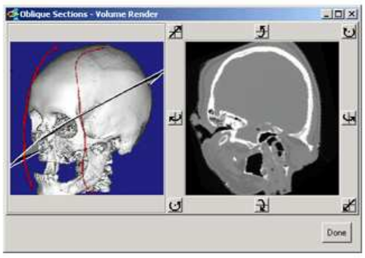

Figure 14: Analyze: rendering oblique sections

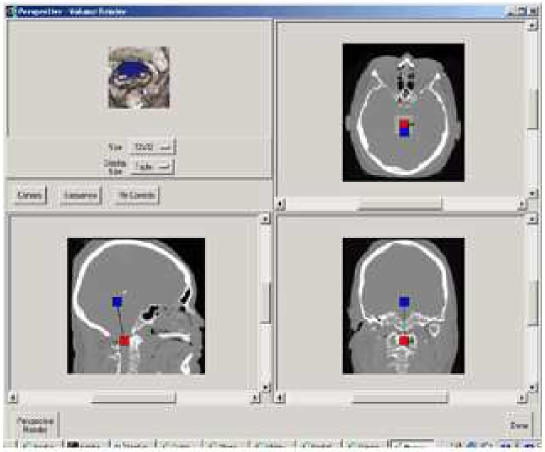

Figure 15: Analyze: measurements

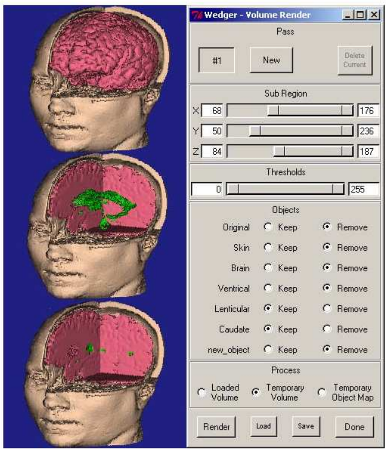

Figure 16: Analyze: rendering cross sections

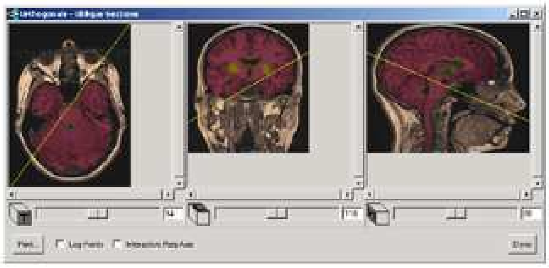

Figure 17: Analyze: oblique sections in three orthogonal views

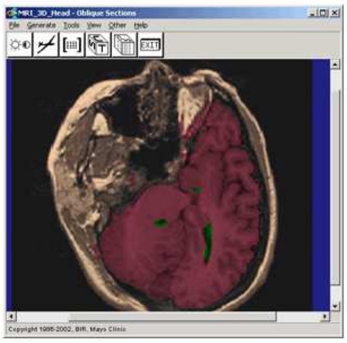

Figure 18: Analyze: oblique sections in oblique views

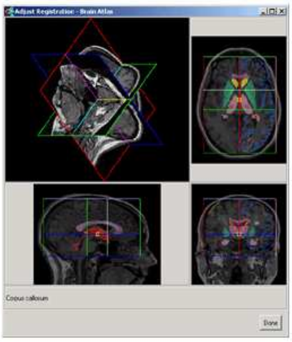

Figure 19: Analyze: brain atlas

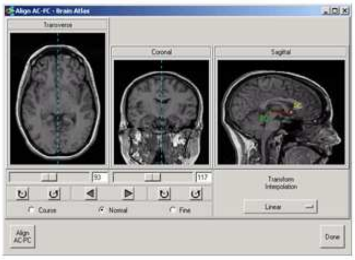

Figure 20: Analyze: brain atlas

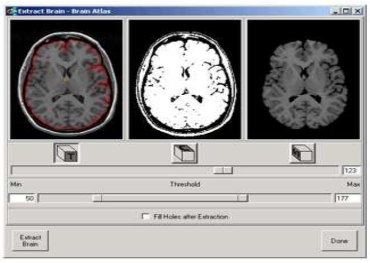

Figure 21: Analyze: brain atlas

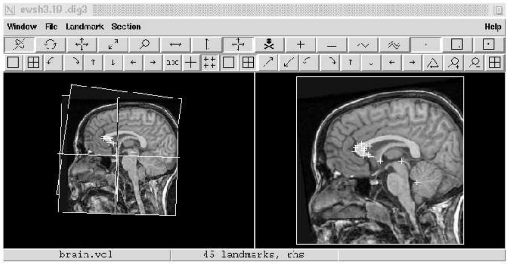

Figure 22: Edgewarp: mark up tool

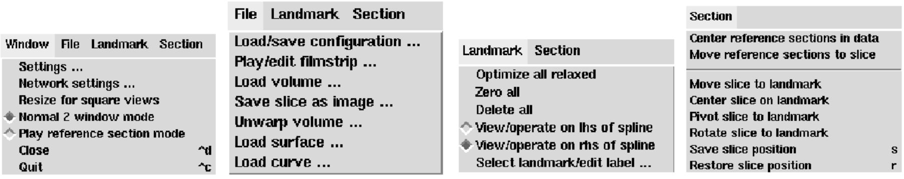

The `Windows` menu   The `File` menu   The `Landmark` menu   The `Section` menu

Figure 23: Edgewarp: Window, File, Landmark and Section menus

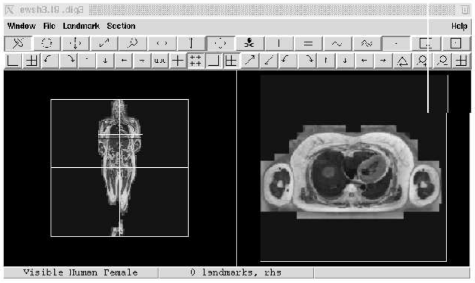

Figure 24: Edgewarp: cross sections

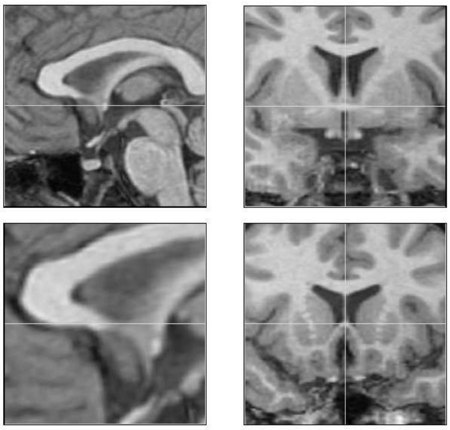

Figure 25: Edgewarp: landmark matching

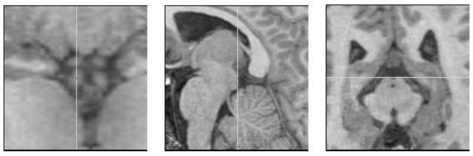

Figure 26: Edgewarp: landmarks in 3 orthogonal views

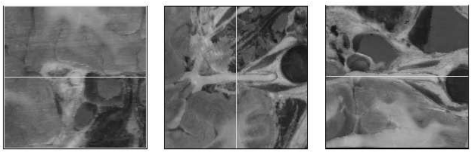

Figure 27: Edgewarp: landmarks in 3 orthogonal views

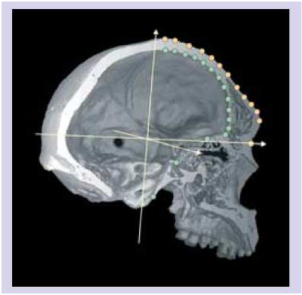

Figure 28: EVAN: mark up tool

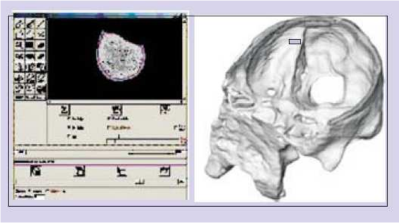

Figure 29: EVAN: volume rendering tool

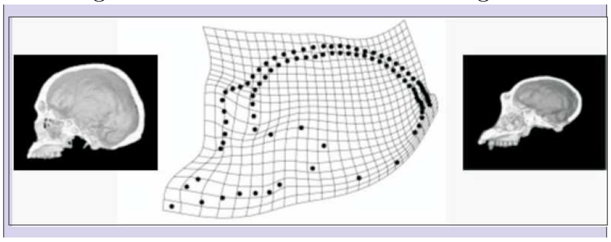

Figure 30: EVAN: morphing tool
