# Supplementary material for: TINA manual landmarking tool: software for the precise digitization of 3D landmarks
Source: Front Zool. 2012 Apr 5;9:6. doi: 10.1186/1742-9994-9-6 (PMC3353871; doi:10.1186/1742-9994-9-6)
Supplement: Additional file 2 — The TINA Manual Landmarking Tool. This file is the manual for the Manual Landmarking Tool by P. A. Bromiley. It contains detailed information on the download, installation and use of the software. Updates are available at http://www.tina-vision.net/docs/memos.php, file 2010-007. [file 1742-9994-9-6-S2.PDF]

Tina Memo No. 2010-007
Technical Memo

The TINA Manual Landmarking Tool

P.A. Bromiley

Last updated
3 / 2 / 2011

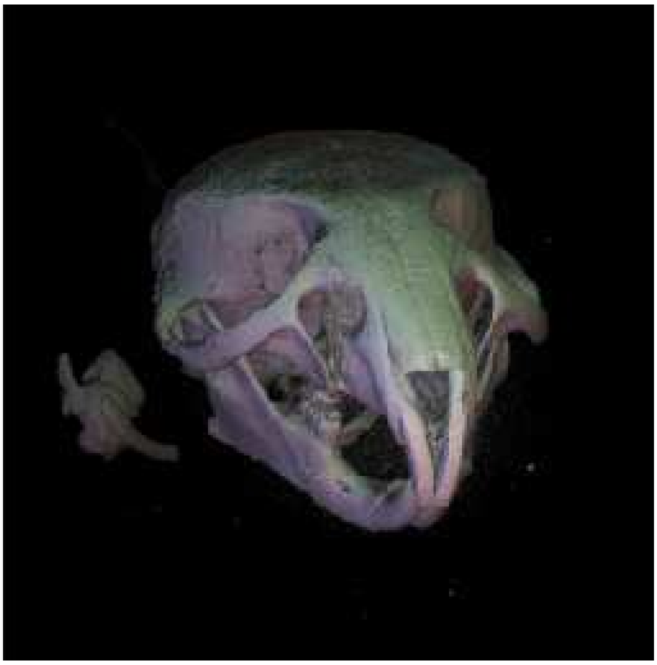

WWW.TINA-VISION.NET

Imaging Science and Biomedical Engineering Division,
Medical School, University of Manchester,
Stopford Building, Oxford Road,
Manchester, M13 9PT.

The TINA Manual Landmarking Tool

P.A. Bromiley
Imaging Science and Biomedical Engineering Division
Medical School, University of Manchester
Manchester, M13 9PT, UK
paul.bromiley@manchester.ac.uk

1 Introduction

The TINA manual landmarking tool is designed to support the manual identification and specification of morphological landmarks for subsequent analysis using techniques such as Procrustes analysis. This user manual covers:

- Section 2: installation of TINA, the Volpack volume rendering library, and the TINA Manual Landmarking toolkit on Linux systems.
- Section 3: the TINA graphical subsystem, Tv's and Tv tools.
- Section 4: the TINA Sequence tool, used for loading 3D medical data sets.
- Section 5: the Manual Landmarking tool, definition of coordinate systems, and the Tv's available in the tool.
- Section 6: the TINA interface to the Volpack volume rendering library, used to produce the volume rendered images displayed in the 3D Tv of the Manual Landmarking tool.
- Section 7: the Landmark Control dialog box, used to load, save and manipulate lists of landmark points.
- Section 8: the use of the Manual Landmarking tool to mark up the positions of landmarks specified in the landmark point list.
- Section 9: the Keyboard Controls dialog box, used to assign keyboard shortcuts to the Tv's of the Manual Landmarking tool.
- Section 10: the TINA View tool, which provides functions to save the images displayed in TINA Tv tools.
- Section 11: and example landmarking session.
- Section 12: quick reference guides, explaining the functions of each button, data entry field etc. in the Sequence tool, Manual Landmarking tool, and Manual Landmarking tool dialog boxes.

This manual covers the Manual Landmark Tool v. 1.4, which requires TINA 6.0 build number 005 or higher. Some familiarity with the Linux OS is assumed e.g. how to start and use a shell tool.

Throughout this guide, boxed sections contain detailed instructions on how to perform specific tasks with the Manual Landmarking software.

2 Installation

Installation of the TINA manual landmarking tool is done in three stages: installation of the TINA libraries, which provide the TINA machine vision functionality; installation of the Volpack library, which is used by the TINA manual landmarking tool to produce 3D volume rendered images of tomographic image data; and installation of the TINA manual landmarking toolkit, which provides the manual landmarking functionality and the user interface.

2.1 Install GTK+ 2

The TINA libraries depend on the GTK+ 2 libraries, which must be installed prior to installing TINA. Both the gtk2 and gtk2-devel packages are required: see the documentation for the Linux distribution you are using for instructions on how to install them. Installation of these packages should also result in installation of various other packages (atk, pango, fontconfig, pkgconfig) required by both GTK+ 2 and TINA.

2.2 Install the TINA libraries

Download the latest tina-libs and tina-tools tarballs from `http://www.tina-vision.net/tarballs/`. The TINA Manual Landmark toolkit version 1.3 requires rcbuild005 or greater.

As root, create a directory called Tina6 in /usr/local. Copy both tarballs into the new directory. Then unzip both tarballs using e.g.

```
gunzip tina-libs-6.0rcbuild005.tar.gz
gunzip tina-tools-6.0rcbuild005.tar.gz
```

and then unpack both using

```
tar -xvf tina-libs-6.0rcbuild005.tar
tar -xvf tina-tools-6.0rcbuild005.tar
```

Build the tina-libs libraries first: cd into the tina-libs-xxx (where xxx is the build number) directory, created when the tina-libs tarball was unpacked, and type

```
./configure
make
make install
```

Build the tina-tools libraries after the tina-libs libraries. cd into the tina-tools-xxx (where xxx is the build number) directory, created when the tina-tools tarball was unpacked, and type

```
./configure
make
make install
```

Note that the configure script in the tina-tools directory provides some information about the required libraries: in particular, if you do not have the necessary GTK+ 2 libraries installed (see step 1) it will warn you about this.

2.3 Install Volpack

The TINA Manual Landmarking toolkit requires the Volpack volume rendering library. A version of this library can be found in `http://www.tina-vision.net/tarballs/manual_landmark_toolkit`. Unzip and unpack the tarball (this can be done in any directory, although it is standard practice to put user-installed software in /usr/local), cd into the new Volpack directory created when the tarball was unpacked, and use

```
./configure
make
make install (as root)
```

to build the library. On standard Linux systems the Volpack library and header file will be copied into standard locations (/usr/local/lib and /usr/local/include respectively). The compiler should automatically pick up the location of the header file, and the location of the library will be automatically picked up if /usr/local/lib is specified in /etc/ld.so.conf (the system-wide list of library locations). If it is not, then add /usr/local/lib to the file. Run ldconfig to refresh the list of libraries after the installation of Volpack (or restart the machine).

2.4 Install the Manual Landmarking Toolkit

Download the manual landmarking toolkit (max_planck_toolkit-1.0.tar.gz) from the TINA web-site, place it in a convenient directory (e.g. `/home/my_username/tina_toolkits`), then unzip and unpack it. A new sub-directory will be created: cd into this subdirectory and type

./configure

The configure script will search for the external libraries needed to build the toolkit, warning you about any it fails to find. In particular, if it cannot find the tina-libs or tina-tools libraries and headers, then they can be passed to the configure command using the following flags

–with-tina-includes=PATH
–with-tina-libraries=PATH
–with-tinatool-includes=PATH
–with-tinatool-libraries=PATH

e.g. ./configure –with-tina-libraries=/usr/local/Tina6/tina-tools/lib

If the tarballs were unpacked in /usr/local/Tina6, then the correct locations are e.g.

–with-tina-includes=/usr/local/Tina6/tina-libs-6.0rcbuild005
–with-tina-libraries=/usr/local/Tina6/tina-libs-6.0rcbuild005/lib
–with-tinatool-includes=/usr/local/Tina6/tina-tools-6.0rcbuild005
–with-tinatool-libraries=/usr/local/Tina6/tina-tools-6.0rcbuild005/lib

Finally, type make to build the toolkit.

The TINA Manual Landmark toolkit needs to find the Volpack header and library. On standard Linux systems, these should be located in /usr/local/include and /usr/local/lib once Volpack has been installed. The header file should be picked up automatically by the compiler, and the library location should be automatically identified if /usr/local/lib is listed in /etc/ld.so.conf (and if ldconfig has been run, or the machine rebooted, since Volpack was installed). If these files cannot be located, the build will fail with error messages about functions with names starting with vp e.g. vpRenderRawVolume (these are the functions provided by the Volpack library). In that case, the locations of the Volpack library and header file can be specified manually to the configure command using the flags.

–with-volpack-include=PATH
–with-volpack-library=PATH

The executable file for the toolkit is located in the src subdirectory; alternatively, typing make install will copy it to the bin subdirectory. In either case, cd to that directory and type

./tinaTool

to run the toolkit.

Upon typing ./tinaTool, the top-level TINA tool will appear on the screen. This provides access to various sub-tools that in turn provide access to the algorithmic functionality. The sub-tools of most interest for manual landmarking are the manual landmarking tool itself, the sequence tool (used for loading medical image data) and the Tv tools (used to display graphics). The text window at the bottom of the top-level TINA tool is used to display information and error messages.

3 Tv's and Tv Tools

The TINA image display system is designed to provide the maximum range of configuration options to the user. It consists of two components: Tv's and Tv tools. The majority of the TINA sub-tools will have a choice list at the top of the sub-tool window labelled "Tv:". Each button in the list represents an available graphics channel; however, Tv's are not displayed by default, in order to save space on the screen.

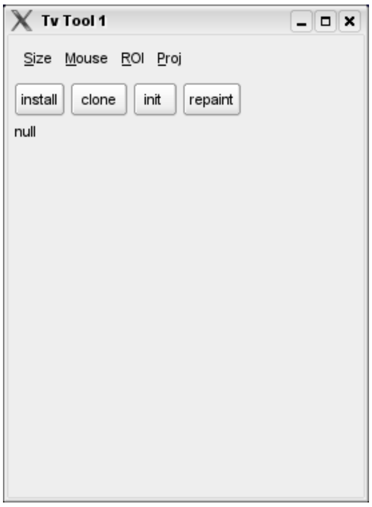

Figure 1: A Tv Tool.

In order to display a Tv graphics channel, it must be associated with a Tv tool:

- Start a new Tv tool by pressing the "New Tvtool" button in the top level TINA tool. A new Tv tool window will appear on the screen, as shown in Fig. 1.
- Select the Tv you wish to display, by selecting on its field in a Tv: list. Note that, since multiple lists are available in various TINA sub-tools, this may be required even if it appears that the desired field is already selected.
- Press the "install" button in the new Tv tool. The title of the Tv tool will change to the name of the Tv it is displaying.

Once a Tv has been installed on a Tv tool, the Tv tool will display the graphics produced by that Tv, automatically updating whenever the graphics change without further user interaction. Tv tools can be minimised or closed at any time without affecting algorithmic functionality. Tv tools can also be freely reassigned, by selecting another Tv and pressing the "install" button again. Note that, when a Tv is installed onto a Tv tool, any other Tv tool displaying that Tv will be un-installed: the title of the Tv tool will change to reflect this.

Each Tv tool has the following menus and buttons:

- Size: set the size of the Tv tool to various default values (256x256, 512x512 and 768x768). Tv tools can also be freely resized by clicking and dragging the corner of the Tv tool window with the mouse.
- Mouse: sets the mouse interaction with the image in the Tv tool.
- ROI: select a region of interest within the image.
- Proj: select the projection function for the Tv tool.
- install: install the currently selected Tv onto the Tv tool.
- clone: install a duplicate of the currently selected Tv onto the Tv tool (allows multiple Tv tools to display the same Tv).
- init: re-initialise the image display to the default viewpoint, removing any translation, rotation or zooming of the image.
- repaint: re-display the image with the current translation, rotation or zooming (used if the image display should become corrupted for any reason).

See the TINA User's Guide [5] for full details.

Each Tv tool also displays two messages: the name of the Tv installed onto the Tv tool, displayed in the title bar; and the current mouse functionality for the Tv tool, displayed just below the button row. The mouse functionality

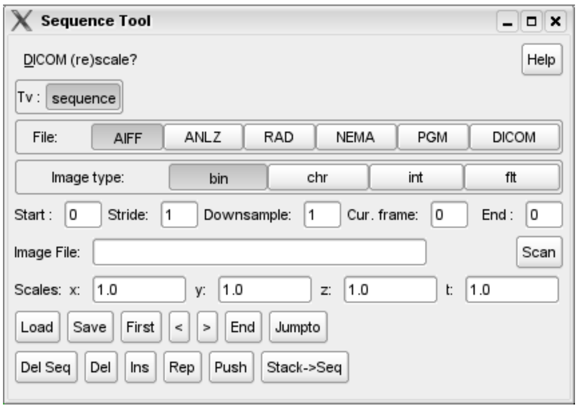

Figure 2: The Sequence Tool, used for loading 3D medical image data.

lists the name of the function type followed by the functionality of the left, middle and right mouse buttons e.g. zoom3D:shift/zoom/rot indicates that the mouse has been set for 3D interaction with the image displayed in the Tv tool, clicking and dragging with the left mouse button moves the image around in the Tv tool, clicking and dragging with the middle mouse button zooms the image, and clicking and dragging with the right mouse button rotates the image in 3D.

4 Loading 3D Medical Image Data: The Sequence Tool

The TINA Manual Landmarking Tool is allows manual identification of anatomical landmarks in 3D medical image data; it was designed for use with micro-CT images of rodent skulls, but can interact with any other tomographic medical image data e.g. MR images. Tomographic medical image data is loaded into TINA using the sequence tool, shown in Fig. 2.

The current sequence can be viewed by installing a Tv tool onto the "Sequence" Tv. This displays the slice specified in the "Curr. frame" field. When a sequence is loaded, the current slice is set to be the first slice in the sequence. Pressing the > button increments the current slice number i.e. moves forwards through the sequence. Pressing the < button decrements the current slice number i.e. moves backwards through the sequence. The "First" and "Last" buttons set the current slice to be the first and last slice of the sequence respectively. To view a specific slice, enter the slice number into the "Curr. frame" field and press the "Jumpto" button.

Individual image slices can be manipulated using the buttons on the bottom of the Sequence tool. The "Push" button copies the current slice to the top of the Imcalc stack (see the TINA User's Guide) where it can be processed or passed to other tools. The "Ins" button takes the image from the top of the Imcalc stack and places it into the sequence after the current slice (if no sequence is loaded, then a new sequence will be created with the image as the first slice). The "Rep" button takes the image from the top of the Imcalc stack and replaces the current slice of the sequence with it. The "Del. seq" button deletes the entire current sequence, allowing it to be replaced with other images from the Imcalc stack. The "Stack->Seq" button will take all of the images currently held in the imcalc stack and use them to create a new sequence (any current sequence is deleted in this process); the images will be placed into the sequence in reverse order i.e. the image on the top of the stack will become the last image in the new sequence.

The sequence tool can also save data to file, although not all file formats are supported (this is to prevent the user from saving sequences where essential header data is not available, or is no longer valid due to processing that has been applied to the images).

In order to load a tomographic image data set:

- Enter the pathname of the image file/files into the "Image file:" field of the tool, either directly or using the file browser started by pressing the "Scan" button.
  - For AIFF, RAD, NEMA and PGM file types the sequence tool will automatically add the correct file name extension (.aiff, .rad, .ani and .pgm respectively), and so the filename should be entered without the extension. However, for Analyze or DICOM files the sequence tool will not automatically add an extension, and so the correct extension must be entered into the filename field. The file-browser started using the scan button will automatically strip the extension from any selected file and so, when loading DICOM files, the extension must be manually re-entered into the filename field.
- Select the format of the image file from the "File:" choice list.
  - For file types (e.g. Analyze) where multiple slices of data are contained within a single file, enter the number of slices in the file into the "End" field. If you are unsure of the number of slices, then enter a number that is too high: the sequence tool will load data until it reaches the end of the file and then reset the "End" field to show the number of slices loaded.
  - For file types (e.g. AIFF) where each slice is contained in a separate file, use the # character as a wildcard. For example, if the directory contains 10 files named im-00 to im-10, enter the pathname of the first file into the "Image file:" field, either by typing or by using the file-browser. Then replace the part of the name that changes between files with the # character, so that it becomes im-##. Enter the replaced part of the filename from the first file into the "Start" field, including any leading zeros (00 in this example) and the relevant part of the filename from the last file into the "End" field (10 in this case). During image loading, the wildcard characters in the pathname will be replaced with the contents of the "Start" field, and the number contained there will be incremented until it is equal to or greater than the "End" field, loading a file each time. If the numbers in the filenames increase by more than 1 between each file, then the "Stride" field can be used to specify the increment. For example, entering im-## into the "Image file:" field, 00 into the "Start" field, 10 into the "End" field and 2 into the "Stride" field will load the images im-00, im-02, im-04, im-06, im-08 and im-10 in that order. Note that there are some restrictions on loading multiple files in this way. The substitution only works for numbers, can only be applied to one contiguous segment of the pathname, and only works in ascending or descending order. In addition, all files must be in the same directory.
- If the sequence is too large to load into memory, the "Stride" and "Downsample" fields can be used to down-sample the data as it is loaded. The "Stride" field down-samples data in the inter-slice direction by skipping over slices as the data is loaded. For example, if the "Stride" field is set to 2, then every other slice will be loaded i.e. the sequence is down-sampled by a factor of 2 in the inter-slice direction. No use is made of the discarded slices: they are never loaded. The "Downsample" field down-samples data within each slice. For example, if the "Downsample" field is set to 2, then each image is down-sampled by a factor of 2 along its x and y axes after it is loaded, and the down-sampled image is entered into the sequence. Linear averaging is used during this process, so that the discarded data is used to reduce image noise.
- Press the "Load" button. Any previous image sequence will be deleted from memory, and the file(s) specified in the "Image file:" field will be loaded in order to produce a new sequence. The "Image type:" choice list will be updated to show the variable type of the images loaded into the sequence. The "Start" and "End" fields will be updated to show the slice numbers found during loading. The "Scales" fields will also be updated to show the size of each voxel, if this information is contained in the image file headers.

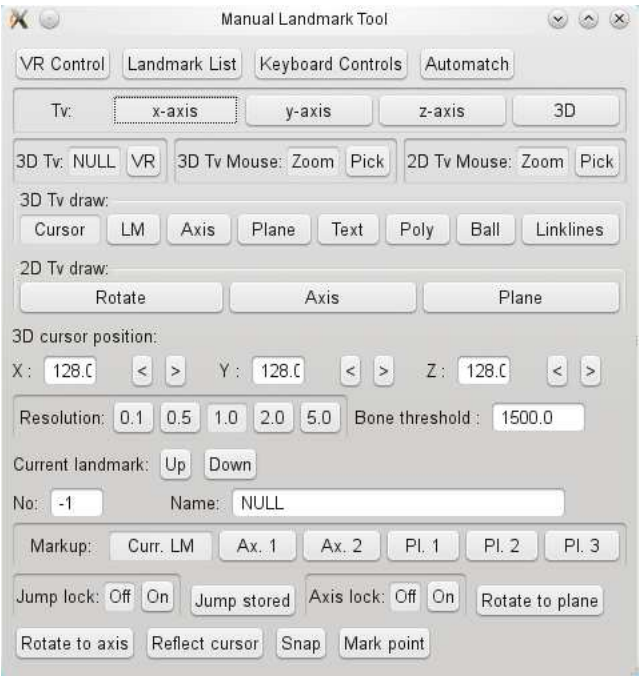

Figure 3: The Manual Landmarking Tool.

In order to save a tomographic image data set:

- Enter the pathname of the image file/files into the "Image file:" field of the tool,
- Select the format of the image file from the "File:" choice list (AIFF, RAD, ANLZ and PGM are supported: NEMA and DICOM are not supported).
  - For file types (e.g. AIFF) where each slice is contained in a separate file, use the # character as a wildcard. For example, if the sequence contains 10 slices, to be saved to files named im-00 to im-10, enter the pathname of the first file into the "Image file:" field. Then replace the part of the name that changes between files with the # character, so that it becomes im-##. During image saving, the wildcard characters in the pathname will be replaced with the slice numbers of the images in the sequence. If the numbers in the filenames increase by more than 1 between each file, then the "Stride" field can be used to specify the increment. For example, entering im-## into the "Image file:" field and 2 into the "Stride" field will save the images im-00, im-02, im-04, etc. Note that there are some restrictions on saving multiple files in this way. The substitution only works for numbers, can only be applied to one contiguous segment of the pathname, and only works in ascending or descending order.
- Press the "Save" button.

5 The Manual Landmarking Tool

The Manual Landmarking tool (Fig. 3) allows morphological landmarks to be identified within any 3D data set loaded into the sequence tool. It provides multiple views of the data; three 2D Tvs, showing orthogonal slices through the sequence, and a 3D Tv showing a volume rendering of the whole sequence. The functionality of the manual landmarking tool is based around the concept of a 3D cursor: a single point within the volume, whose coordinates can be manipulated by the user in a number of ways. The aim is to move the 3D cursor to the point the user wishes to mark, and then to store the coordinates of the cursor in a list of landmark points. When all landmark points have been specified in this way, the list of points can be saved to a file for further processing e.g. Procrustes analysis.

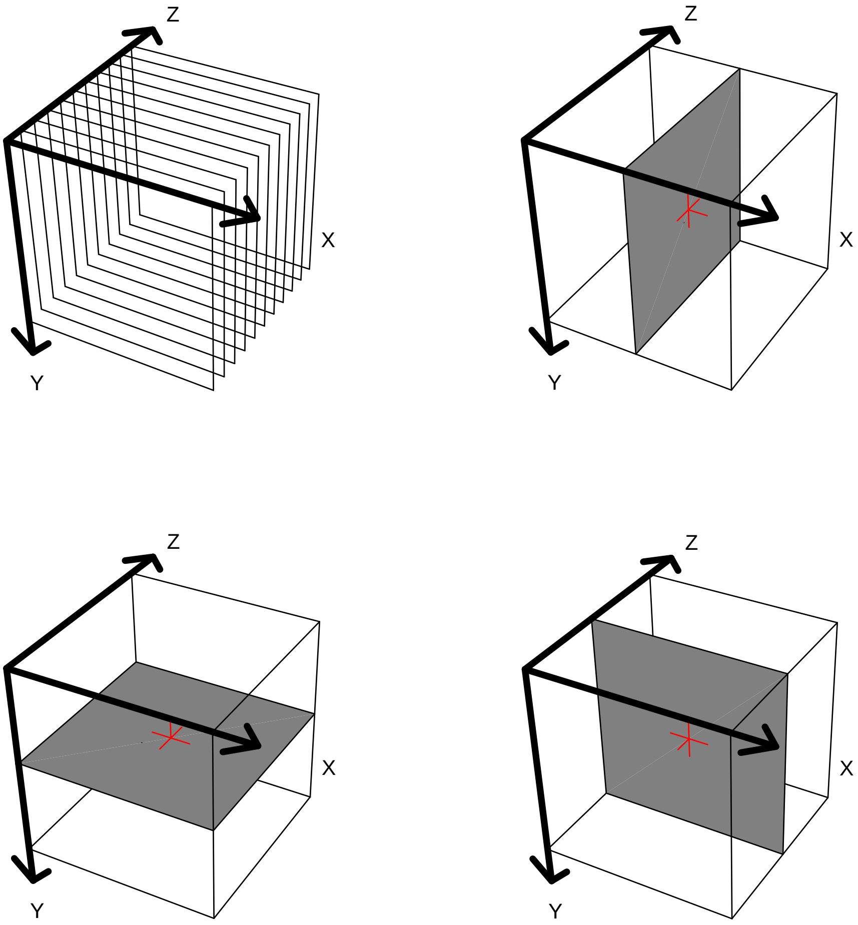

Figure 4: The coordinate system used to refer to the data is shown in (a): the z-direction is the inter-slice direction; the x-direction is the left-right axis of images seen in the sequence tool Tv, and the y-direction is the top-bottom direction of images seen in the sequence tool Tv. The images shown in the three 2D Tvs of the manual landmark tool are generated from orthogonal planes passing through the current 3D cursor position (shown in red), such that the image shown in the x-axis Tv is generated from a plane perpendicular to the x-axis (a), the image shown in the y-axis Tv is generated from a plane perpendicular to the y-axis (b), and the image shown in the z-axis Tv is generated from a plane perpendicular to the z-axis (c).

5.1 Definition of Coordinates

The coordinate system used in the TINA manual landmarking tool is derived from the original data, and relates to the images displayed by the sequence tool as follows: the z dimension is the inter-slice dimension, such that $z = z_{min}$ is the first slice and $z = z_{max}$ is the last slice. Within each image, the x dimension runs from left to right, such that $x = x_{min}$ is the left-hand edge of the image and $x = x_{max}$ is the right-hand edge, and the y dimension runs from top to bottom, such that $y = y_{min}$ is the top edge of the image and $y = y_{max}$ is the bottom. This definition is visible to the user in several places, such as in the definitions of the 2D Tvs and in the landmark coordinates, and is shown in Fig. 4.

When loading large medical image volumes, it may be necessary to use the "downsample" and "stride" fields of the sequence tool, depending on available memory. In this case, it should be noted that the 3D cursor position will always be displayed in the coordinates of the down-sampled volume, but all landmark positions displayed in the Landmark Points dialog box (and save to file using that dialog) will automatically be multiplied by the relevant down-sampling factors so that they are in the coordinate system of the original data. For example, if the original image volume consists of 1000 slices, each 1000x1000 voxels in size, and this is down-sampled by a factor of 2 on

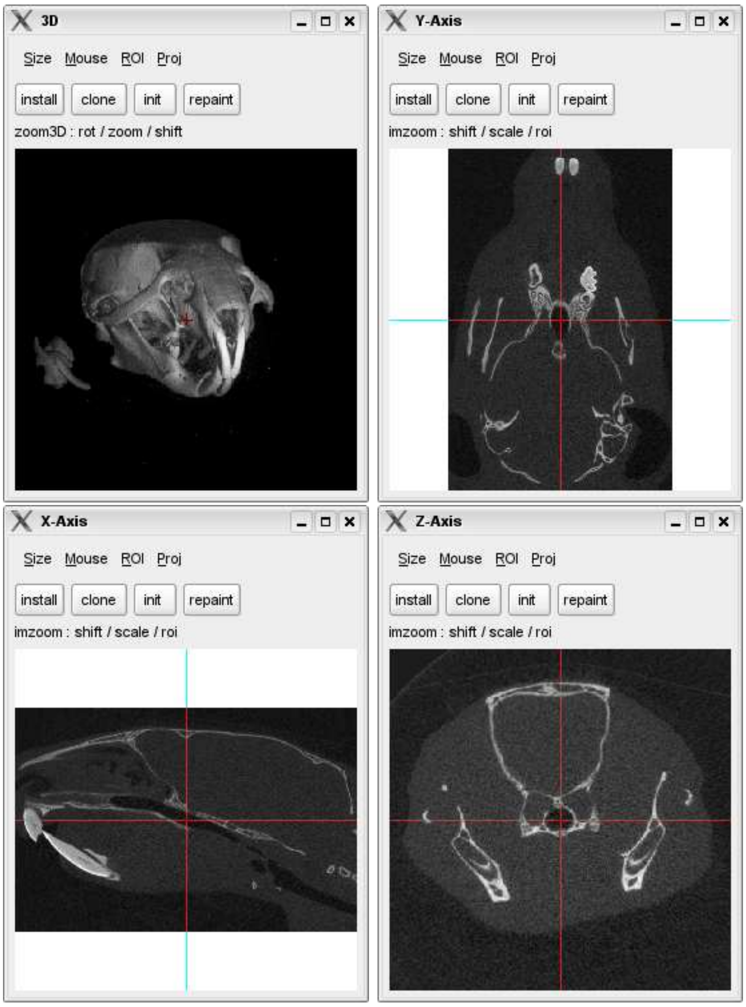

Figure 5: The four Tv's of the Manual Landmarking tool, arranged so that the cross-hairs for the three 2D Tv's are consistent across all three windows.

each axis when it is loaded (i.e. the downsample and stride fields in the sequence tool are both set to 2) then the coordinates of the 3D cursor placed at the centre point of the volume will be (256, 256, 256), and this will be displayed in the X,Y and Z fields of the Manual Landmark tool; however, if this point is marked up as a landmark point, then the coordinates will automatically be multiplied by the down-sampling factors to become (512, 512, 512) before they are displayed in the Landmark Points dialog box or saved to file. This prevents the choice of down-sampling factors from affecting any subsequent Procrustes analysis of the landmark points.

5.2 Image Display

The manual landmarking tool provides four Tvs (see Section 3). Three of these provide 2D views of orthogonal slices through the volume, centred on the 3D cursor. The fourth provides a 3D volume rendering of the volume. The Tvs can also display 3D cursor, landmark, axis and plane points. Figure 5 shows the four Tvs in a recommended arrangement, such that the axes are consistent across the three 2D Tvs.

5.2.1 2D Image Display

The three 2D Tvs display orthogonal slices through the sequence, centred on the current 3D cursor position, which is displayed in each Tv as a large, red cross-hair. Whenever the 3D cursor is moved, the images displayed in these Tvs will be updated to reflect this change. The Tv's are named according to the axis that is perpendicular to the image displayed in the Tv (see Section 5.1), as shown in 4. Therefore, the x-axis Tv displays an image in which the left-right axis is the z axis of the sequence and the top-bottom axis is the y axis of the sequence. The y-axis Tv displays an image in which the left-right axis is the x-axis of the sequence and the top-bottom axis is the z-axis

of the sequence. The z-axis Tv displays an image in which the left-right axis is the x-axis of the sequence and the top-bottom axis is the y-axis of the sequence.

Mouse interaction with the 2D Tvs can be selected using the "2D Tv Mouse:" switch in the Manual Landmark tool. The functions assigned to each mouse button are displayed in the Tv tool, just above the image. When "zoom" is selected, the mouse can be used to manipulate the image. Clicking and dragging with the left mouse button will move the image around in the Tv. Clicking and dragging with the middle mouse button will zoom in or out. Clicking and dragging with the right mouse button will select a rectangular region of interest, and then zoom into that region when the mouse button is released. During the moving or zooming operations, the image will be replaced with a wire-frame in order to increase speed. When "Pick" is selected, the mouse can be used to manipulate the 3D cursor via the 2D Tvs. Only the left mouse button has functionality assigned to it in this mode. Clicking on any point in the image will move the 3D cursor to that point: clicking and dragging will move the 3D cursor interactively. Only the coordinates displayed in the Tv will be altered e.g. the x-axis Tv can be used to manipulate the y and z coordinates of the 3D cursor.

When marking up large numbers of points in multiple images, it is desirable to keep the mouse pointer within the Tvs (i.e. not to have to move it repeatedly back to the Manual Landmark tool window in order to click buttons there). Therefore, the Tvs also provide a set of keyboard shortcuts for commonly used functions:

- Up arrow key: move the 3D cursor position upwards by one increment.
- Down arrow key: move the 3D cursor position downwards by one increment.
- Left arrow key: move the 3D cursor position left by one increment.
- Right arrow key: move the 3D cursor position right by one increment.
- Right ctrl: select "zoom" mode for mouse interactions in all Tvs.
- Right shift: select "pick" mode for mouse interactions in all Tvs.
- Enter: equivalent to clicking on the "Mark point" button in the manual landmark tool i.e. store the current 3D cursor coordinates as the currently selected landmark.

The size of the increment for cursor movement is selected using the "Resolution" choice list in the manual landmarking tool: by default it is set to one pixel.

Note that, in order for the Tvs to receive the keyboard inputs, the window manager focus must be on the Tv. In many window managers, the window that has the current focus is displayed with a coloured title bar: all other windows have a grey title bar. Most window managers provide two modes for focus selection: click-to-focus, in which the user must left-click with the mouse inside a window in order to switch focus to that window, and focus-follows-pointer, in which the focus in on whichever window the mouse pointer is inside. If click-to-focus is selected, then the user will have to left-click on a specific Tv tool in order to send subsequent keyboard instructions to it. Therefore, if significant use is made of keyboard shortcuts, it may be easier to change the window manager focus policy to focus-follows-pointer, so that keyboard instructions are sent to whichever Tv the mouse pointer is inside.

In order to switch to focus-follows-pointer mode in Suse 10.3:

- Click on the start button (in the bottom-left corner of the screen); select "Configure desktop".
- Select the "Desktop" tab from the menu on the left.
- Select the "Window Behaviour" tab from the menu on the left.
- Select the "Policy" drop-down menu in the "Focus" box.
- Select "Focus follows mouse" from the drop-down menu.
- Click the "Apply" button at the bottom of the window.

The window manager focus will now follow the mouse pointer. These instructions are valid for openSuse 10.3 using the KDE desktop. Other window managers may vary: see the instructions for the window manager you are using if necessary.

The keyboard shortcuts can be reassigned to any set of keys (with the exception of a few reserved by the window manager e.g. the print screen key) using the keyboard controls dialog box (see Section 9).

Additional display functionality can be accessed via the "2D Draw" check list in the Manual Landmarking tool. If "Axis" is selected, then any currently specified axis points will be displayed as small yellow cross-hairs in the 2D Tvs. Similarly, "Plane" will display any currently specified plane points as small cyan cross-hairs. Note that these are projected onto the Tvs, so that they are displayed even if they do not lie in the plane of the image. "Rotate" takes any rotation that the user has applied to the image displayed in the 3D Tv and applies it to the images displayed in the 2D Tvs.

New Feature in Manual Landmark Tool v1.2

Manual Landmark Tool v1.1.1 and earlier:

The "Rotate" option affects only the displays in the Tvs i.e. the increment and decrement operations for the 3D cursor still apply to the original axes of the sequence, not the rotated axes of the Tvs. This is true for both the increment and decrement buttons (i.e. the arrow buttons) in the Manual Landmarking Tool window, and the keyboard shortcuts in the 2D Tvs.

Manual Landmark Tool v1.2 and later:

When the "Rotate" option is selected, the increment and decrement buttons (i.e. the arrow buttons) in the Manual Landmarking Tool window still move the position of the 3D cursor along the major axes of the original image volume. However, when the keyboard shortcuts are used in the 2D Tvs, the cursor is moved along the (rotated) axes of the image displayed in the Tv, not along the axes of the original volume. This allows the User to define a preferred orientation using the axis or plane points, rotate the volume to that orientation, and then select extremal points on structures displayed in the 2D Tvs relative to the defined plane or axis.

5.2.2 3D Image Display

The 3D Tv displays a volume rendering of the data loaded into the sequence tool. Since the preparation of this image involves a significant amount of processor time and memory usage, the volume renderer is not activated by default and must be explicitly switched on by the user. This is done using the "3D Tv" choice list in the Manual Landmarking tool: if "NULL" is selected then the volume renderer is switched off. Selecting "VR" will switch on the volume renderer and prepare the sequence for rendering: this may take several tens of seconds to complete.

In order to switch on the volume renderer:

- Select "VR" in the "3D Tv" choice list in the Manual Landmarking tool.
- Select "3D" in the "Tv" choice list of the Manual Landmarking tool, start a new Tv tool, and click "Install" in the Tv tool; the title bar of the Tv tool will change to indicate that it is now displaying the 3D image stream.
- Click the "VR Control" button in the Manual Landmarking tool to start the volume rendering controls dialog box.
- Use the volume rendering controls (see below) to produce a satisfactory rendering.

Mouse interaction with the 3D Tv can be selected using the "3D Tv Mouse:" switch in the Manual Landmark tool. In "zoom" mode, mouse interaction controls the 3D image. Clicking and dragging with the left mouse button will rotate the image. Clicking and dragging with the middle mouse button will zoom in or out (moving the mouse left and right) or rotate the image within the image plane (moving the mouse up or down). Clicking and dragging with the right mouse button will move the image around within the Tv. When "pick" mode is selected, mouse interaction within the 3D Tv controls the 3D cursor. Left-clicking within the 3D Tv will move the 3D cursor to the first bone surface under the mouse pointer i.e. the position of the mouse pointer specifies a vector through the data (depending on the current rotation of the 3D image); the software will run along this vector, starting at the uppermost point from the current view direction, until it reaches a bone surface. The 3D cursor will then be moved to this point on the bone surface. At the current time, the bone surface is defined by a threshold, which the user can set in the "Bone threshold" field of the Manual Landmarking tool. It is anticipated that this will be replaced with something more quantitative in a future version of the software. However, the software also provides a profiling tool to aid the user in selecting a suitable threshold. Right clicking with the mouse within the 3D Tv will produce a grey-level profile along the vector under the mouse pointer, and display it in the Imcalc Graph Tv.

In order to select a bone threshold intensity:

- Start the volume renderer as described above. Using the “Zoom” mouse mode, rotate the image such that a point with a solid bone surface is clearly visible.
- Start the “Imcalc” tool from the top-level tinaTool.
- Click “Graph” in the Tv choice list of the Imcalc tool: start a new Tv tool, and click “Install” in the Tv tool to assign it to the Imcalc graph Tv.
- Select “Pick” mode for the 3D Tv mouse.
- Move the mouse pointer to a location within the 3D Tv that has a clearly visible bone surface.
- Right click with the mouse: a grey-level profile through the image volume, along the vector under the mouse pointer, will be displayed in the Imcalc Graph Tv. The x-axis of this graph represents depth through the 3D image, with 0 being the back of the image and 1 being the front of the image from the current viewpoint. The y-axis represents the intensity (grey level) of the data along the profile.
- Identify the step change in the intensity profile that represents the transition from soft tissue to bone. Several bone surfaces may be visible in the profile; also, be sure to clearly identify soft tissue from background.
- Calculate an intensity value half-way between the mean intensity of bone and the mean intensity of soft tissue.
- Enter the calculated intensity value into the “Bone threshold” field of the Manual Landmarking tool.

As with the 2D Tvs (see Section 5.2.1; the same comments about window manager focus apply to the 3D Tv), several keyboard shortcuts are available in the 3D Tv:

- Right ctrl: select “zoom” mode for mouse interactions in all Tvs.
- Right shift: select “pick” mode for mouse interactions in all Tvs.
- Enter: equivalent to clicking on the “Mark point” button in the manual landmark tool i.e. store the current 3D cursor coordinates as the currently selected landmark.

These can be reassigned to any desired keys using the keyboard controls dialog box (see Section 9).

As well as displaying a volume rendered image of the data currently loaded into the sequence tool, the 3D Tv can display any stored landmark, axis or plane points. The user can choose which points to display using the “3D Tv draw” check list in the Manual Landmark tool. The available choices are:

- Cursor: display a red cross-hair representing the 3D cursor (switched on by default).
- LM: display green cross-hairs for all marked-up landmark points.
- Axis: display yellow cross-hairs for any specified axis points.
- Plane: display cyan cross-hairs for any specified plane points.
- Text: display the number of each marked-up landmark point next to the point.
- Poly: display a line representing the axis and a grid representing the plane.
- Ball: display 3D spheres instead of cross-hairs for the landmark, axis and plane points.
- Linklines: display white lines linking equivalent landmarks on the left and right sides of the sample.

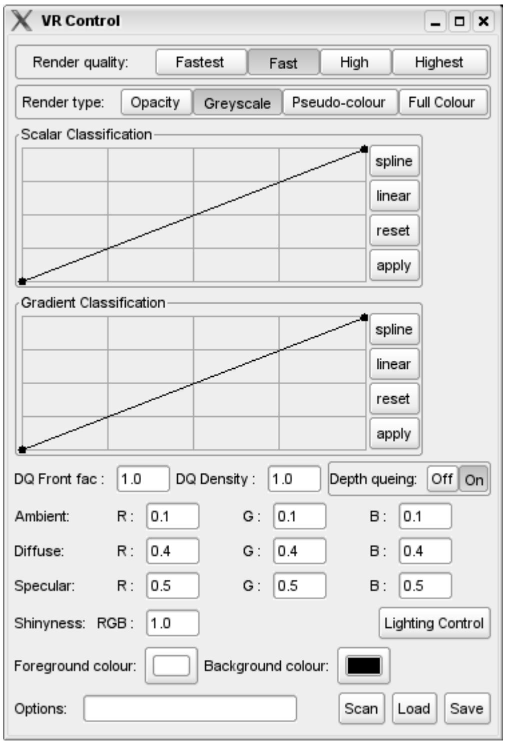

Figure 6: The interface to the volume rendering engine.

New Feature in Manual Landmark Tool v1.2

The "Text" option in the "3D Tv draw" check list of the Manual Landmark tool now displays text labels for any axis and plane points that have been marked. Axis point labels (A1 and A2) are displayed in yellow, and plane point labels (P1, P2 and P3) in cyan. Since a single point might be used simultaneously as a landmark, axis and plane point, the labels are arranged around the point such that they should not overlap.

New Feature in Manual Landmark Tool v1.4

The "Linklines" option has been added to the "3D Tv draw" check list. In many cases the landmark point list will include pairs of points arranged symmetrically around the plane of bilateral symmetry e.g. points on the tips of the coronoid processes of the mandible on the left- and right-hand sides. The user can (optionally) prepare a file listing the landmark point numbers of such pairs (see Section 7 for details of the format and loading of this file). If such a file has been prepared and loaded then, when the "Linklines" option is selected in the "3D Tv draw" check list, white lines will be drawn between these pairs of points in the 3D Tv whenever both points have been manually identified. This can provide a quick visual check of the landmarking process, since the linking lines should all be approximately parallel; if they are not then the user may have made an error (e.g. skipped over a point) during landmarking.

6 The Volume Rendering Interface

The TINA manual landmarking tool uses the Volpack volume rendering library ([4, 3]) to produce 3D views of the data loaded into the sequence tool. In general terms, it is easier to see the general position of a morphological landmark when the data is displayed in 3D, although for maximum accuracy it is important to refine the landmark position by checking the 2D views, since these provide a more direct view of the data.

Pressing the "VR Control" button in the manual landmarking tool spawns a dialog box that provides access to various user-controlled parameters of the volume rendering engine, as shown in Fig 6. The most important of these are the graphs contained in the "Scalar Classification" and "Gradient Classification" fields. Each of these takes the form of a small user-controlled graph with four associated buttons. The curve shown in the "Scalar Classification" graph controls the way in which intensity (grey level) in the original 3D image data loaded into the sequence tool (and displayed on the x-axis of the graph) is converted into the opacity of the corresponding pixel in the volume

rendered image (the y-axis of the graph). The shape of the curve is controlled by a set of points placed within the graph by left-clicking with the mouse or moved by left-clicking and dragging with the mouse cursor placed over an existing point. The "spline" and "linear" buttons set the shape of the curve to be a spline or piecewise-linear curve through the control points. The "reset" button" returns the shape of the curve to the default linear ramp. After setting the curve to the desired shape, the "apply" button must be pressed to pass the new curve to the volume renderer. The effects of these parameters can be seen in Figs. 7, 8, 9, 10 and 11. in general, the curves should both be set to sigmoid functions: using steep sigmoids, as shown in Fig. 9, will produce a surface-rendering style image.

The balance between rendering speed and image quality is controlled by the "Render quality" choice list. These choices control an upper and lower threshold; voxels with intensities below the lower threshold are ignored during rendering, and voxels with intensities higher than the upper threshold are assigned 100% opacity (so that no voxels behind them need to be rendered). The "Fastest" choice sets these thresholds to their most extreme values, so that many voxels are ignored; the rendering is then faster at the expense of lower image quality. The "Highest" choice disables the thresholds completely, so that all voxels are rendered; the rendering is then slow but produces the best image quality. In general the "Fast" choice is the most suitable during mouse interaction with the image displayed in the 3D Tv.

The volume renderer is also capable of producing several different types of image, depending on the selection in the "Render type" choice list. The "Opacity" choice will render an image showing only the opacity of each voxel i.e. with no surface shading, so that the "Gradient classification", lighting and material parameters are all ignored. This provides a transparent view of the data as shown in Fig. 8. The "Greyscale" choice will render an image with both opacity and surface shading, so that the "Gradient Classification", lighting and shading parameters are all applied. However, only the overall intensity of light from each voxel is displayed and so the resulting image is greyscale. The "Pseudo-colour" choice produces a greyscale image but then colours the result using the colour chosen with the "Foreground colour" button, and sets the background to a uniform colour as specified with the "Background colour" button. The "Full colour" choice gives the renderer complete control of image colour, so that coloured lights and material properties can be used (see below); the "Background colour" field can also be used to change the colour of the background in this mode.

Pressing the "Lighting Control" button spawns the Lighting Control dialog box, which provides the user with full control over the lighting parameters used by the volume renderer. See the following section for more details.

The volume renderer also supports depth queing. This function allows a user controlled "fog" to be applied to the volume rendering, which grows thicker with depth through the image. More distant objects therefore appear dimmer, which helps to create a more three-dimensional effect. Depth queing can be switched off or on using the "Depth queing:" buttons, and is controlled by the two parameters "Front fac" (the thickness of the fog at the front of the image) and "Density" (the rate at which the fog grows thicker with depth through the image), according to

$$fog\ thickness = (front\ factor).e^{-density.depth}$$

Both "Front fac" and "Density" must be positive numbers. Changes to the these parameters are not implemented in the rendered image until the next time it is regenerated: the user can force the image to be rendered with the new parameters by clicking the "Repaint" button in the Tv tool displaying the 3D Tv.

When the rendering type is set to greyscale, pseudo-colour or full colour, the intensity gradient of the original 3D image data is used to shade the surface of each voxel. Finer control over the reflectivity of the surfaces can be achieved using the material property parameters contained in the "Ambient", "Diffuse", "Specular" and "Shinyness" fields. The ambient and diffuse fields control how the surface reflects ambient and diffuse light respectively; the red, green and blue components can be set independently. The specular field controls the intensity of specular reflections (i.e. highlights), and again the red, green and blue components can be controlled independently. The shininess field controls the overall shininess of surfaces, for which there is only a single parameter. Valid parameters for the ambient, diffuse and specular rgb components are in the range 0.0 to 1.0: the shininess parameter should be in the range 1.0 to 100.0. The colouration of surfaces will only be visible when the renderer is in full colour mode; in greyscale or pseudo-colour modes the rendered image is produced using only the overall intensity of the reflected light (i.e. the intensity of each of the red, green and blue components combined). Although the Volpack library provides the ability to assign different material properties to different tissues, using the techniques described in [1], the current version of the Manual Landmarking tool assumes a single material type. Changes to the material properties are not implemented in the rendered image until the next time it is regenerated: the user can force the image to be rendered with the new parameters by clicking the "Repaint" button in the Tv tool displaying the 3D Tv.

It is important to note that none of the options in the VR Control or Lighting Control dialog boxes will affect the accuracy of landmark point placement (except to the extent that they affect the user's ability to see where the

landmark point should be placed). When the user selects a point in the 3D image by placing the mouse cursor over that point, and then left-clicking with the "Pick" mode of the "3D Tv Mouse" function selected, the 3D cursor is moved to the first bone surface lying under the point specified by the mouse cursor. However, this is not done using the 3D renderer: instead, it is done by searching through the original 3D image data, as contained in the sequence tool, until a voxel with an intensity equal to or greater than the intensity specified in the "Bone threshold" field of the Manual Landmark tool is found. None of the options contained in the VR Control dialog box is used in this process, and so the user is free to reset them in order to produce the best possible rendering of the data without this process having any effect on subsequent analysis of the landmark points generated using the software.

Significant user experimentation with the options displayed in the VR Control dialog box may be required in order to produce the best possible 3D display of the data. Therefore, the software provides the ability to save/load all of the currently selected volume rendering and lighting options to/from a file. The pathname of the file can be entered into the "Options:" field at the bottom of the VR Control dialog box, either by typing the pathname directly or by using the file browser started with the "Scan" button. The "Load" and "Save" buttons then load or save the options to or from the file.

New Feature in Manual Landmark Tool v1.2

Several example volume renderer options files are available in the extra_files sub-directory of the max-planck-toolkit directory that is created when the Manual Landmarking Toolkit is extracted.

- tmlt_surface_style.txt - the options used in Fig. 9; these provide a surface-rendering style 3D image for many micro-CT scans of rodent skulls.
- tmlt_pseudo_colour.txt - similar to the options used in Fig. 9, but with pseudo-colour rendering enabled and a light-blue background, allowing the User to easily see the opacity of the rendered image.
- tmlt_full_colour.txt - similar to the options used in Fig. 9, but with multiple coloured lights enabled; similar to the options used to produce the image on the front page of this document.
- tmlt_printing.txt - similar to the options used in Fig. 9, but with pseudo-colour rendering enabled with a white background, and with all landmark points displayed as spheres rather than crosshairs: these options are useful for producing images to be incorporated into publications.

6.1 The Lighting Control Interface

The volume rendering engine supports up to six fully configurable light sources. Fig. 12 shows the lighting control dialog box, which allows the parameters of the lights to be specified by the user. The dialog box is created by pressing the "Lighting Control" button in the "VR Control" dialog box.

Each line of the lighting control dialog box controls one of the lights. The buttons on the far left specify whether the light is switched on or switched off. The next three fields (x, y and z) specify the normalised x, y, and z components of the direction of the light; values between -1.0 and 1.0 are valid. The final three components (r, g and b) specify the colour of the light as normalised RGB components; values between 0.0 and 1.0 are valid.

The default is to have a single white light shining down the z-axis; the "Reset to defaults" button resets all parameters to these default values. Changes to the lighting parameters are not implemented until the "Apply" button at the bottom of the dialog box is pressed, and even then have no effect unless the volume renderer is running. If the "Apply" button is pressed with invalid parameters in the dialog box, error messages will be printed to the text window of the top-level tinaTool and any lights with invalid parameters will be disabled. Finally, the lighting control dialog features an emergency mode to ensure that, even if all lights are disabled due to invalid parameters, the default lighting will be applied so that a rendering is still produced.

Note that, if coloured lights are specified with the renderer in greyscale mode, then the rendering will show the intensity of the lighting but not the colour. Switch to full colour mode to display the coloured lighting.

6.2 Limitations of the Volume Renderer

In order for the Manual Landmarking tool to be usable, mouse interaction with the 3D image must be fast enough that the image can be manipulated in interactive time i.e. the renderer must be able to produce several frames per second. This places severe constraints on the amount of processing that can be performed during rendering. Traditionally, landmark identification software has used surface rendering algorithms in order to meet

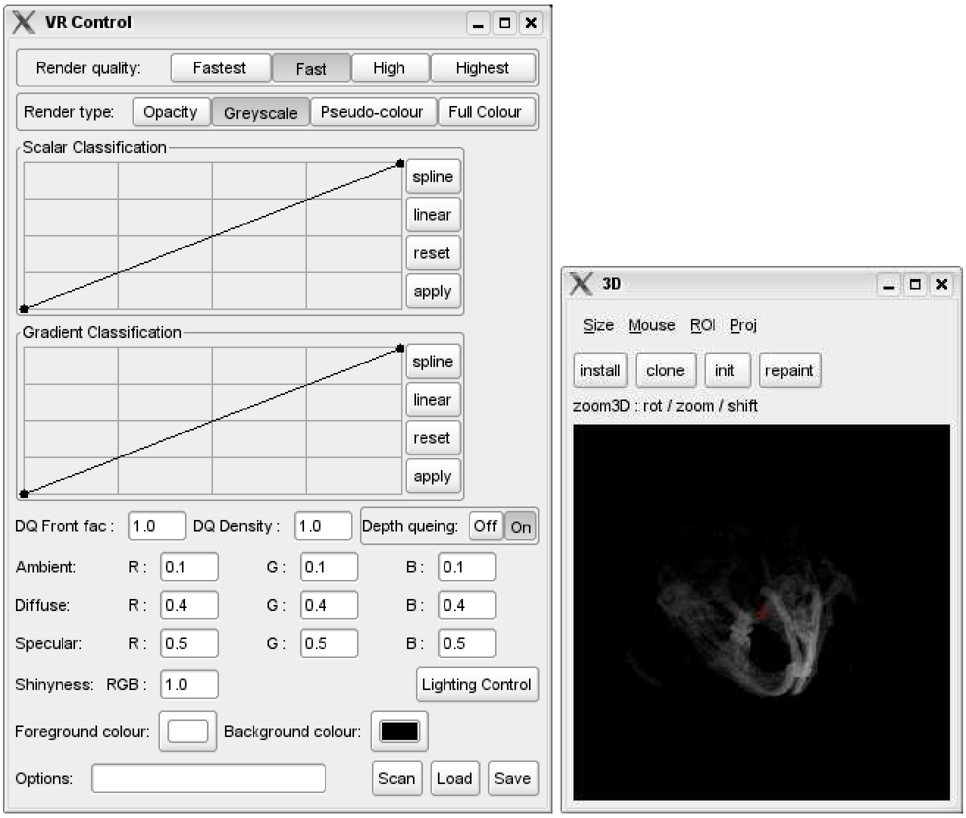

Figure 7: The volume renderer using the default options.

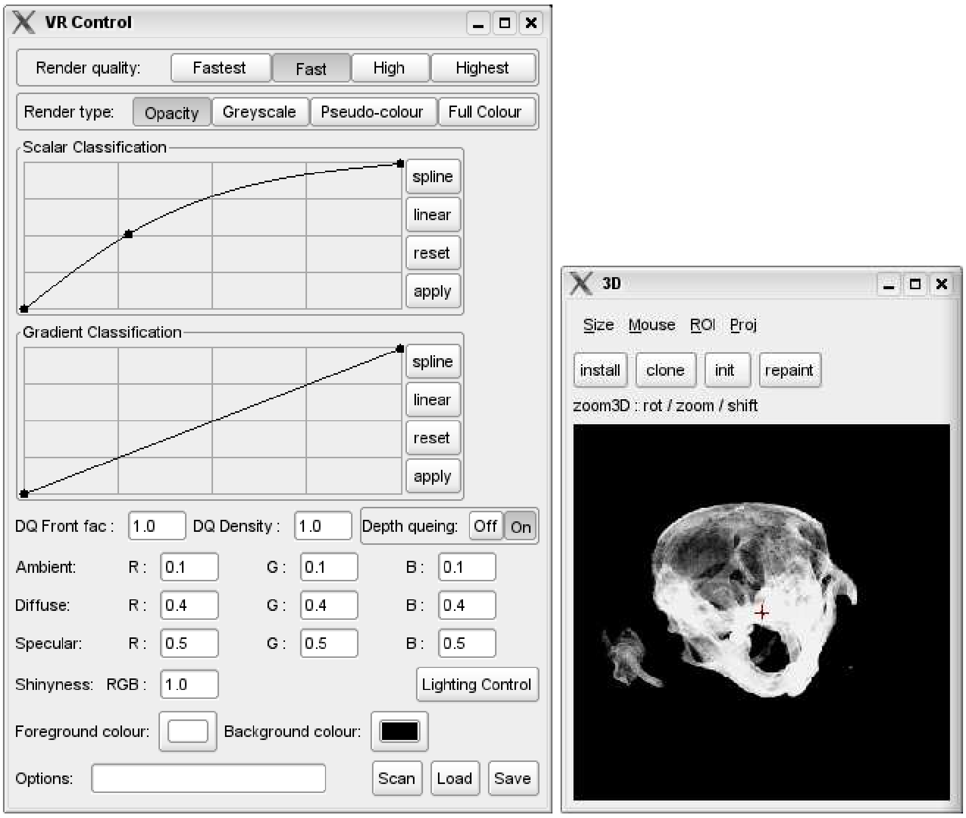

Figure 8: Opacity only volume rendering.

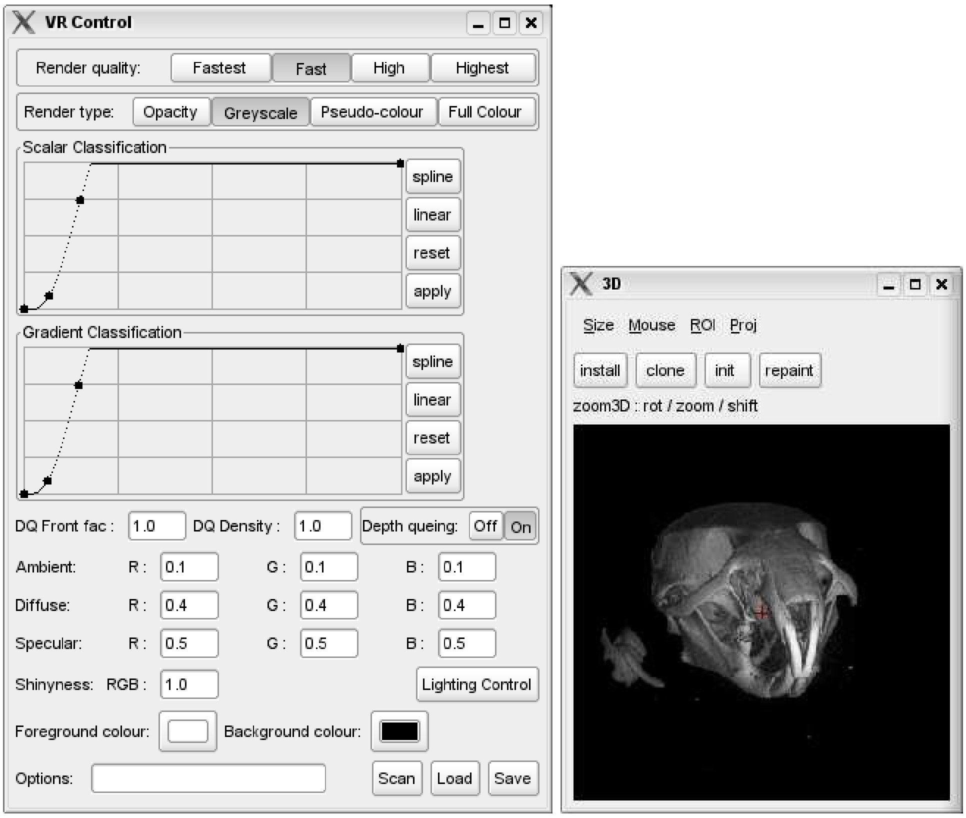

Figure 9: Greyscale volume rendering with options chosen to give a surface rendering style image.

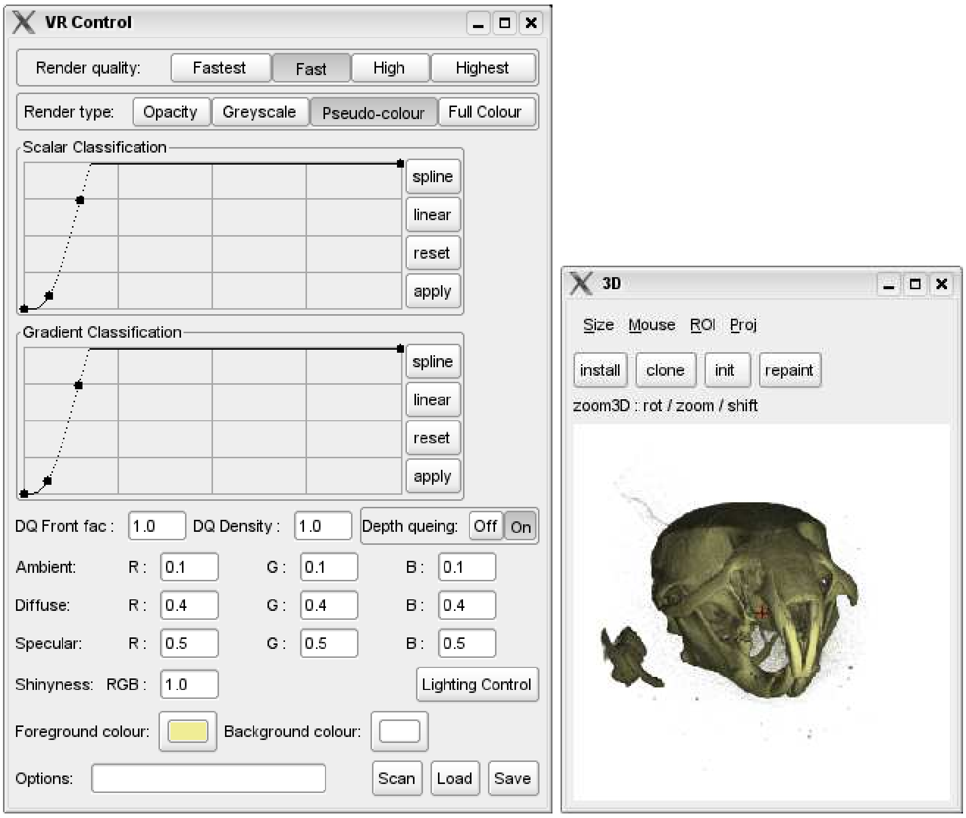

Figure 10: Pseudo-colour volume rendering with options chosen to give a surface rendering style image.

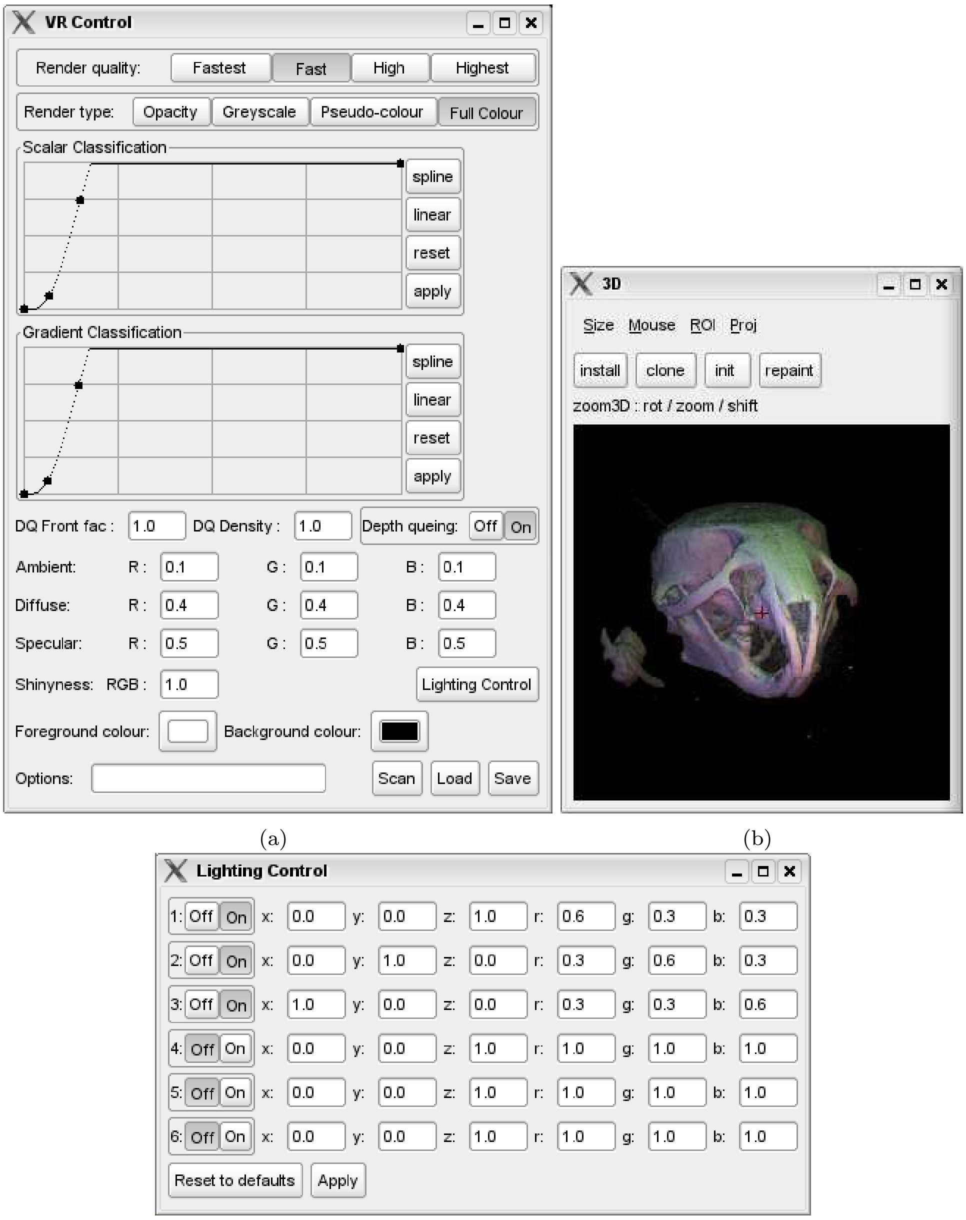

(a) (b)

(c)

Figure 11: Full-colour volume rendering with options chosen to give a surface rendering style image, showing the effects of coloured lighting.

this requirement. Such algorithms rely on identifying a set of points on the bone surface within the volume, tesselating these points to produce a representation of the surface, and then only rendering this surface i.e. only rendering a small fraction of the data. However, this approach has a drawback in that it relies on a specific interpretation of the data i.e. some technique must be used to identify the surface prior to rendering. If, for example, a simple threshold is used, then this is equivalent to the assumption that all bone surfaces in the volume have a well-defined grey-level. At any point where this assumption fails (e.g. where the bone is thin and so obscured by partial voluming, is of low density, or is corrupted by image noise) errors will be generated in the rendering e.g. pseudofenestrations (holes in the rendered bone surface that do not exist in the original data).

The TINA Manual Landmarking tool uses a volume rendering algorithm in order to avoid such problems. Volume rendering produces an image using the entire 3D volume: each pixel in the rendered image represents a vector through the 3D volume, and the renderer runs along these vectors, converting the intensity and intensity gradient of every voxel in the 3D volume into opacity and reflectivity, and combining these together to produce the intensity of the final pixel in the rendered image. The final, rendered image thus provides a more complete view of the data than a surface rendering, at the expense of significantly more processing.

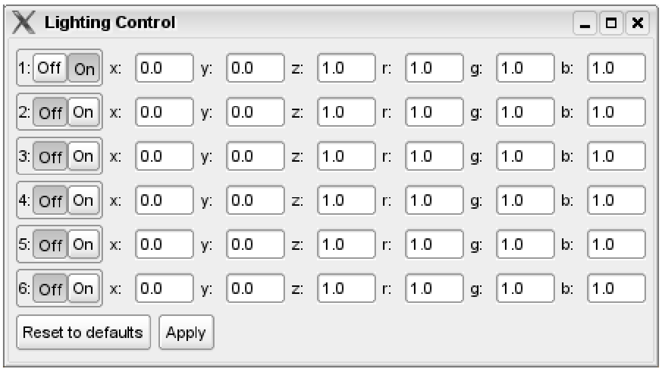

Figure 12: The lighting control dialog box.

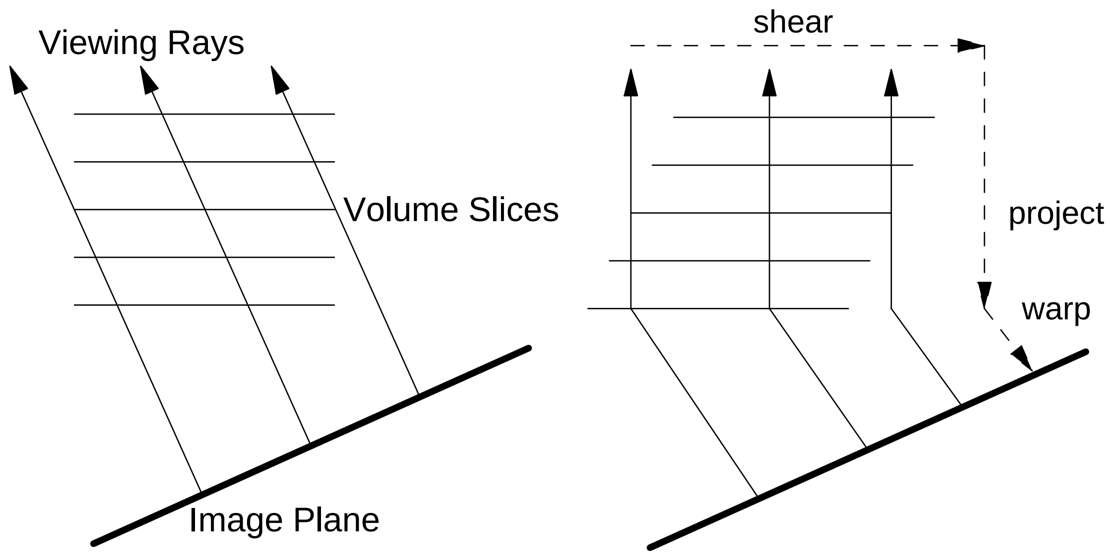

(a) (b)

Figure 13: Volume rendering a 3D data block from an arbitrary viewing direction results in arbitrary angles of intersection between the viewing rays and the slices of the 3D data (a). Shear-warp volume rendering pre-calculates an overall shear of the volume that makes the viewing rays parallel to one axis of the sheared volume (b). The sheared data is then projected along the parallel rays into a buffer, which is finally warped onto the image plane to produce the rendered image. This results in a much more favourable alignment of the data in memory, vastly increasing rendering speed at the expense of a small reduction in the quality of the rendered image.

In order to produce volume rendered images in interactive time, the TINA Manual Landmarking tool uses a fast volume rendering algorithm known as the shear-warp algorithm. The operation of this algorithm is illustrated in Fig. 13. Projecting along vectors from some arbitrary viewpoint would involve complex geometrical calculations in order to find which voxels were intersected by which vectors. Therefore, the shear warp algorithm calculates an overall shearing of the volume that makes all of the vectors parallel to the nearest major axis of the volume. The data is then projected into an intermediate image, which is parallel to one face of the volume, before being warped to produce the final rendered image. This results in a much more favourable alignment of the data in memory, vastly increasing rendering speed at the expense of a small reduction in image quality (introduced by the warping).

Several additional limitations have been implemented in order to maintain rendering speed. The renderer uses a parallel projection, rather than a perspective projection i.e. assumes that all rays from the viewpoint through the data are parallel, rather than diverging. The drawback of this approach is that it can introduce an optical illusion known as the Necker Cube [2] in which, particularly if the image has high transparency, it can be difficult to tell which side of the volume is closest to the viewer. Using renderer settings that minimise the translucency of bone surfaces will minimise this effect.

Furthermore, the lighting model used by the renderer does not take account of radiosity i.e. diffuse lighting

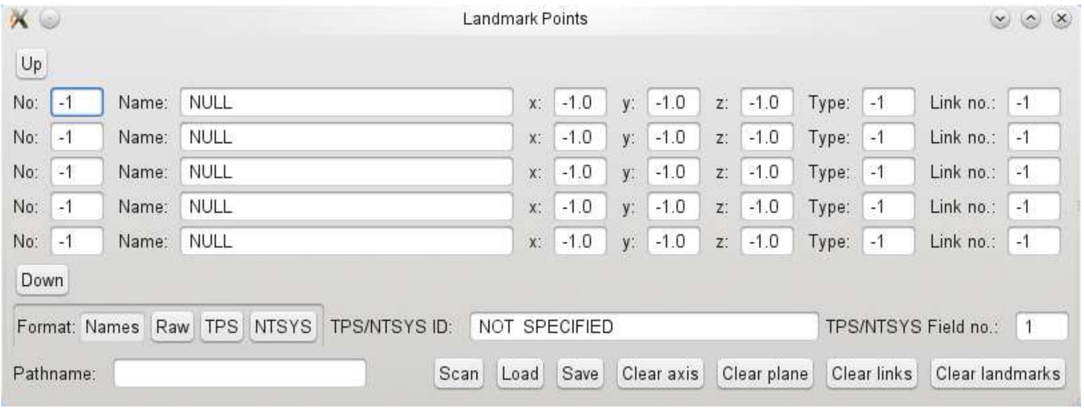

Figure 14: The landmark control dialog box.

reflected from multiple surfaces within the volume. Instead, it only takes account of direct rays of light from the light sources, reflected off of one surface to the viewer. This can cause a characteristic artefact in which rough bone surfaces appear to be speckled with small, dark patches. Correcting this with a more realistic lighting model would increase the processor time requirements to the point where the renderer could not run in interactive time. However, the effect can be minimised with careful choice of lighting parameters (e.g. using a single light shining onto the front of a rodent skull seems to give a good result).

Finally, the TINA Manual Landmarking tool was designed primarily for use on very large micro-CT image volume of rodent skulls (typically several GB in size). In order to make proper use of the accelerated 3D graphics hardware found in most PCs, the data to be rendered must be copied to the video card memory (in order to avoid continual paging of data from system memory to video card memory across the system bus). However, the majority of current video cards lack sufficient memory to hold an entire micro-CT rodent skull volume. Therefore, the volume renderer used in the Manual Landmarking tool does not use graphics hardware; it runs on the CPU and from system memory.

7 The Landmark Control Interface

The TINA Manual Landmarking tool was developed to allow the generation of lists of landmark point positions for use in Procrustes analysis. In this type of analysis, it is essential that each list of landmark points contains the same set of physiological locations in the same order. Therefore, rather than allowing the free-form generation of lists of points, the Manual Landmarking tool requires the user to prepare a text file containing a list of point names and point numbers, referred to as a “names file”, which must be loaded into the tool before point locations can be specified. An example is shown below

An example landmark point names file for mouse skulls:

1 Most anterior point of the anterior palatine foramen, left side.
2 Most anterior point of the anterior palatine foramen, right side.
3 Intersection of frontal process of maxilla with frontal and lacrimal bones, left side.
4 Intersection of frontal process of maxilla with frontal and lacrimal bones, right side.
5 Frontal-squasmosal intersection at temporal crest, left side.
6 Frontal-squasmosal intersection at temporal crest, right side.
7 Center of alveolar ridge over maxillary incisor, left side.
8 Center of alveolar ridge over maxillary incisor, right side.
9 Lateral intersection of maxilla and palatine bone posterior to the third molar, left side.
10 Lateral intersection of maxilla and palatine bone posterior to the third molar, right side.
11 Anterior notch on frontal process lateral to infraorbital fissure, left side.
12 Anterior notch on frontal process lateral to infraorbital fissure, right side.
13 Most posterior point of the anterior palatine foramen, left side.
14 Most posterior point of the anterior palatine foramen, right side.
15 Anterior-most point at intersection of premaxillae and nasal bones, left side.
16 Anterior-most point at intersection of premaxillae and nasal bones, right side.
17 Most infero lateral point on premaxilla-maxilla suture, left side.
18 Most infero lateral point on premaxilla-maxilla suture, right side.
19 Intersection of parietal, temporal and interparietal bones,left side.
20 Intersection of parietal, temporal and interparietal bones,right side.
21 Most inferior aspect of posterior tip of medial pterygoid process,left side.
22 Most inferior aspect of posterior tip of medial pterygoid process,right side.
23 Joining of squasmosal body to zygomatic process of squasmosal, left side.
24 Joining of squasmosal body to zygomatic process of squasmosal, right side.
25 Intersection of zygomatic process of maxilla with zygoma (jugal), superior surface, left side.
26 Intersection of zygomatic process of maxilla with zygoma (jugal), superior surface, right side.
27 Intersection of zygoma (jugal) with zygomatic process of temporal, superior aspect, left side.
28 Intersection of zygoma (jugal) with zygomatic process of temporal, superior aspect, right side.
29 Basion.
30 Bregma: intersection of frontal bones and parietal bones at midline.
31 Intersection of parietal bones with anterior aspect of interparietal bone at midline.
32 Intersection of interparietal bones with squamous portion of occipital bone at midline.
33 Nasion: Intersection of nasal bones, coudal point.
34 Nasale: Intersection of nasal bones, rostral point.
35 Opisthion.
36 Intradentale superior.
37 Most posterior projection of the posterior nasal spine.
38 Most superior point on the external auditory meatus, right side.
39 Anterior edge of the alveolar process where right first molar hits alveolus, right side.
40 Most anterior-medial point on the right carotid canal, right side.
41 Most superior point on the external auditory meatus, left side.
42 Anterior edge of the alveolar process where right first molar hits the alveolus, left side.
43 Most anterior-medial point on the left carotid canal, left side.
44 Anterior nasal spine.
45 Intersection of the right occipital condyle and the foramen magnum, taken at the lateral most curvature, right side.
46 Intersection of the left occipital condyle and the foramen magnum, taken at the lateral most curvature, left side.

The file can be prepared in any text editor, and on each line should contain a point number at the start of the line, followed by one or more spaces or a tab character, then the text description of the point.

If the user wishes to use the “Linklines” option in the 3D Tv (see Section 5.2.2) in order to draw lines linking any pair of points that are arranged symmetrically about the plane of bilateral symmetry, then a file listing these links must also be prepared. Each line of the file contains two landmark point numbers, corresponding to the landmark point numbers in the names file, separated by a tab or one or more spaces. Lines will then be drawn in the 3D Tv between the specified points whenever the “Linklines” option is selected in the “3D Tv draw” check list and coordinates have been specified for both points. The file must have the same filename as the landmark names file

and must have the extension .llf (e.g. if the names file is called points.txt, then the linking lines file must be called points.llf). It must also be placed in the same directory. The linking lines file will then be automatically loaded or saved whenever the names file is loaded or saved. An example is shown below.

An example landmark point links file for mouse skulls (corresponding to the names file above):

```
1  2
3  4
5  6
7  8
9  10
11  12
13  14
15  16
17  18
19  20
21  22
23  24
25  26
27  28
38  41
39  42
40  43
45  46
```

Names files and other files containing landmark points are loaded and saved using the Landmark Control dialog box, which also displays the currently loaded list of landmark points and provides some basic tools to manipulate it. This dialog box is started by clicking on the "Landmark List" button at the top of the Manual Landmarking tool, and is shown in Fig. 14.

In order to load a landmark names file:

- Prepare the names file in advance, using any text editor.
- In the Landmark Control Dialog box, enter the absolute or relative pathname of the landmark names file into the "Pathname" field, either directly or by using the file-browser started by clicking on the "Scan" button.
- Select the format of the file (in this case "Names") from the "Format" choice list.
- Click the "Load" button in the Landmark Control dialog box.

Once a names file has been loaded, the names and numbers of the landmarks can be viewed in the Landmark Control dialog box. The top half of the dialog box shows five landmarks at a time, displaying their number, name, any stored x, y and z coordinates, a "type" field (see below), and a link no. field, which specifies which (if any) other point the point is linked to when the "Linklines" option is used in the "3D Tv draw" checklist.. The user is free to edit any of these fields manually. The Manual Landmarking tool assigns a type of -1 to any point that has not yet been marked up, and a type of 0 to any point that has been manually marked up. The user is free to enter any integer into this field, and so it can be used to hold additional information about the point e.g. if museum specimens are included in the sample group, and parts of the bone surface are missing in some specimens, then the user could assign type 5 to indicate landmark points that have been approximated because the bone surface is missing.

After loading a landmark names file, one landmark in the list is always considered to be the current landmark. During manual landmarking, whenever a point is marked the coordinates of the 3D cursor will be transferred to the current landmark. The current landmark is always the one displayed in the middle of the five rows of landmark information in the Landmark Control dialog box. The user can specify the current landmark (so that points can be marked up in any order) by scrolling up and down through the landmark list using the "Up" and "Down" buttons in the Landmark Control dialog box. In order to avoid having to keep the Landmark Control dialog box open during landmarking, a subset of the dialog is also available in the Manual Landmarking tool, which displays the number and name of the current landmark and has copies of the "Up" and "Down" buttons. However, the current landmark is always kept consistent between these two windows, so when scrolling through the landmark list using

the buttons in the Manual Landmarking tool, the user will also see the list scroll in the Landmark Control dialog box.

The Landmark Control dialog box can also load and save a variety of other file formats, as specified in the "Format" choice list. "Raw" is a TINA-specific, ASCII text format that can contain all of the information from the landmark list. "TPS" and "NTSYS" [1] are standard formats for morphological data. All of these formats are ASCII text files, and so can be viewed or edited in any text editor.

In order to save a landmark list:

- In the Landmark Control Dialog box, enter the absolute or relative pathname of the landmark file into the "Pathname" field, either directly or by using the file-browser started by clicking on the "Scan" button.
- Select the format of the file from the "Format" choice list.
  - Raw files can only hold one list of landmark points. If a Raw file is overwritten, then the previous version of the file will be replaced
  - TPS files can hold multiple lists of landmark points. If landmark points are saved to a TPS file that already exists, then the new set of landmark points will be appended to the existing file. The TPS format assumes that each set of landmarks in the file has a unique identifier: the contents of the "TPS/NTSYS ID" field will be used as the identifier. However, the software does not check whether this is unique in the file i.e. it is up to the user to do this.
  - NTSYS files can hold multiple lists of landmark points. If landmark points are saved to a NTSYS file that already exists, then the new set of landmark points will be appended to the existing file. The contents of the "TPS/NTSYS ID" field will be saved in the file as a comment. The landmark point names will be saved to file as a list immediately preceeding the data matrix; however, NTSYS only supports labels of up to 16 characters, and so the names will be abbreviated if necessary. Users should be aware of this when preparing lists of landmark point names if they intend to use the NTSYS file format. The landmark point coordinates will be saved to the data matrix of the NTSYS file as columns (i.e. each column of the matrix represents a point).
- Click the "Save" button in the Landmark Control dialog box.

Landmark point information can also be loaded from any of these file formats. The "TPS Field No." field allows the user to specify which record to load from a TPS file that contains more than one set of landmark points. Since the software can only maintain one list of landmark points at a time, a conflict may arise in situations where the user attempts to load a file when some landmark information has already been loaded. In such situations, the lengths of the two lists will be compared. If they are the same, then the software will assume that the points are in the same order and will load all of the information contained in the new file into the relevant fields of the current landmark list. If the lengths are different, then the software will either refuse to load the new file, or will discard the current landmark list and replace it with the information from the file, always choosing the option that discards the least data i.e. if the user attempts to load a names file after a TPS or Raw file has been loaded, the software will refuse to load the file; if the user attempts to load a TPS or Raw file after loading a names file, then the names will be replaced with those from the file. However, the Landmark Control dialog box provides a "Clear Landmarks" button that allows the current landmark list to be cleared, and also provides "Clear Axis" and "Clear Plane" buttons allow the user to discard any currently stored axis and plane points (see Section 8.1).

Note that Raw, NTSYS and TPS files do not contain the information from the line linking lines file. However, the user can load this information by loading a names file (with a corresponding linking lines file) after loading a Raw, NTSYS or TPS file, as long as all files contain corresponding lists of landmark points.

8 Manual Landmarking

The aim of the manual landmarking tool is to allow the user to manually specify morphological landmarks in any 3D medical image volume that has been loaded into TINA via the sequence tool. In order to do this, the user must manipulate the position of the 3D cursor until it corresponds to the desired point, and then store the coordinates of the 3D cursor. When data is first loaded, the 3D cursor if initialised at the centre of the volume. Its position is displayed graphically in all Tvs as the red cross-hair. Its coordinates are also displayed numerically in the X, Y and Z fields of the Manual Landmarking tool.

[1]NTSYS read/write has been implemented as of version 1.3.

The position of the 3D cursor can be manipulated in a number of ways:

- Numerical values can be entered directly into the X, Y and Z fields of the Manual Landmarking tool.
- The arrow keys after each of the X, Y and Z fields in the Manual Landmarking tool allow the user to increment or decrement each coordinate of the 3D cursor: the size of the increment can be chosen via the "Resolution" choice list in the Manual Landmarking tool.
- When the 2D Tv mouse interaction is in "Pick" mode, the user can move the large red cross-hair representing the 3D cursor by left-clicking on a position in the Tv, or by left-clicking and dragging. Alternatively, the keyboard shortcuts can be used to shift the position of the 3D cursor (see Section 5.2.1).
- When the 3D Tv mouse interaction is in "Pick" mode, the user can left click on any position in the Tv: the 3D cursor will then be moved to the upper-most bone surface under the mouse cursor. The bone surface is detected using a simple intensity threshold, specified in the "Bone Threshold" field of the Manual Landmarking tool (see Section 5.2.2).

Once the 3D cursor is in the desired position, its coordinates can be stored by clicking the "Mark Point" button in the Manual Landmarking tool. The coordinates will be transferred to the point specified in the "Markup" choice list. If "Curr. LM" is selected, then the coordinates will be transferred to the current landmark i.e. the landmark specified in the "No." and "Name" fields in the Manual Landmarking tool, and also displayed in the middle of the five rows of landmark information in the Landmark Control dialog box. A list of landmark names must already have been loaded in order to do this: otherwise, the software will issue a warning that no landmark list has been loaded. After transferring the coordinates, the software will also increment the current landmark (i.e. move to the next one in the landmark list), so that the user does not need to scroll through the landmark list during landmarking. The other choices in the "Markup" list allow the user to specify axis and plane points (see Section 8.1).

The "Jump lock" switch and the "Jump stored" button allow the user to re-use coordinates of landmarks that have previously been identified. If the user scrolls through the landmark list (using the "Up" and "Down" buttons in the Manual Landmarking tool or in the Landmark Control dialog box) to change the current landmark to a landmark for which coordinates have already been stored, and then clicks the "Jump stored" button, the 3D cursor will be moved to the stored coordinates for the current landmark. If the "Jump lock" switch is set to "On", then this becomes the default behaviour when scrolling through the landmark list: the 3D cursor will automatically be moved to the stored coordinates of the current landmark whenever such stored coordinates exist.

The "Snap" button provides a basic snap-to-grid style functionality. When the 3D cursor is outside the bone, clicking the snap button will move it to the nearest bone surface, as defined by the bone threshold. This function will have no effect if the 3D cursor is inside bone or already on a bone surface.

8.1 Axis and Plane Points

In addition to landmark points, the TINA Manual Landmarking tool supports three plane points and two axis points (these are not displayed in the landmark dialog box). The plane points are used to specify the plane of bilateral symmetry. The axis points are used to specify an arbitrary axis within the 3D image. Both sets of points are used to implement various utility functions that make the manual landmarking process easier. Axis and plane points can be specified regardless of whether a landmark list is loaded or not.

In order to mark up an axis or plane point:

- Move the 3D cursor to the desired point in the 3D image
- Select the desired point in the "Markup" list of the Manual Landmark tool (e.g. "Pl.1" is the first plane point).
- Press the "Mark Point" button in the Manual Landmark tool: this will save the current coordinates of the 3D cursor to the point specified in the "Markup" list of the Manual Landmark tool

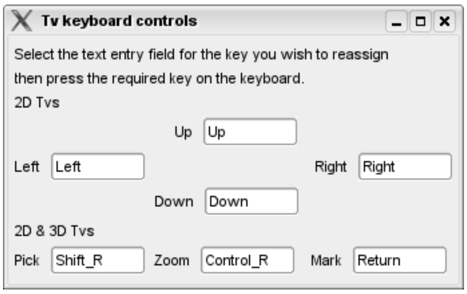

Figure 15: The keyboard controls dialog box.

In order to re-use an existing landmark point as an axis or plane point:

- Scroll through the list of landmark points, either in the landmark control dialog box or in the Manual Landmarking tool.
- Press the “Jump to” button in the Manual Landmarking tool: this will move the 3D cursor to the coordinates of the current landmark point, as specified in the landmark control dialog box and in the Manual Landmarking tool.
- Select the desired point in the “Markup” list of the Manual Landmark tool (e.g. “Pl.1” is the first plane point).
- Press the “Mark Point” button in the Manual Landmark tool: this will save the current coordinates of the 3D cursor to the point specified in the “Markup” list of the Manual Landmark tool

In order to display axis or plane points within the 3D rendering, select the desired options in the “3D Tv Draw:” check list in the Manual Landmarking tool. Plane points are displayed in cyan, and axis points in yellow. As with landmark points and the 3D cursor, the points are displayed as 3D cross-hairs. Any parts of a cross-hair that lie behind a bone surface (as specified by the “Bone threshold” field in the Manual Landmarking tool) from the current viewpoint are displayed in a lower intensity, whilst points that are not behind a bone surface are displayed in a higher intensity; this provides 3D cues to the user, making it easier to see how each point interacts with the bone surface. However, this functionality is disabled during rotation of the 3D rendered image in order to prevent slowdown; during rotation, all points are displayed in a medium intensity.

Plane points are used by the “Reflect cursor” button in the Manual Landmarking tool. Once three plane points have been specified, pressing this button will project the current 3D cursor position through the plane, to an equidistant point on the other side, and reset the 3D cursor to that point. This can be useful when marking up symmetrical structures.

Axis points are used to limit the rotation of the volume rendered image in the 3D Tv. Once both axis points have been specified, press the “Lock Rotation” button in the Manual Landmarking tool. The volume rendered image will then only rotate around an axis parallel to that defined by the two axis points and passing through the centre of the volume. This functionality can be useful in viewing the extremal extents of some structures.

The viewpoint of the 3D Tv can also be aligned using the “Rotate to Axis” and “Rotate to Plane” buttons in the Manual Landmarking tool. Once three plane points have been selected, clicking the “Rotate to Plane” button will rotate the volume such that the viewing direction after rotation is the normal to the plane, using the smallest rotation angle possible. Clicking the button a second time will flip the volume over to give the view from the other side of the plane. Once all axis and plane points have been defined, clicking the “Rotate to Axis” button will rotate the volume such that the projection of the axis onto the plane is aligned with the left-right axis of the 3D Tv, such that using both buttons in combination will completely align the volume. Again, the rotation applied is through the smallest angle possible, and clicking the button a second time will flip the volume over to give the view from the opposite side.

9 Keyboard Controls

As described in Sections 5.2.1 and 5.2.2, a number of keyboard shortcuts are available in the 2D and 3D Tvs, enabling commonly used functions to be applied without moving the mouse cursor away from the Tv tools. These

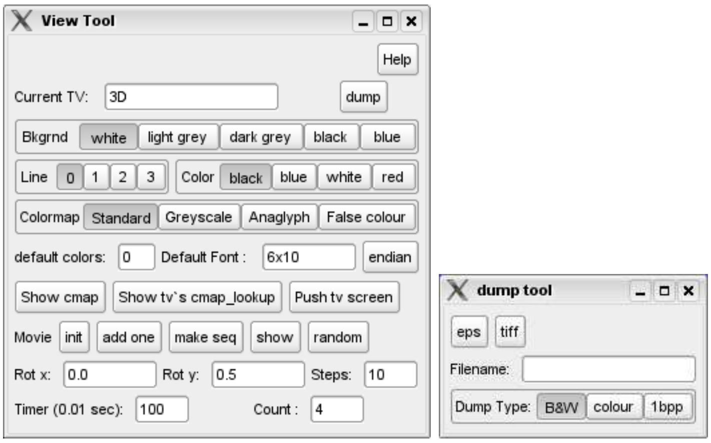

Figure 16: The view tool and dump tool.

can be reassigned to any key (with the exception of a few keys reserved by the window manager, such as the print screen key) using the Keyboard Controls dialog box. This dialog box is started by clicking the "Keyboard Controls" button at the top of the Manual Landmarking tool, and is shown in Fig.15. In order to reassign a key, left-click with the mouse inside the field displaying the keyboard control you want to reassign, and then press the key you want to specify. The name of the key will appear in the entry field. The same set of keys are used across all Tvs.

10 The View Tool

The View and Dump tools provide additional interaction with graphics displayed in Tv tools. See the TINA User's Guide [5] for full instructions on how to use these tools. Two functions of these tools are of particular interest when using the TINA Manual Landmarking tool: the creation of movies from 3D images and the saving of images to files.

The View tool can be used to prepare a movie from any Tv displaying a 3D image (e.g. the 3D Tv of the Manual Landmarking tool), in which the image rotates in 3D around its central point. Each frame of the movie is rendered in turn and stored as a 2D image within the Tv tool; during playback, these 2D images are displayed in the Tv as a slide show. Therefore, no rendering is performed during playback, allowing the movie to play in a much shorter time than it took to render. This can significantly increase the viewer's perception of 3D, and so can be useful in seeing exactly where landmark points are located in the 3D image. The user can specify the angles of rotation, the number of frames in the movie, the time delay between each frame during playback, and the number of times the movie loops during playback. Note that the rotation angles are specified in radians, around the current x-axis (i.e. left-right axis) and y-axis (i.e. up-down axis) of the Tv i.e. they do not refer to the coordinate system used in the Manual Landmarking tool. Once a movie has been created, it is stored in the Tv tool until the user deletes it.

To create a movie in a Tv tool displaying a 3D image:

- Select the Tv for which you wish to produce a movie by clicking on its entry in the Tv list of the tool it is associated with (e.g. click on “3D” in the Tv list of the Manual Landmarking tool).
- Start the View tool by clicking the “View” button in the tinaTool window. The view tool will start, and will display the name of the currently selected Tv in the “Current Tv” field.
- Select the parameters of the movie:
  - Rot x: the rotation to apply about the x-axis of the image (in radians).
  - Rot y: the rotation to apply about the y-axis of the image (in radians).
  - Steps: the number of frames in the movie.
  - Timer (0.01 sec): the time delay between each frame during playback.
  - Count: the number of times to loop through the movie.
- Click “Make seq” to prepare each frame of the movie and store it to the Tv.
- Click “Show” to play the movie in the Tv.
- Clicking “Init” will delete any previously stored movie, allowing a new one to be prepared.

The Dump tool, which is a sub-tool of the view tool, can save the image displayed in any TINA Tv tool as a TIFF or Encapsulated Postscript file; TIFF files can be used to transfer images to Windows machines e.g. for incorporation into MS Word documents, whilst EPS files can be used to incorporate images into documents prepared under Linux (e.g. using Latex).

To save the image displayed in a Tv tool to a file:

- Select the Tv for which you wish to produce a movie by clicking on its entry in the Tv list of the tool it is associated with (e.g. click on “3D” in the Tv list of the Manual Landmarking tool).
- Start the View tool by clicking the “View” button in the tinaTool window. The view tool will start, and will display the name of the currently selected Tv in the “Current Tv” field.
- Click the “Dump” button in the View tool. This will start the Dump tool. Enter a relative or absolute pathname (relative pathnames are relative to the directory containing the tinaTool executable) into the “Filename” field, and select the colour depth using the “Dump Type” field: “B&W” will give a greyscale image, “Colour” will give a full-colour image, and “1bpp” will give a black and white image (which can be useful for saving graphs displayed in the Imcalc Graph Tv).
- Click on either the “TIFF” or “EPS” button to save the image from the currently selected Tv to file in TIFF or Encapsulated Postscript format.

11 Using the Manual Landmarking Tool

A complete set of steps required to specify a set of landmarks is as follows:

- Prepare a landmark names file, specifying the points that you intend to identify (see Section 7).
- Start TINA; start the Manual Landmarking tool.
- Start the sequence tool, assign a Tv tool to the sequence tool Tv (optional; see Section 3). and load a 3D medical image volume (see Section 4).
- Start the volume renderer, using the "3D Tv" switch in the Manual Landmarking tool.
- Start four new Tv tools, and install them on the four Tvs of the Manual Landmarking tool.
- Start the VR Control dialog box and the lighting control dialog box, and manipulate the volume renderer options to produce a good volume rendering (see Sections 6 and 6.1 for suggested settings).
- Use the techniques described in Section 5.2.2 to specify a suitable bone threshold.
- Load the landmark names file using the Landmark Control dialog box (see Section 7).
- The current landmark will now be the first one in the list: manipulate the rotation of the image in the 3D Tv (with the 3D Tv mouse interaction in "Zoom" mode) until you can see the corresponding point on the bone surface.
- Switch the 3D Tv mouse interaction to "Pick" mode, and then left-click on the position of the landmark point in the 3D Tv: the 3D cursor will be moved to that point.
- Check the position of the 3D cursor in the 2D Tvs: if necessary, refine it using the arrow keys in the Manual Landmarking tool, or by using mouse or keyboard interaction with the images in the 2D Tvs (see Section 5.2.1).
- When you are happy with the position of the 3D cursor, click the "Mark point" button in the Manual Landmarking tool (or use the keyboard shortcut in the 2D or 3D Tvs: see Sections 5.2.1 and 5.2.2). The coordinates of the 3D cursor will then be transferred to the current landmark, and the current landmark will then be incremented (i.e. the next landmark in the list will become the current landmark).
- Proceed through the landmark list, marking up each point in turn until the coordinates of all points have been specified.
- Save the landmark list to file using the Landmark Control dialog box (see Section 7).
- At this point the user can load another 3D image volume and repeat the process. Note that the previous landmark list will still be in memory, and so can be used as starting points (if the data sets are similar enough) or can be deleted using the "Clear Landmarks" button in the Landmark Control dialog box, allowing the names list to be re-loaded.

12 Quick Reference

12.1 Sequence Tool

DICOM (re)scale?	Selects the type of scaling tags in a DICOM image (rarely used).
Tv: sequence	The Sequence tool Tv
File:	Selects the file format:<br><br>AIFF: TINA's standard image file format;<br>ANLZ: ANALYZE medical image format;<br>RAD: RAD format;<br>NEMA: Older, pre-DICOM format;<br>PGM: Portable grey map;<br>DICOM: DICOM format.
Image Type:	Displays the variable type of the images in the sequence, and can also be used to cast the current sequence to a different type (by selecting the desired type after loading the sequence):<br><br>bin: binary;<br>chr: short;<br>int: integer;<br>flt: floating point.
Start	The number of the first image in the sequence
Stride	The number of images to skip whilst loading the sequence (e.g. entering 2 means that every other image will be loaded).
Downsample	Down-sampling factor used during image loading (e.g. entering 2 means that each image will be down-sampled by a factor of 2 on the x and y axes during loading).
Cur. frame	The current image in the sequence i.e. the one displayed in the sequence tool Tv.
End	The number of the last image in the sequence.
Image File:	The absolute or relative pathname of the file to load/save.
Scan	Starts a file-browser for the "Image File:" field.
Scales: x,y,z,t	The dimensions of each voxel in the sequence
Load	Load data from the file specified in the the "Image File:" field.
Save	Save the current sequence to the file specified in the the "Image File:" field.
First	Set the first image in the sequence as the current image.
<	Move backwards through the sequence i.e. decrement the current image.
>	Move forwards through the sequence i.e. increment the current image.
End	Set the last image in the sequence as the current image.
Jumpto	Set the image specified in the "Cur. frame" field to be the current image.
Del Seq	Delete the current sequence from memory.
Del	Delete the current frame from the sequence.
Ins	Take the image from the top of the Imcalc stack and insert it into the sequence after the current image.
Rep	Take the image from the top of the Imcalc stack and insert it into the sequence, replacing the current image.
Push	Copy the current image to the top of the Imcalc stack
Stack− >Seq	Take the entire Imcalc stack and produce a new sequence with it (in reverse order).

12.2 Manual Landmark Tool

VR Control	Starts the Volume Renderer Control dialog box, which allows the user to set the various options used to produce the image in the 3D Tv (see below).
Landmark List	Starts the Landmark Points dialog box, which allows the user to load, view and save lists of landmark points (see below).
Keyboard Controls	Starts the Keyboard Controls dialog box, which allows the keyboard controls for interaction with the Tvs to be re-defined.
Tv:	The list of available Tvs in the Manual Landmarking tool: selecting an item in this list will make it available for installation on a Tv tool (see above).<br><br>x-axis: 2D Tv showing images of the z-y plane through the 3D cursor.<br>y-axis: 2D Tv showing images of the x-z plane through the 3D cursor.<br>z-axis: 2D Tv showing images of the x-y plane through the 3D cursor.<br>3D: 3D Tv showing a volume rendering of the data loaded into the sequence tool.
3D Tv:	Switch the volume renderer off/on.<br><br>NULL: switch the volume renderer off.<br>VR: switch the volume renderer on.
3D Tv Mouse:	Switch to control mouse interaction with the 3D Tv.<br><br>Zoom: mouse interaction moves, rotates and zooms the image<br>Pick: mouse interaction picks points on bone surface or displays intensity profiles.
2D Tv Mouse:	Switch to control mouse interaction with the 2D Tvs.<br><br>Zoom: mouse interaction moves or zooms the image.<br>Pick: mouse interaction moves the 3D cursor cross-hairs.
3D Tv draw:	Select how landmark, axis and plane points will be displayed in the 3D Tv.<br><br>Cursor: display a red cross-hair representing the 3D cursor.<br>LM: display green cross-hairs for all marked-up landmark points.<br>Axis: display yellow cross-hairs for the axis points.<br>Plane: display cyan cross-hairs for the plane points.<br>Text: display the number of each marked-up landmark point.<br>Poly: display a line representing the axis and a grid representing the plane.<br>Ball: display 3D spheres instead of cross-hairs for the landmark, axis and plane points.<br>Linklines: display white lines linking symmetrical pairs of points.
2D Tv draw:	Select how the images in the 2D Tvs will be displayed.<br><br>Rotate: display the 2D Tvs using the rotation from the 3D Tv.<br>Axis: display yellow cross-hairs for the axis points.<br>Plane: display cyan cross-hairs for the plane points.
3D Cursor Position:	Displays the coordinates of the 3D cursor.<br><br>X: the x-coordinate of the 3D cursor.<br>Y: the y-coordinate of the 3D cursor.<br>Z: the z-coordinate of the 3D cursor.<br><: decrement the relevant coordinate of the 3D cursor by the number of pixels specified in the “resolution” choice.<br>>: increment the relevant coordinate of the 3D cursor by the number of pixels specified in the “resolution” choice.
Resolution	The number of pixels by which to move the 3D cursor when the arrow keys are pressed.
Bone threshold	Specify an intensity value that corresponds to bone surface.

Current landmark:	The current landmark in the landmark list.<br><br>Up: move to the previous landmark in the list.<br>Down: move to the next landmark in the list.<br>No.: the number of the current landmark.<br>Name: a text description of the current landmark.
Markup:	Choose which point to mark up when the “Mark point” button is pressed.<br><br>Curr. LM: the current landmark from the landmark list.<br>Ax. 1: the first axis point.<br>Ax. 2: the second axis point.<br>Pl. 1: the first plane point.<br>Pl. 2: the second plane point.<br>Pl. 3: the third plane point.
Jump lock:	If this switch is on, then when the selection in the markup choice list is changed (or when the choice of current landmark is changed, if “Curr. LM” is selected in the markup choice list) the 3D cursor will automatically by moved to the stored coordinates for that point (if any).
Jump stored	Move the 3D cursor to the coordinates of the point specified in the markup choice list (if those coordinates have already been marked up).
Axis lock:	Lock the rotation of the image in the 3D Tv so that rotation only occurs around an axis parallel to the axis defined by the two axis points and passing through the centre of the volume.
Rotate to plane	Rotate the image in the 3D Tv such that the view direction is normal to the plane: pressing the button a second time will rotate the view by 180 degrees around the y-axis to show the opposite side of the volume.
Rotate to axis	Rotate the image in the 3D Tv such that the projection of the axis defined by the two axis points onto the plane defined by the three plane points is aligned with the left-right axis of the Tv: pressing the button a second time will rotate the view by 180 degrees around the y-axis of the Tv to show the opposite side of the volume.
Reflect cursor	Project the cursor through the plane defined by the 3 plane points.
Snap	Move the 3D cursor to the nearest bone surface (as defined by the bone threshold; unless it is already inside bone).
Mark point	Save the coordinates of the current 3D cursor position to the point specified in the markup choice list (i.e. the current landmark or one of the axis or plane points). A landmark list must be loaded before landmarks can be marked up.

12.3 Volume Renderer Control Dialog Box

Render quality:	Set the balance of speed vs. rendering quality by ignoring low-opacity voxels: the recommended option is "Fast".
Render type:	Choose the type of rendering.<br><br>Opacity: show an image of opacity (transformed intensity) only.<br>Greyscale: show both opacity and surface shading (transformed gradient).<br>Pseudo-colour: show a greyscale image in which the object is tinted with the "Foreground colour" and the background is filled with the "Background colour".<br>Full Colour: give the renderer full control of image colour (this allows the use of coloured lights and material properties).
Scalar classification:	A user-defined curve controlling the transformation from intensity in the original data to opacity in the rendered image.<br><br>spline: produce a spline curve linking the control points.<br>linear: produce a piecewise linear curve linking the control points.<br>reset: reset the curve to a linear ramp.<br>apply: implement the changes made to the curve.
Gradient classification:	A user-defined curve controlling the transformation from intensity gradient in the original data to surface shading in the rendered image.<br><br>spline: produce a spline curve linking the control points.<br>linear: produce a piecewise linear curve linking the control points.<br>reset: reset the curve to a linear ramp.<br>apply: implement the changes made to the curve.
DQ Front Fac:	The thickness of the depth queing fog at the front of the image (>0).
DQ Density:	The rate at which the depth queing fog becomes thicker with depth through the image (>0).
Depth queing: Off/On	Switch depth queing on/off; depth queing acts like a fog in the rendered image, so that more distant parts of the data are dimmer.
Ambient R/G/B:	The reflection coefficients of surfaces for ambient light (red, green and blue; 0.0 to 1.0).
Diffuse R/G/B:	The reflection coefficients of surfaces for diffuse light (red, green and blue; 0.0 to 1.0).
Specular R/G/B:	The specular reflection coefficients of surfaces (red, green and blue; 0.0 to 1.0).
Shinyness RGB:	The shinyness of surfaces (1.0 to 100.0).
Lighting Control:	Start the Lighting Control dialog box.
Foreground colour:	Set the foreground colour for pseudo-colour rendering.
Background colour:	Set the background colour for pseudo-colour and full colour rendering
Options:	Specify a file from/to which all the options of the renderer can be loaded/saved.
Scan	Start a file browser for the options file.
Load	Load renderer options from the file specified in the "Options" field.
Save	Save renderer options to the file specified in the "Options" field.

12.4 Lighting Control Dialog Box

1-6 On/off:	Switch the light on or off (a maximum of six lights are supported).
x	The x-component of the direction vector of the light (-1.0 to 1.0).
y	The y-component of the direction vector of the light (-1.0 to 1.0).
z	The z-component of the direction vector of the light (-1.0 to 1.0).
r	The red component of the colour of the light (0.0 to 1.0).
g	The green component of the colour of the light (0.0 to 1.0).
b	The blue component of the colour of the light (0.0 to 1.0).
Reset to defaults	Reset all options to the default of a single white light shining down the z-axis.
Apply	Implement any changes to the lighting options.

12.5 Landmark Points Dialog Box

Up — Move upwards in the list of landmark points (the next five rows of fields show the information stored for the landmark points; the middle row of the five is the current landmark).

No: — Landmark point number.

Name: — Landmark point name.

x: — Stored x-coordinate for the landmark point.

y: — Stored y-coordinate for the landmark point.

z: — Stored z-coordinate for the landmark point.

Type: — An arbitrary integer allowing the user to store additional information for the landmark point.

Link no.: — The point that this point is linked to when the "Linklines" option of the "3D Tv draw" checklist is used.

Down — Move downwards in the list of landmark points.

Format: — Choose the type of file to load or save.
Names: a list of landmark point numbers and names (and corresponding linking lines file, if one exists).
Raw: a TINA-specific ASCII text format that contains all of the information stored in the landmark list.
TPS: a standard format for morphological data.
NTSYS: another standard format for morphological data.

TPS/NTSYS ID: — (TPS/NTSYS files) Study name.

TPS/NTSYS Field no.: — (TPS/NTSYS files) TPS and NTSYS files can hold multiple lists of points: this field selects which one to load if there is more than one list in the file.

Pathname — Absolute or relative pathname of the file from/to which to load/save landmark data.

Scan — Start a file browser to select the file to load or save.

Load — Load data from the file specified in the "Pathname" field, using the format specified in the "Format" field.

Save — Save the current landmark point list to the file specified in the "Pathname" field, using the format specified in the "Format" field.

Clear axis — Delete the current axis points.

Clear plane — Delete the current plane points.

Clear landmarks — Delete the current landmark points list.

12.6 Keyboard Controls Dialog Box

Up — Select a shortcut key to move the cursor upwards in the currently selected 2D Tv by the number of pixels specified in the “Resolution” choice list in the Manual Landmark tool.

Left — Select a shortcut key to move the cursor left in the currently selected 2D Tv by the number of pixels specified in the “Resolution” choice list in the Manual Landmark tool.

Right — Select a shortcut key to move the cursor right in the currently selected 2D Tv by the number of pixels specified in the “Resolution” choice list in the Manual Landmark tool.

Down — Select a shortcut key to move the cursor downwards in the currently selected 2D Tv by the number of pixels specified in the “Resolution” choice list in the Manual Landmark tool.

Pick — Select a shortcut key that switches the mouse functions to “Pick” mode for all Tvs.

Zoom — Select a shortcut key that switches the mouse functions to “Zoom” mode for all Tvs.

Mark — Select a shortcut key for the “Mark Point” button (i.e. markup the point currently selected in the “Markup” choice of the Manual Landmark tool with the current coordinates of the 3D cursor and then, if “Curr. LM” is selected, move the current landmark to the next one in the landmark list.

References

[1] R A Drebin, L Carpenter, and P Hanrahan. Volme rendering. *Computer Graphics*, 22(4):65–74, 1988.

[2] R L Gregory. Perceptual illusions and brain models. *Proc Roy Soc Lond B*, 171(1024):279–296, 1968.

[3] P Lacroute and M Levoy. The volpack volume rendering library. `http://graphics.stanford.edu/software/volpack/`.

[4] P Lacroute and M Levoy. Fast volume rendering using a shear-warp factorization of the viewing transform. In *Proc. SIGGRAPH '94, July 24-29, Orlando, Florida*, pages 451–458, 1994.

[5] N A Thacker and P A Bromiley. Tina 5.0 user's guide. `http://www.tina-vision.net/docs/memos.php`.
