# Supplementary material for: TINA manual landmarking tool: software for the precise digitization of 3D landmarks
Source: Front Zool. 2012 Apr 5;9:6. doi: 10.1186/1742-9994-9-6 (PMC3353871; doi:10.1186/1742-9994-9-6)
Supplement: Additional file 3 — Tina 5.0 User's Guide. The file gives additional information on the TINA software in general, including features not used for the landmarking procedure. [file 1742-9994-9-6-S3.PDF]

Tina Memo No. 2005-002
Internal

Tina 5.0 User's Guide

Edited by N.A. Thacker and P.A. Bromiley

Last updated
7 / 12 / 2005

Neuro-Imaging Analysis Centre
Medical School
University of Manchester
Manchester M13 9PL
England

©University of Manchester, England.
Software contributors: Steve Pollard, John Porrill,
Neil Thacker, Stuart Cornell, Julian Briggs, Dave Prendergast,
Richard Lane, Anthony Ashbrook, Beth Vokurka, Tony Lacey,
Paul Bromiley, Marietta Scott, Gio Buonaccorsi, Maja Pokric.

Contents

1 Introduction **8**

1.1 The Main Tool . . . 8

1.2 Infrastructure Sub-Tools . . . 8

1.3 Machine Vision Research Sub-Tools . . . 10

1.4 Medical Image Analysis Sub-Tools . . . 11

1.5 File Handling . . . 11

1.6 Display Selection . . . 11

1.7 Standard File Names . . . 12

2 Tv Tool **13**

2.1 Introduction . . . 13

2.2 The Logical Tv and the Tv Tool . . . 13

2.3 Interaction . . . 14

2.3.1 Mouse Interaction . . . 14

2.3.2 ROI Interaction . . . 14

3 View Tool **16**

3.1 Introduction . . . 16

3.2 Graphics Options . . . 16

3.3 Colour Maps . . . 16

3.4 Graphic Hard Copy Output . . . 17

3.5 Making Movies . . . 17

4 Macro Tool **18**

4.1 Introduction . . . 18

4.2 Button Functionality . . . 18

5 Histogram Tool **20**

5.1 Introduction . . . 20

6 Terrain Tool **21**

6.1 Introduction . . . 21

6.2 Display Options . . . 21

6.3 User Interaction . . . 21

6.4 Functionality 21

7 Mono, Stereo and Sequence Tools **23**

7.1 Introduction 23

7.2 File handling 23

7.3 Display 24

7.4 Mouse Activity 25

7.5 Sequences 26

7.6 Push and Pop 26

8 Imcalc Tool **27**

8.1 Introduction 27

8.2 Mouse Interaction 28

8.3 Stack manipulation. 29

8.4 Basic Image Manipulation. 29

8.5 Basic Algebra 30

8.6 Transendental Algebra. 30

8.7 Standard Noise and Texture Filters. 30

8.8 Numerical differential functions. 31

8.9 Complex Image manipulation 31

8.10 Fourier and related Functions. 31

8.11 Misc. 32

8.12 IP defaults: 32

8.13 Create Tool 33

8.14 CP defaults: 33

8.15 Standard Usage 33

8.16 Non-Parametric Image Subtraction 34

9 Edge Geom Tool **36**

9.1 Introduction 36

9.2 Standard Usage 36

9.3 Edge Geom Tool Buttons 37

9.4 Pick and Mouse Menus 38

10 Corner Tool **40**

10.1 Introduction 40

10.2 Standard Useage 40

10.3 Processing Functions 40

10.4 Pick and Mouse Facilities 41

11 Calib Tool **43**

11.1 Introduction 43

11.2 The Camera Model 43

11.3 Tina Interaction 44
11.4 Input/Output 44
11.5 Pre-Processing 44
11.6 Calibration Methods 45
11.7 Covariance Computation 46
11.8 Calibration Errors 46
11.9 Grid Calibration 47
11.10 Calibrating Arbitrary Stereo Images 47
11.11 Model Based calibration 47
11.11.1 Model Parameters 48
11.11.2 Recommended settings 48

12 PMF Stereo Tool **49**
12.1 Introduction 49
12.2 Initial Matching 49
12.3 Cost Options 50
12.4 Match Support 51
12.5 Choosing Matches 51
12.6 Mouse Activity 51
12.7 Default stereo matching 52

13 Correlation Stereo Tool **53**
13.1 Introduction 53
13.2 Standard Usage 53
13.3 Stereo Correlation Matching 54
13.3.1 preproc params. 54
13.3.2 Correlate. 54
13.3.3 disp drad. 55
13.4 Default stereo matching 55

14 Matching Tool **56**
14.1 Introduction 56
14.2 Standard Usage 56
14.3 Matcher File/View 57
14.4 Geometry 57
14.5 Initialisation 58
14.6 Interactive Matching 58
14.7 Automatic Matching 58
14.8 PWR Params 59
14.9 Match Parameters 59

15 Pairs Tool **60**

15.1 Introduction . . . 60
15.2 Standard Usage . . . 60
15.3 Scene and Model Data . . . 60
15.4 Single Line Functions . . . 61
15.5 Complete Scene Functions . . . 61
15.6 Matched Model Functions. . . . 61
15.7 Dialog Boxes . . . 61

16 Colour Image Tool **63**
16.1 Introduction . . . 63
16.2 Colour Theory 101 . . . 63
16.2.1 Colour spaces . . . 63
16.3 The Tina Colour Tool . . . 65
16.3.1 The Tv List . . . 65
16.3.2 Image Input and Output . . . 65
16.3.3 Pushing and Popping Images to and from the Stack . . . 66
16.3.4 Colour Space Conversions . . . 66
16.3.5 RGB Normalisation . . . 66
16.3.6 Colour Segmentation . . . 67
16.4 Quick Reference . . . 68

17 SmartROI Tool **70**
17.1 Introduction . . . 70
17.2 Graphical Display and Data Selection . . . 70
17.3 Profile Input/Output . . . 71
17.4 Mark-up . . . 71
17.5 Training . . . 71
17.6 Search . . . 72
17.6.1 SmartROI Parameters . . . 72
17.7 Typical Use . . . 73

18 NMR-Segment Tool **75**
18.1 Introduction . . . 75
18.2 Tool Description . . . 75
18.2.1 Multi-D fit . . . 75
18.2.2 EM plot . . . 76
18.2.3 Multi-D Grad Calc . . . 76
18.3 Image segmentation example . . . 76

19 NMR-Analysis Tool **78**
19.1 Introduction . . . 78
19.2 Tool Description . . . 78

19.2.1 stim params 78
19.2.2 gamma params 79
19.2.3 perm params 79
19.2.4 Sequence 79
19.2.5 Perfusion 80
19.2.6 Permeability 80
19.2.7 Test 80
19.3 Sequence images segmentation examples 80
19.3.1 FMRI example 80
19.3.2 Perfusion segmentation example 81

20 Coreg Tool **82**
20.1 Introduction 82
20.2 The Simple Coreg Tool 82
20.2.1 Loading Data 82
20.2.2 Manual Coregistration 83
20.2.3 Automatic coregistration 83
20.2.4 Outputting the Results 84
20.3 Simple Coreg Tool Quick Reference 85
20.4 The AIR Parameters Dialog Box 86
20.5 AIR Parameters Dialog Box Quick Reference 86
20.6 The Advanced Coreg Tool 87

21 SeqROI Tool **88**
21.1 Introduction 88
21.2 Graphics and Splines 88
21.3 The VOL: Buttons 89

22 The DODECANTS Tool **90**
22.1 Introduction 90
22.2 Installation 91
22.3 Use of the DODECANTS Tool 92
22.3.1 Preparation of the Data List 92
22.3.2 Registration 93
22.3.3 Segmentation 94
22.3.4 CSF Volume Measurement 96
22.3.5 KNN Classifier 97
22.4 Quick Reference 98

23 Talairach Tool **100**
23.1 Introduction 100
23.2 Tool Description and Use 100

23.2.1 Prerequisites to using the Altas 100
23.2.2 Obtaining Atlas Descriptions for Single Pixels 100
23.2.3 fMRI functionality 100
23.2.4 Talairach Regions 101

24 Flow Tool **105**
24.1 Introduction 105
24.2 Pre-requisites for Use 105
24.3 Using the Tool 105
24.3.1 Initialise Image Volumes 105
24.3.2 Perform Flow Calculations 105
24.3.3 Regional Histograms 106

25 Cortical Thickness Tool **108**
25.1 Introduction 108
25.2 Tool Description 108
25.2.1 Cortical Thickness Tool 108
25.2.2 Cortical Thickness Parameters 110
25.3 Standard Usage 111
25.4 Histogram Numbers and Labels 111

A File Formats **115**
A.1 AIFF Images 115
A.2 Edge Files 116
A.3 Geometry File formats 116
A.4 Calibration Data 117
A.5 Matcher Files 117
A.6 OLD format 117
A.7 NEW format 117
A.8 Multi-Dimensional MR model 118

Chapter 1

Introduction

Tina has been written to provide a research environment for the machine vision programmer and a repository for good algorithms. It supports a mouse driven front end and well integrated package of vision processes running under the UNIX operating system with Xwindows graphics. Different versions of the same user interface are available via the selection of alternative graphical and widget libraries. The individual modules and display facilities are provided as tools under the control of a parent which also displays diagnostic messages. These sub-tools can be grouped according to two types, graphical infrastructure and research. In this document we give a brief description of the system as a whole, discussing generic concepts, we then go on to describe the sub-tools in more detail.

1.1 The Main Tool

The main tool *tinaTool* is the only one that is visible by default when the tool is started from scratch by typing *tinaTool*. Other tools require button activation for initiation. Previous sequences of button interactions can however be stored by typing " tinaTool -s" and then using the **save** option on the main tool. The sequence of button inteactions are then stored in the "tinaTool.cls" file which can subsequently be replayed using " tinaTool -r". In addition the tool contains a generic macro facility allowing the construction of sequences of button presses from anywhere within the tinaTool system. This is of particular value when used in conjunction with the image calculator (*Imcalc Tool*) and *Sequence Tool*.

1.2 Infrastructure Sub-Tools

Much of the power of the Tina software as a development system comes from the ease with which existing resources can be accessed by the user or the user programmer. The combination of sub-tools, when used together, form a powerful research tool. The mechanism which facilitates this is the use of static data structures, which are globally accesable to the user via button pressing and to the programmer via access functions (described in the programmers guide). The concept of the use of this mechanism follows from the "message board" approach to the construction of complex asynchronous processing. This allows the *user* to investigate the consequences of updating stored datasets using alternative parameters or algorithms in the context of use of that data in a larger system. It also allows the *programmer* to gain rapid access to algorithmic resources already compiled into the tools.

The most important global data structure is the *stack*. The stack is best visualised using the *Imcalc Tool* but can be accessed directly by many other tools via **push** and **pop** buttons. The configuration of the internal data pathway of *tinaTool* can be envisaged as a star, with file tools at the extremities, linked together by the *stack*. This permits data loaded or created in any one tool to be transfered to another. For sub-tools which have several associated static data structures the one placed onto the *stack* will be selected by a choice menu. This mechanism allows, for example, pre-processing of images with the image

calculator before further feature extraction using a sub-tool. For generic exchange of user defined data structures the programmer is referred to the programmers guide section on *serialisation.*

Another feaure of the *tinaTool* system is the use of generic display tools. Data stored in the appropriate static locations will automatically be available to the graphical user interface for interactive manipuation (such as zoom or roam) or display (such as histogramming and surface generation). Many characteristic data interaction tools are available, located within the tools which benefit most from each form of interaction.

The sections below give a brief description of the sub-tools available within the system, more details are given later in this document. The tools shown under *tinaTool* will depend upon the users own configuration in his top level file "tinaTool.c".

- *Tvtool*

  The Tina View tool (*Tv tool*) has been developed to provide a common framework for graphic applications. It is used to interactively display in both 2D and 3D with a wide and flexible range of user definable mouse interactions. For this purpose a large number of functions exist for selecting, manipulating and displaying Tina primatives. This is considered an essential support role for the rapid development of machine vision algorithms. All graphic display in Tina is done using the *Tv tools*. Each applcation tool that uses graphics is able to initialise a *Tv tool* in order to apply its own specific interactions. This is done by first selecting the Tv choice option on the subtool and then installing the selected graphic funtion for the chosen *Tv tool* using the **init** button. A *Tv tool* is not dedicated to one function once intitialised and can at any time be taken over by re initialisation by another sub-tool, this maximises the flexibility of the user environment and minimises the number of *Tv tools* that need to be displayed at any moment in time. The data from a *Tv tool* is not modified on re-initialisation and the *Tv tool* may be re-initialised again for the original purpose if required. *Tv tools* can thus be considered as a floating graphics resource.

- *View* : view_tool()

  Generic manipulation of individual *Tv tool* parameters (colour maps etc.) and postscript (or tiff) output for use in documents is handled by this tool. The Tv acted upon will be the one most recently selected via a sub-tool Tv choice menu (and identified via a static variable). The tool can also be used to produce a buffered sequence rotations on data which can then be used for realtime visualisation of 3D data.

- *Macro* : macro_tool()

  This tool supports sorage and execution of button presses with tool reconfiguration capabilities. It is designed for use with debugging (eliminating all that tedious interface re-positioning which preceeds useful work) and sequence analysis (allowing single image analysis processes to be applied to whole sequences or volumes).

- *Stack*: raw_input_tool()

  This tool is provided to allow reading of arbitrary (uncompressed) data, it supports various data types and endian and signed data alternatives. Data is loaded directly into the *stack* for view in the image calculator (see below). With practice, images of unknown origin can be loaded by a process of trial and error in order to determine key variables.

- *Mono*: mono_tool()

  The *Mono Tool* is provided to handle generic file manipulation and display functions for grey level image and edge based data structures. It controls a single Tv tool for *mono* image display of the global mono data structures and has **push** and **pop** access to the *stack*.

- *Stereo*: stereo_tool()

  The *Stereo Tool* is provided to handle generic file manipulation and display functions for stereo image data and derived geometric features. It controls 3 Tv tools for *left right* and *threed* data display of the global left and right data structures and has **push** and **pop** access to the stack.

- *Sequence*: seq_tool()

  The Sequence tool is provided to handle the construction manipulation and processing of temporal, volume or multi-spectral sequences of images. The tool not only provides a means of interacting

with the image sequence but also provides an infrastructure with which temporal algorithms may be developed. It has an associated *Tv* and interacts directly with the stack.

- *Imcalc*: imcalc_tool()

  The image calculator (or *Imcalc Tool*) is a stack based image processing tool designed to be used as a general purpose image processing algorithm development tool and image preprocessor. It's main use is for algorithm evaluation but also has uses for general purpose data manipulation and display. The tool must be used in conjuction with the *Stereo* or *Mono* tools for input and output via stack **push** and **pop** manipulation. It has 4 *Tv tools* associated with it for stack and memory displays and graphical output such as profiles and histograms.

1.3 Machine Vision Research Sub-Tools

The tools contained in this section are less fixed in nature and may not have yet been released for use external to the group. These tools are in a state of flux and may be subject to change at any time, depending upon research requirements. We endevour to keep the most recent versions of proven algorithms up-to date but there is often a lag between the current reseach and inclusion of new techniques. Such techniques will however generally be available as executable demonstrations.

- *Calib*: calib_tool()

  This tool is used for the calibration of images via the estimation of the parameters of projective camera models. It controls a single graphical Tv structure and has access to the global stereo calibration structures via **get calib** and **set calib** buttons.

- *Edge Geom*: edge_tool()

  This tool is an iterface to the routines in Tina system for performing edge detection, edge based stereo processing and recovery of wire frame geometrical scene descriptions. The tool must be used in conjuction with the *Stereo* or *Mono* tools for input output and display and operates on the *mono*, *left* and *right* image global data structures, which can be viewed on their associated Tvs.

- *Corner:* corner_tool()

  The *Corner Tool* is an iterface to the routines in Tina system for performing corner detection and corner based stereo and temporal matching. The tool must be used in conjuction with the *Stereo* or *Mono* tools for input output and display and operates on the *mono*, *left* and *right* image global data structures.

- *PMF Stereo Test*: stereo_test_tool()

  This tool provides an iterface to Tina subroutines for the use of edge string based stereo algorithms. This tool should be used in conjunction with the *stereo* sub-tool and operates on the *left* and *right* image global data structures.

- *Correlation Stereo*: st_corr_tool()

  The *Stereo Corr Tool* provides an iterface to Tina subroutines for the use of image correlation based stereo algorithms. This tool should be used in conjunction with the *stereo* sub-tool and operates on the *left* and *right* image global data structures.

- *Pairs:* pairs_tool()

  The pairwise tool provides an iterface to the Tina subroutines for generic object recognition of 2D projected shape via the use of pairwise geometric histogrammes. This tool should be used in conjunction with the *Mono* sub-tool and operates on the *mono* global data structures.

- *Matcher:* matcher_tool()

  The matcher subtool provides an interface to the Tina subroutines for the location of 3D wireframe objects. It has associated with it additional Tv tools for scene and model display. This tool should be used in conjunction with the *stereo*, *edge* and *corner* tools and operates on the left and right image global data structures.

1.4 Medical Image Analysis Sub-Tools

These tools are intended for the quatitative analysis of medical images. All of them have either been used, or are being used, in medical research projects.

- *NMR-Segment*: nmr_segment_tool()

  This tool has been developed to identify tissues in MR data sets. The approach uses EM modelling based upon Bayes statistics. Its is expected to be used in conjuction with the *Sequence* and *Imcalc* tools.

- *NMR-Analysis*: nmranalysis_tool()

  This tool has been developed for the analysis of temporal MR data sets, such as BOLD, Perfusion and Permeability analysis. Its is expected to be used in conjuction with the *Sequence*, *Imcalc* and *Coreg* tools.

- *Coreg*: coreg_tool()

  This tool is used to co-register and reslice, either manually or automatically, MR data sets. It is designed to be used in conjunction with the *Sequence* tool.

- *SmartROI*: sroi_tool()

  The *SmartROI Tool* is designed to support the automatic location of simple polygonal boundaries in grey level data. The techniques are based on eigen vector approximation to sampled data. The tool can be used in conjuction with the *Imcalc, Sequence* and *Mono* tools. Located structures can be used to define local regions away from the main control points using an method of affine transfer.

- *SeqROI Tool*: seqroi_tool()

  This tool is provided for manipulation, visualisation and storage of 3D volumes of interest. It allows the editing of a set of closed 2D spline curves which can be viewed in the *threed* Tv and apllied as regions of interest in other Tv's. The tool also has use for maual markup of structures.

1.5 File Handling

File i/o facilities typically have three components:

- a selection box to specify file type
- buttons for performing input and output
- directory and basename specification strings

The user sets the directory and basename strings, chooses the appropriate file type and then performs i/o by pressing the appropriate button.

1.6 Display Selection

In general some of the following three facilities will be available

- a selection box to activate Tv control initialisation
- selectors for the current display of each Tv tool
- selectors for the current "pick" and "mouse" facilities of each Tv tool

It is recommended that the section of the user guide for the *Tvtool* and *View* sub tools be read fully.

1.7 Standard File Names

A number of standard file name extensions are assumed by the Tina system when reading and writing to disc. This allows a single image pathname to be used to generate multiple files for globally defined data (see Infrastructure Sub-Tools). File name extensions are delimited by the character "." and these should not be used elsewhere in filenames. Here we describe only the basic file types, other tool specific extensions (such as those used in the *Matcher*) will be described in the relavant sections of this guide.

Images are stored in AIFF (AIVRU Image File Format) in files with the "aiff" postfix. In addition Tina is often used to deal with image pairs (via the *Stereo* subtool), this gives rise to the additional postfix of "l" or "r", eg:

```
widgets.l.aiff
widgets.r.aiff
```

for the stereo pair of "widgets" images.

Edge data, 2D and 3D data are stored separately as:

```
widgets.l.edges
widgets.l.poly
widgets.poly
```

note that the "l" extension is dropped for the 3D case.

Camera geometry is also associated with each view projection on an individual basis as:

```
widgets.l.cam
widgets.r.cam
```

These nameing conventions are used only for convenience and the user can of course use other root names if so required provided that the expected extensions are retained. This will of course require additional user interaction when reading the data.

Tools designed to read and write multiple images include numeric fields which can be substituted into locations in the file name specified by thw "#" and "?" symbols. These tools also support the "*" wildcard.

Chapter 2

Tv Tool

2.1 Introduction

The *Tv Tool* provides a common framework for interactive graphics for use during algorithm development and demonstration. It is used to display image 2D and 3D graphics data. The software has been designed to support straight forward extension and modification by the user programmer. By isolating system dependant graphics code, and providing a protective layer of graphics routines, it is hoped that this software will support portability and upward extendability.

All graphics display in Tina is done via the *Tv Tool*, each application tool that has graphics display in the Tina system has associated *Tv Tool*'s. The *Tv Tool* is resizeable, either by dragging at the corners of the tool or via use of the "Size" menu. 3D graphics can be displayed either with orthographic or perspective projection as required ("Proj" menu). Data can also be inverted by a 180 degree rotation in either 2D or 3D. During data manipulation (eg rotation) only a subset or "skeleton" of the entire data structure may be displayed. This is purely for convenience and the entire data set will be fully displayed at the end of the interaction.

2.2 The Logical Tv and the Tv Tool

The programmer works with a logical display device called a Tv. Tools that require graphical display devices can either reuse existing display devices (via calls to the appropriate pointer access functions) or must provide their own Tv device. During program execution a Tv is created using the *New Tv Tool* button of the main tool. Within reason any number of Tv's can be created. Tv's support the following button proceses. Tv's support both transient and retained graphical facilities. Transient graphics are lost following zooming or other repaint facilities, while retained graphics are not. Generally, transient graphics will not be displayed on cloned Tv's.

- **install**. The user associates the most recently selected *Tv tool* from a subtool selection menu to a logical display device.
- **clone.** Multiple copies of the same Tv can be installed on different *Tv Tool*'s. These copies are not accessable from the main application program and can be therefore used to store graphical results for visual comparison.
- **init**. An aplication can supply an initialisation function which selects a default geometry for display (eg; an image can be scaled to fit the display screen). Using this button will return the displayed data to the standard projection after zooming or rotating.
- **repaint**. When the screen is exposed or resized repaint routines are called to refresh the display appropriately. Users can invoke a repaint directly by pressing this button.

Interaction with the tool takes place through the mouse (assumed to have three buttons). The mouse generates 9 different event types from the combinations of these buttons with up, down and drag.

2.3 Interaction

Experience has allowed us to identify four types of useful activity, *roi* (region of interest), *zoom*, *pick* and *mouse*. The first of these can be selected from the **ROI** menu, the others can be individually selected from the *Tv Tool* **mouse** menu. When an activity is selected a brief description of the functions asociated with the three buttons is displayed in the information panel of the tool.

2.3.1 Mouse Interaction

- **zoom**. Zoom mode allows the data to be shifted scaled and rotated in 2D and 3D by dragging the mouse across the display with a button pressed. The appication selects the modes for zooming according to data type at the time when the tool is installed. The button functions are:

```
left drag   : up/down/left/right = image shift.

middle drag : left/right = zoom in/out (from centre)
              up/down = rotate display about view direction.

right down  : up/down = 3D rotation about vertical display axis.
```

- **pick**. This sets up the basic functionality to choose items from the display for mainpulation and processing. Selected ietms are stored in a "pick-list" which is globally available to the software. The choice function and pick function are set by the application according to data type.

```
left down and drag   : Highlight current choice.

left up              : select curent choice and add to pick-list

middle down          : apply selected funtion to pick-list data

right down           : cancel pick-list
```

- **mouse**. This is intended to be a programmer defined function, and the behaviour will vary depending upon which sub-tool the Tv is installed with.

2.3.2 ROI Interaction

- **rect**. Roi mode allows rectangular regions of interest to be selected for processing. The button functions are:

```
left down   : Choose upper left of region.

left drag   : Drag to lower right corner of region.

left up     : set roi.

middle down : toggles display of current roi.

right down  : Display the current roi.
```

- **poly**. Roi mode allows polygonal regions of interest to be selected for processing. The button functions are:

```
left down   : Add new point to polygon.

middle down : Close and display polygon.

right down  : Start new polygon.
```

- **point**. Roi mode allows polygonal regions of interest to be edited. The button functions are:

```
left down   : move nearest point on polygon.

middle down : delete closest point on polygon.

right down  : add new point to polygon and join to the two closest.
```

- **global**. Roi mode allows polygonal regions of interest to be modified. The button functions are:

```
left down   : translate polygon.

middle down : scale polygon.

right down  : rotate polygon.
```

Chapter 3

View Tool

3.1 Introduction

The *View Tool* is a generic Tv control panel for the control of graphics parameters on the most recently selected Tv as

displayed at the top of the tool. The tool supports various colourmap display modes and can be used to modify graphics display variables including fonts.

3.2 Graphics Options

The background can be set to the required colour by the selection of the following options.

`white,  light grey,  dark grey,  black,  blue`

The line width of displayed geometrical primitives can be set to either of the following (in pixels).

`0,  1,   2,  3`

The default line colour can be set to

`black, blue, white, red.`

3.3 Colour Maps

The display colour map can be selected from the following alternatives;

`standard,   grey scale,  anaglyph, false colour`

These maps are avaialable as either Psedo Color or True Color depending on whether the X server is in 8 bit or 16 bit colour mode or above (increasingly, modern computers are no longer resricted to 8 bit graphics). The “grey scale” option is intended for cases where better image definition is required with 8 bit graphcs than is available with the **Standard** colour-map. **False colour** is intended for displaying images with quantitative grey level values. **Anaglyph**s are provided for stereoscopic display and display of complex images (ie: two value). Tina does not yet support full colour dislays of, for example, RGB’s as there is no need for the algorithms currently supported. The **default colours** variable specifies the number of colours to be copied from the system colourmap. Sensible window managers will not switch

back to the system colourmap when it is included as a subset of the tool colour map in this way. Very large values of this variable (ie: greater than 72) may move the graphical colours of the standard colour maps into the red overlay plane used for temporary graphics. This does not however, cause any undue execution errors.

The colour map can be interregated and examined via the use of the **Show cmap** and **Show tv's cmap_lookup** functions. Most windows managers runnning with 8 bit colour include an "xcmap" utility which is eqivalent. The current Tv screen can be pushed directly into the *Imcalc Tool* if necessary, though this is not reccomended as a stage in data processing.

The default font for the current Tv can be changed. If a wildcard is included (ie: *) the nearest suitable font will be chosen.

Problems may be encountered when displaying 16 bit (or above) graphics on Linux machines from programmes rumming on a Sun workstation. A grey scale image will take colours which are typically green and purple. This is due to incompatabilities between integer number represenatation on these machines which is not correctly handled by the X server. When this happens the problem can be fixed by toglling the **endian** button. This re-orders the bytes passed to the graphics server so that all colours and grey scales are rendered as originally intended.

3.4 Graphic Hard Copy Output

The *dump tool* (generated by the **dump** button) provides a means of writing a postscript (or tiff 6.0) image of the current Tv window to a file using the obvious parameter string menus. The extensions ".eps" and ".tiff" are automatically appended.

3.5 Making Movies

The *View Tool* contains a general purpose facility to make a stored copy of any Tv panel and add it ot a list. This list can then be replayed with a fixed display **Time** interval. This is useful for generating timed sequences for psychophysics or functional magnetic resonance experiments, but is also useful for viewing the results of temporal or 3D data analysis. The system has two free parameters, **Time** in hundredths of a second and **Count** which specifies the number of times to loop backwards and forwards through the stored image list if positive, or only in the forward direction if negaive.

3D geometry can be viewed as a rotated movie sequence of *step* images using the rotation axis specified by **Rot x,y**. This functionality is supported by the **make seq** and **show** buttons.

Chapter 4

Macro Tool

4.1 Introduction

This macro facility is potentially very powerful, as it allows the interactive construction and storage of complex vision algorithms making full use of the flexibility of the Tina environment and algorithmic tools. These were written to support and evaluate a range of algorithm variants which can be configured and replayed using the macro facility. Macros avoid the need to write software for specific sequences of algorithm during the process of evaluation and provide a compact decription of exactly what was done in order to process data. This allows key analyses to be repeated at a later date, subject to modification of software or the need for additional results. It provides a natural way for the programmer to divide software intended for the libraries (and therefore provided on buttons) with experiment specific configurations (which can be in a macro).

Macros are particularly useful in conjuction with the *Seqence Tool*, as they allow functions written for operation on a single image to be applied to a sequence of images (see **loop** below). Macro's are editable using a simple text editor and can even be constructed from combinations of other macros.

The generated macro files are in the same, human readable, format as the tinatool replay facility and stored with the extension ".cls" which can be edited directly if neccesary (the syntax used is immediately obvious). The macro facility can thus be regarded as an interactive language which permits the simple exchange of ideas across different computer platforms (via email if necessary) without the need for recompilation of the standard tool.

The macro syntax is deliberately simplistic. For example, it does not support logical functions, as all good algorithms are expected to be based on soft statistical decisions already built into the algorithm (see Programmers Guide). In addition, complex data interactions (such as zoom functions within a Tv) are not stored. Macros are strictly intended for configuation of the tool, variable initialisation and button presses.

The text file identified for execution is provided by the **Macro File** string variable. Unfortunately, the use of absolute screen co-ordinates for the represenation of user-interface position means that macros are not (and never could be) directly portable between machines running non-identical window managers or screens. This includes differences due to font sizes. Although such macros will execute (and the software should not crash), problems may be found with the sizing of windows and button placement. Therefore, if a macro is intended for demonstration purposes it is generally best to construct the macro on the machine which is intended to be used on the day.

4.2 Button Functionality

- **append**

  The specifed **Macro File** is opened and susequent button presses are appended to it. **append** button presses are not executed within macros, thus preventing infinite loops. The button press

is however always noted in the macro file, thus providing a way of identifying new additions to existing macros.

- **close**

  The specifed **Macro File** is closed, with final positions of all widgets and graphics panels stored at the end of the file.

- **run**

  The specifed **Macro File** is executed, repeating variable changes, button presses and tool repositioning as necessary.

- **start**

  The specifed **Macro File** is opened and the first line executed and displayed in the **line**$->$ string.

- **next**

  Exectute the next line of the specified macro.

- **execute**

  Continue macro execution until the end of the file.

- **loop**

  Loop the currect macro execution starting at **start** and ending on **end**. The current value of the loop variable is shown in **Current**.

Chapter 5

Histogram Tool

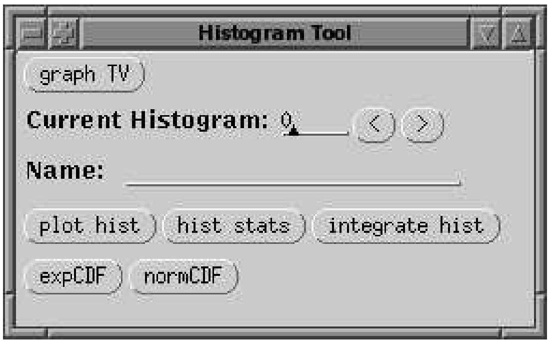

Figure 5.1: Histogram Tool

5.1 Introduction

The histogram tool is designed to allow the program developer to plot and display data distributions anywhere within the software, without the need to have to write additional display software. Histograms are generated and filled using the "hbook" and "hfill" library functions (see Programmers Guilde).

The current histogram for display is manipulated by the < and > buttons. Each will find the next available histogram in the specified direction.

factor Generate a Tv for dsplay.

plot hist Display the current histogram if 1D. If a 2D scatter plot, form an image for display in the **Imcalc Tool**.

hist stats

Print histogram information to the **tinaTool** dialog box.

integrate hist

Replace the current histogram with its integral (from the left).

exp CDF

Plot the histogram along with the cumulative distribution function for an exponential distribution.

norm CDF

Plot the histogram along with the cumulative distribution function for a Gaussian distribution.

Chapter 6

Terrain Tool

6.1 Introduction

The *Terrain Tool* is designed for the visualisation of images which are to be interpretted as depth surfaces. Displayed data is shown in the associated *terrain* Tv, which must be generated and installed. The height of the displayed points is derived from the pixel values.

6.2 Display Options

The tool has a number of switches to allow the user to define the manner in which these surfaces are rendered and displayed. These include;

```
Visible Surface  : Top,  (Bot,all off, all on)
Display          : (lines off,lines on) (fill off, fill on)
Line colour      : black, blue, white, red
Fill Type        : image, shade, mean
```

6.3 User Interaction

The displayed surface can be viewed in the usual manner for 3D data under Tina. That is the zoom facility is set according to;

```
left drag   : up/down/left/right = image shift.

middle drag : left/right = zoom in/out (from centre)
              up/down = rotate display about view direction.

right down  : up/down = 3D rotation about vertical display axis.
```

The effects of these options on the display process are best understood from personal experience.

6.4 Functionality

- **stack pop.** Will get an image from the top of the Tina stack.
- **stack push.** Will push the displayed image back onto the Tina stack.

- **Grimson.** Will fill in the zero's of a sparse image assuming differential continuity and minimum elastic surface energy.
- **surface.** Will generate a projection of the current image surface in the *terrain* Tv according to the parameters in **Terrain Params**, which are;

  `X interval`

  `Y interval`

  `Z scale`

  `Grimson iterations`

  It is advised that the X and Y parameters as set large initially until a good projection direction has been selected.

Chapter 7

Mono, Stereo and Sequence Tools

7.1 Introduction

The *Stack* tool provides a raw data reader. *Stereo* and *Mono* tools perform generic file handling and display for stereo and mono processing respectively. The *Sequence* tool also provides readers for medical image data sets. Files can be flexibly converted directly or by interchanging data via the stack. See appendix for file formats.

7.2 File handling

The simplest image handler is the *Raw Input Tool* which provides direct acces to the data stack within Tina. It allows the user to specify all of the parameters necessary to locate and read a raw binary (or ascii) data block within a file. The parameters of this tool should be quite self explanatory. Typical use involves a trial and error process to select the best parameters while viewing the resulting data in the **Imcalc Tool**'s Tv. This should be the method of last resort for loading proprietary data sets. The other data input tools support formats which contain useful supplemental header information. The *Sequence Tool* in particular supports common medical volume data sets. Once an data set has been loaded it is possible to write out to any of the supported formats, thus achieving image conversion. This process can be batched using macro replays (see below) if many files are involved.

The *Mono Tool* handles the following file types;

Option	Extension	Description
AIFF	.aiff	Image (new Aivru image file format)
PGM	.pgm	portable greylevel map
RAD	.rad	RADIAL image format (Manchester Univ.)
EDGE	.edges	Edge data (binary)
POLY	.poly2D	Geometry
CAM	.cam	Camera geometry
WISP	.wisp	Edge String data
RAS	.ras	SUN rasterfile
DICOM		GIF image file format

An image may have associated files with some or all of these extensions. PGM file reading is supported only for compatability with standard data formats. Generally, image processing requiring co-ordinate mapping or pixel casting will produce images which cannot be supported within an integer format. The AIFF format exists specifically to overcome these kind of deficiencies and its use is recommended for

processed image storage. In order to support sequence processing the filename can be specified including numeric wildcards "#". These are substitued for the numeric **Frame Number** which can be manipulated via the forward and backward iterators > and <. This facility is of primary value when constructing sequence replays within the *Sequence Tool*.

A **scan** facility is provided to provide a listing of files which are compatable with the current specification of the file name. To list a directory contents the directory path should be terminated with a "/". If the file path is unique it will be completed for you in the interface thus avoiding typing errors in locating data.

Stereo tool handles the following file types.

Option	Extension	Description
AIFF	.l.aiff & .r.aiff	Image (new Aivru image file format)
ROI	.roi	stored Tv regions.
EDGE	.l.edges & .r.edges	Edge data (binary)
POLY	.l.poly & .r.poly	2D geometry
LH_G	.poly	3D geometry (old right hand coords)
RH_G	.poly	3D geometry (new right hand coords)
CAM	.l.cam & .r.cam	Camera geometry

An image may have associated files with some or all of these extensions (.l means left images and .r means right image).

The default image pathname is given by the environment variable TINA_IMAGE_DEFAULT, if set otherwise it takes a #defined value.

Again (as in the *Mono Tool*) the filename can be specified including numeric wildcards "#". These are substitued for the numeric **Frame Number** which can be manipulated via the forward and backward iterators > and <. This facility is of primary value when constructing sequence replays within the *Sequence Tool*.

- **Input** input selected file(s) eg house.l.aiff and house.r.aiff (in stereo_tool)

  A camera file is input whenever a .aiff (or .iff) or raw image is input. When inputting a camera file, a heirarchy of default filenames is used. eg. for the image *HOME/images/house/house.aiff*, Tina looks for a camera file in the order:

  ```
  $HOME/images/house/house.cam     (<basename>.cam)
  $HOME/images/house/default.cam   (deafult.cam in same directory)
  $HOME/images/default.cam         (TINA\_CAMERA\_DEFAULT variable)
  $HOME/images/default.cam         (pathname #define'd)
  ```

- **Output** output selected file(s)

  On output a camera file is only written when CAM is selected. (ie. **NOT** when an image is output) and it is written only to the named file (basename.cam) is possible.

7.3 Display

The *Mono Tool* handles a single Tv display device, called *mono*. The *Stereo Tool* has three Tv's associated with it, called *left, right* (image display) and *threed* (3D tina primatives recovered from stereo). The *Sequence Tool* has a single Tv which shows one image from a set of images at any time.

The available display facilities are listed below, you can have any, all or none of them, initially only the image is set to be shown. To change the displayed image press the redraw and repaint buttons on the associated Tv tool.

Mono

```
image   Display current mono image (the one input by the mono_tool).
edges   Display current mono edges. These are drawn as pixels (a box
        around the perimeter of the pixel) in the location of the image
        array where the edge is located (stored).
corner  Display current corners (hard to see at present).
strings Display current mono linked edge strings. These are drawn
        between the current sub-pixel edge locations (in ``salmon'').
geom    Display current mono 2D geometry (conics in ``yellow''and
        poly lines in ``baby blue'' ).
```

Left and Right

```
image    Display current left and right images.
edges    Display current left and right edges.
corner   Display current corners.
str      Display the current left and right linked edge strings.
geom     Display the current left and right 2D geometry.
other    Allows other modules to register alternative image features.
```

Threed

```
geom     Displays 3D geometry.
ground   Displays elected ground plane (if available).
other    Allows other modules to register alternative 3D features.
```

At any stage the left and right images can be used to generate a red green anaglyph which will be displayed in the **Current Tv** using the selected colour-map.

7.4 Mouse Activity

The mouse button on a tv_tool sets the mouse buttons.(The mouse is also set when the mouse button is pressed in another tool). There are three options ;-

pos

```
left down       ipos: the Tv image pixel (integer) position.
middle down     pos2D: the physical image position (sub pixel) with
                respect to image coordinates (takes account of scale).
right down      pos3D: the image position of the underlying physical image
                with respect to 3D co-ordinates of the camera located at
                the centre of the image.
```

grey

```
left down       grey: the grey level of the physical image.

left down       grey: the pixel value of the tv image.
```

All diagnostics are written to the main tool.

7.5 Sequences

The manipulation and analysis of image sequences is handled by the *Sequence Tool.* The file i/o facilities are consistent with the *mono* and *stereo* tool functionality, but now a range of images specified by the parameters **Start** and **End** in the file **Image File** is loaded into a list of images. These sequences include medical image data types such as ACR-nema (NEMA), DICOM and Analyze (ANLZ). This sequence can be viewed in the *sequence* Tv and maipulated using the **first Last** and **jumpto** buttons as well as the forward and backward iterators. The **Del**, **Ins** and **Rep** buttons manipulate this list and the system stack (as displayed in the image calculator).

Image processing can be repeatedly applied to individual images displayed in the *Sequence Tool* via the use of the *Macro Tool.*

7.6 Push and Pop

Tina supports a single data stack onto which a copy of an image can be pushed and from which an image can be popped. In this way images can be shared between the various tools in the Tina system eg. an image can be input into the mono_tool, pushed onto the stack and processed with the *Imcalc Tool.* This avoids every tool requiring specific image i/o code in order to interact with the rest of the system.

Chapter 8

Imcalc Tool

8.1 Introduction

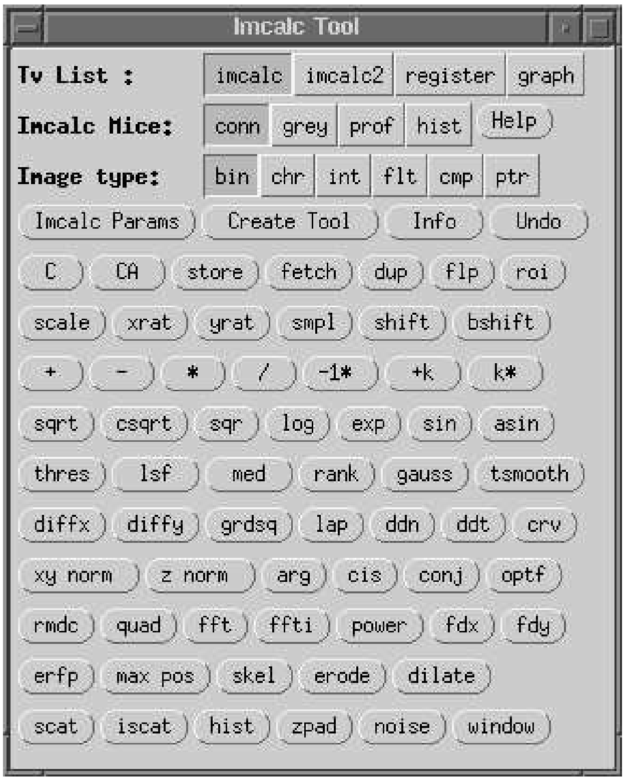

Figure 8.1: Imcalc Tool

The image calculator **Imcalc Tool** is designed to be used as a rapid general purpose image processing algorithm development tool. The interface duplicates the functionality of a common calculator but with data manipulations applied to images rather than single values. It is based around the data processing concept called a **stack**. In this case this is the central data storage and retieval mechanism for exchanging data between the various tools within TINA, which is a last-in, first-out list of data. Most tools within TINA have the facility to **push** and **pop** data on and off the stack, and therefore also pass data in and out of the image calculator. Image calculator processes always operate on the top of the stack (TOS) and sometimes also the next on stack (NOS) data. The images involved are always deleted and results are placed back on the TOS. This mechanism prevents the uncontrolled growth of the stack but requires explicit duplication of any data which is needed again for later stages of processing. Any computational

algorithm can be constructed this way but it is sometimes found quite awkward as it requires good foresight. For this reason an extra data register is provided for storage of intermediate results.

The tool's main use is for algorithm evaluation but it also has uses for general purpose data manipulation, pre-processing and display. This functionality is extended when used in conjunction with the *macro* facility available within TINA which makes it possible to interactively develop and reuse complex image processing algorithms. Though image processing texts contain a multitude of algorithms, algorithms are only included here which have demonstrated useful capabilities during the course of our research. In particular functions have been selected on the basis of providing components in computationally stable algorithms. Such stability can be assessed either by using the technique of error propagation or by Monte-Carlo. Algorithm stability can generally be ensured by demanding that intermediate images have the property of uniform random errors. This tool supports the generation of noise and simple test images in order to perform Monte-Carlo testing. It can also perform noise estimation and the use of scatter plots allows the investigation of data correlation.

The tool has 4 *Tv* tools associated with it *imcalc*, *imcalc2*, *register*, and *graph* for stack and memory displays and graphical output such as profiles and histograms. The required subset of these should be installed before image processing.

The calculator supports several image types; unsigned char, integer, floating point, double precision and complex. The functions decribed below will cast image types according to the minimum requirment for numerical stability but users must bear in mind their required data storage format and its limitations on file output (eg: quantization and truncation). Explicit casting between image types can also be achieved by use of the image type selection menu. This menu displays the resulting image type after all data manipulations and image creation processes. Selecting the appropriate image type will force the image to that storage representation. False binary (actually stored as unsigned char) and pointer types are also indicated. Casting to a type ptr (pointer) will define feature data structures for all pixels with data values greater than IP(thresh) and less than no more than IP(connect) of its neighbours. These structures can then be passed to other tools for continued processing. This is useful for testing out new feature detection algorithms. Casting to "int" will force a 16 bit representation, though internal calculations resulting in "int" will be 32 bit.

At any time the variables defining the current image can be displayed via the *Image Info* button.

The most recent stack manipulation can be reversed by use of the *Undo* facility.

A range of simulated images can be generated in the **Create Tool** sub tool for purposes of algorithmic evaluation (see below).

8.2 Mouse Interaction

After installing the *graph Tv* a range of graph plotting facilities are available for data displayed in the *imcalc Tv.* Selecting *Imcalc Mice* (conn) produces the following functions.

```
left drag    :  Add all all pixels which have 4 way connectivity
                with the identified area to the list of selected
                pixels.


middle drag  :  Construct a connectivity break by setting traversed
                pixels to zero.

right click  :  Construct a complex image mask of selected regions and
                original data by XOR of the the list of selected pixels
                with current mask (if present).

The most recent selection and edit can be undo using the ``undo" facility.
```

Selecting *Imcalc Mice* (prof) produces the following functions.

```
right drag    :   Rubber band straight line. Grey levels along
                 selected along trajectory displayed on associated
                 graph Tv on release.

middle drag  :   Rubber band circle Grey levels along circular
                 boundary displayed on release.
```

Selecting *Imcalc Mice* (hist) produces the following functions.

```
right drag    :   Rubber band straight line. Grey levels along
                 selected along trajectory histogrammed on release.

middle drag  :   Rubber band circle Grey levels within circular
                 boundary histogrammed on release.

left drag   :   Rubber band region of interest. Contents
                 histogrammed on release.
```

Grey level values can be displayed in the main dialog window using the *Imcalc Mice* (grey) selection.

8.3 Stack manipulation.

The following routines are used to directly modify the order of the data stored in the global Tina stack data structure. These are sufficient to allow the construction of any mathematical expression and therefore any image processing algorithm.

- **C** - remove top image (TOS) from stack.
- **CA** - remove all images from stack.
- **store** - save TOS in the storage register. Additional storage can be obtained via **push** and **pop** facilities in many of the other TINA sub-tools.
- **fetch** - retrieve image from the storage register.
- **dup** - duplicate TOS.
- **flp** - flip TOS with next image on stack (NOS).
- **roi** - edit TOS to selected roi. If a polygonal region has been selected in any Tv window within TINA this will be used to define the region of data. Values external to this polygon will be set to zero. If the polygonal region is selected within the **imcalc** Tv the maximum and minimum etent of the polygon vertically and horizontally will be used to define the new image dimensions. Full image dimensions can be restored by "init"ing the **imcalc** Tv before using **roi**.

8.4 Basic Image Manipulation.

- **scale** - change normalisation of TOS to 0-IP(scale) default 255.0 Intended for re-scaling greylevel values to avoid truncation before forcing a particular image storage representation (eg: char) using the type selection bar.
- **yrat** - change vertical aspect ratio of TOS by IP(const) using linear interpolation. Intended for image decimation (ie: const ¡ 1.0). A value of const = 0.5 corresponds to simple averaging. Data values at origional lattice sites are unaffected.

- **xrat** - change horizontal aspect ratio of TOS by IP(const) using linear interpolation. Other comments as **yrat**.
- **smpl** - resample TOS by factor IP(const) using quadratic interpolation. A quadratic 3x3 patch is used to determine the best fit parameters to a 2D quadratic approximation to the local image data when computing sub-pixel locations. Data values at origional lattice sites are unaffected. Intended for image expansion (ie: IP(const) ¿ 1.0).
- **shift** - shift the co-ordinates of TOS by IP(sx,sy). Intended for manipulating images which require specific co-ordinate relationships (eg: stereo images).
- **bshift** - barrel shift TOS by IP(sx,sy). Intended for **fft** based algorithms which may produce unwanted barrel shifts which need to be corrected.

8.5 Basic Algebra

- **+** - add TOS to NOS.
- **-** - subtract NOS from TOS.
- ***** - multiply TOS and NOS.
- **/** - divide TOS by NOS minimum division value limited to IP(const) in order to avoid numerical instability.
- **-1*** - negate TOS.
- **+k** - add IP(const) to TOS.
- **k*** - multiply TOS by IP(const).

8.6 Transendental Algebra.

These functions are useful not only for basic calculation but also for applying non-linear functions to data sets in order to achieve random uniform errors.

- **sqrt** - take square-root of TOS image with sign preserved. Applied to probalitiy or "frequency" image (ie: one with Poisson characteristics) this will result in an image which has uniform random noise (Tina memo 2001-001).
- **csqrt** - complex square-root of TOS.
- **sqr** - take square of TOS image.
- **log** - take logarithm of TOS. Applied to an image which has errors proportional to data value this will result in an image which has uniform random noise.
- **exp** - exponentiate TOS.
- **sin** - take trigonometric sine of TOS.
- **asin** - take trigonometric inverse sine of TOS. Applied to an image with binomial error distribution this will result in an image with uniform random noise (Tina memo 2002-007).

8.7 Standard Noise and Texture Filters.

- **thres** - threshold TOS at making all pixels less than IP(thres) zero, operates only on real component of complex images.
- **lsf** - linear sequential (exponential) smoothing filter of TOS with decay parameter and up/down left right handedness given by IP(sigma,LR,UD). Useful for fast large scale spatial smoothing. Two applications give a reasonable approximation to a Gaussian (ie: Central Limit Theorem).

- **med** - median filter of TOS for 3x3 pixel patch. An edge preserving noise filter which is particularly effective at removing salt and pepper (drop-out) noise. Noise is reduced by a (typical) factor of two and image quantization is unaffected.
- **rank** - rank order filtering of TOS with pixel region IP(range) and expected grey level noise level of IP(const). Useful for allowing parametric statistical measures to be applied to non-parametric image data. The resulting image has approximately bi-nomial noise characteristics which can be transformed to a domain of random uniform errors via the asin mapping.
- **gauss** - gaussian filter of TOS image with standard deviation and convolution area of IP(sigma, range). An optimally compact (spatial/frequency) noise filter. Generally used for band-pass filtering and to reduce noise (particularly the effects of data quantization) before derivative estimation. Errors are generally reduced by an (approximate) factor of two.
- **tsmooth** - local smoothed average of TOS image along direction of minimum gradient. An edge preserving noise filter which reduces image noise by a factor of (approximately) $\sqrt{2}$ (see Tina memo 2003-007).

8.8 Numerical differential functions.

- **diffx** - numerical aproximation to first derivative of TOS in x using (-1,0,1) filter.
- **diffy** - numerical approximation to first derivative of TOS in y.
- **grdsq** - sum squared of diffx and diffy of TOS image.
- **lap** - sum of the second derivatives in x and y of TOS image.
- **ddn** - second order image derivative invariant of TOS image.
- **ddt** - second order image derivative invariant of TOS image.
- **crv** - curvature of TOS image with IP(Precision).

8.9 Complex Image manipulation

- **xy norm** - compute the general image trend (NMR field in-homogenaety) based on the expected level of image noise IP(const). The technique is based on a re-integration of a smoothed local derivative which excludes edges (Tina memo 2000-004).
- **z norm** - compute the most frequent ratio between the TOS and NOS returning the ratio in IP(const).
- **arg** - compute phase of complex TOS image.
- **conj** - complex conjugate of complex TOS image.
- **optf** - construct optimal inverse complex filter for deconvolution from TOS image with noise level set by IP(const).

8.10 Fourier and related Functions.

- **rmdc** - remove dc component of the TOS image. Useful when wishing to display the results of a fft.
- **quad** - create a double sized image from TOS with 4 fold symmetry. This allows fft calculations to be used to give the equivalent DCT manipulations which eliminate boundary effects in processes such as deconvolution.
- **fft** - compute fast fourier transform of TOS image.

- **ffti** - compute inverse fast fourier transform of TOS image.
- **power** - compute power spectrum of TOS fft image.
- **fgradx** - compute x derivative in the fourier domain of complex TOS image. This method for derivative estimation can sometimes give more accurate estimates than spatial filtering, particularly if a smoothing step is not appropriate.
- **fgrady** - compute y derivative in the fourier domain of complex TOS image. Other comments as above.

8.11 Misc.

- **max pos** - indicate position of maximum of TOS image.
- **skel** - perform a binary skeletonisation on the current binary image. Useful for thinning feature based representations of data.
- **erode** - morphological erosion of TOS image with sphere radius IP(range).
- **dilate** - morphological dilation of TOS image with sphere radius IP(range).
- **scat** - generate a 2D scattergram from a complex image with axes given in the range 0-256 for the selected region of interest. The output image has approximate Poisson statistical characteristics.
- **iscat** - generate a float image of probabilities from the selected region in the scattergram. The main use of this process is in the segmentation of "complex" image data.
- **hist** - compute an image of greylevel frequency based on the histogram for the data given within the interval IP(thres) +- IP(range) and return the mean in IP(thresh). The output image has approximate Poisson statistical characteristics. The main use of this process is in the automatic calculation of thresholds for segmentation. Repeated application of the algorithm will set IP(thres) to the mean of the windowed distribution allowing a binary separation of the data on application of a thresholding process.
- **zpad** - extend the current dimensions of the image with a zero boundary by and amount IP(range).
- **noise** - a robust extimate of the local image noise characteristics based on the width of the vertical and horizontalsecond derivative histograms around zero. Intermedate calculations are displayed in the graph Tv and returned data appears in IP(const). Notice of any vertical and horiozontal non-uniformity is given in the main tinatool display.
- **window** - force the maximum and minimum values of the data to be limited to IP(thres) and IP(thres+const) respectively. Use should generally be restricted to display purposes only.

8.12 IP defaults:

```
sx = 0
sy = 0
lr = 0
ud = 0
sigma = 1.0
const = 1.0
range = 5.0
scale = 256.0
thresh = 128.0
connect = 2
```

At any time the current set of parameters can be *store*d to be *restore*d later. This is of most value when constructing **macro** replays (see the *Sequence Tool* for details.)

8.13 Create Tool

The *Create Tool* is a general purpose image creation tool, which can be called from the image calculator sub-tool, for the generation of simulated data of various types. It has the following functional capabiltites.

- **rect** - create rectangle CP(width, height, cx, cy, ax, ax).
- **chequer** - create chequer board CP(width, height, ax, ay). Useful for evaluating edge and corner detectors and for masking geometric regions.
- **noise** - create noise image CP(width, height, ax, ay, noise). The generated image has Gaussian uniform random noise and is intended for Monte-Carlo algorithm evaluations.
- **rds** - random dot stereogram noisy stereo image CP(width, height, ax, ay, ex, ey, noise).
- **delta** - generate a delta function CP(width,height,cx,cy).
- **ellipse** - generate an ellipse to subpixel accuracy CP(width, height, cx, cy, ax, ay).
- **elipsiod** - generate an ellipsoid to subpixel accuracy CP(width, height, cx, cy, ax, ay).
- **sellipse** - generate a super ellipse to subpixel accuracy CP(width, height, cx, cy, ax, ay, ex, ey).
- **shade** - compute shaded image from 3D surface TOS image CP(slant,tilt) using simple Lambertian reflection assumptions.
- **fgabor** - construct gabor filter in fourier domain with central frequency, bandwidth and orientation given by CP(gb_k, gb_b, theta).
- **fshade** - constuct fourier transform of approximate shading convolution funtion CP(slant,tilt) which matches TOS roi, allows subsequent Shape-from-shading using a variation on the technique suggested by Pentland.

In addition simple asci matricies can be read from file and converted into images using the *input output* facility via selection of the appropriate filename parameters.

8.14 CP defaults:

```
width = 128
height = 128
cx = 64.0
ax = 32.0
ex = 64.0
cy = 64.0
ay = 32.0
ey = 64.0
noise = 0.0
slant = 0.785398 (45 degrees)
tilt = 0.785398 (45 degrees)
gb k = 16.0
gb b = 1.0
theta = 0.0
```

8.15 Standard Usage

The *Imcalc Tool* is a stack based image processor which allows the user to visualise the data and coordinate a set of image manipulations which compute the desired output image, in the same way that one would enter a formula on a calculator.

```
Example:
--------
Fourier deconvolution of gaussian smoothed image.

Imcalc Tool quad
Imcalc Tool fft
Image Create delta (equivalent sized image)
Imcalc Tool gauss (sigma=1.0, range > 5.0) (or other deconvolution kernel)
Imcalc Tool fft
Imcalc Tool flp
Imcalc Tool / with IP(const = 128.0)
Imcalc Tool ffti
Imcalc Tool roi (select relavant quadrant)
```

8.16 Non-Parametric Image Subtraction

The non-parametric image subtraction algorithm described in TINA Memo no 2002-004 is implemented within the Imcalc tool as the dscat button. The algorithm subtracts two images by constructing a joint histogram, normalising it to produce a probability histogram and then, for each pair of corresponding pixels from the original image pair, performing an integration along the column of the histogram defined by the pixel grey-level value from the top-of-stack image, summing all values smaller than that at the position defined by the pixel pair. This is directly equivalent to constructing a confidence interval to generate the probability that the grey-level pairing of the pixels was not generated by the same distribution as the bulk of the images. The algorithm is therefore ideal for detecting small changes in image pairs, such as enhancement of MS lesions between pre- and post-GdDTPA contrast agent injection.

The major advantage of the technique over simple image subtraction is that the resulting difference image can be interpreted directly as a probability image, with a uniform distribution (i.e. the probabilities are “honest” in the sense that they reflect frequencies of occurrence). In addition, the difference image is not contaminated with consistent differences between the original image pair e.g. overall illumination differences.

The usage of the algorithm is as follows:

- Start a tinaTool: the one supplied in tina-tools as the example2 toolkit is ideal for this. Start the Imcalc tool and one of Mono, Stereo, or Sequence. Start four new tvtools, and assign them to the imcalc, imcalc2, graph and mono, stereo or sequence Tvs.
- Input two images through either the mono, stereo, or (usual for medical images) sequence tool.
- Push both images to the stack using the ”push” button in the tool you used for input.
- Scale both images to a dynamic range of 0-256, using the ”scale” button in the Imcalc tool. If both images are on the stack, you can do this by pressing ”scale”, then ”flp”, then ”scale” again, in Imcalc.
- Produce a complex image by pressing ”cmp” in the ”image type” choice list in the Imcalc tool.
- Produce a scattergram by pressing the ”scat” button in the Imcalc tool.
- Blur the scattergram a little by pressing the ”gauss” button in the Imcalc tool.

  Ideally this should be done with a kernel roughly equal in size to the noise on the data: you can check the noise by pressing the ”noise” button in Imcalc: the result will be displayed in the Imcalc parameters dialog box as the ”const” field: enter the same number in the ”sigma” field, and change ”range” to be roughly five times larger then ”sigma”. Sigma is, of course, the standard deviation of the Gaussian kernel used, and ”range” is the size at which the kernel is truncated. The Imcalc parameters dialog box can be accessed by pressing ”Imcalc Params” in the Imcalc tool.

- Produce the subtraction image by pressing the "dscat" button in the Imcalc tool.

  This will produce the subtraction image in the imcalc Tv. The subtraction image should have a flat histogram: you can check this by pressing the "hist" button in the "Imcalc Mice" choice list, and then drawing a box in the imcalc tv using the left mouse button (click and drag). The result will be displayed in the Imcalc graph Tv.

  The subtraction image is a probability image, produced by a hypothesis test: pixels which are not drawn from the same distribution as the bulk of the pixels in the image pair will have a low value. Such pixels can be extracted using the thresh button in Imcalc. Press the "-1*" button in Imcalc to invert the image, then enter the desired threshold in the "thresh" field of the Imcalc Parameters dialog box (hint: try -0.05 as a starting point, to threshold the image at the 95% confidence limit). Then press "thresh" in the Imcalc tool. This will set all pixels with lower values (i.e. higher probabilities prior to the inversion) to 0.

Chapter 9

Edge Geom Tool

9.1 Introduction

The *Edge Geom Tool* is an interface to those routines in the Tina system for performing edge detection and subsequent pre-processing for stereo and the recovery of 2D scene descriptions (There are significant changes between this tool and the largely equivalent **Edge Tool** which was in version 4.0).

Two processing modes are available, "mono" and "stereo" ; these relate to the mono and stereo management tools discussed in section 2 of the user guide .

Menus for controlling the Pick and Mouse facilities of associated Tv devices are provided. In addition "pop up" dialog boxes allow various default parameter settings to be manipulated.

Edge data and the 2D geometry recovered from it is now always displayed and manipulated (using the mouse) in real image coordinates even when the edges themselves have been rectified into the equivalent camera coordinate frame (recovered using calibration data).

9.2 Standard Usage

The standard sequence of button presses for stereo processing and the recovery of 2D geometry prior to calculation of 3D measurements (using either the **PMF Stereo Tool** or the **Correlation Stereo Tool**) is given below. It is assumed that the images and camera calibration data have already been input using the stereo management tool. *Tv* display is optional (the edge tool can be run without it) and is again the responsibility of the stereo management tool. If the *Tv*'s associated with left and/or right images exist (are currently installed on the *Tv* tools) their regions(s) of interest will be used by the canny edge detector.

For edge based stereo based upon the PMF algorithm, first make sure the **Image Selection** is set to *stereo images.* The processing order is then

```
canny         (edge detection and display)
edge rectify  (transform the edges to parallel camera coordinates)
geom2         (recover 2D geometry from left and right edges and display)
```

The resulting 3D geomery can be displayed and manipulated using the threed Tv found on the stereo management tool.

The aread based stereo algorithm was developed after PMF in orer to incorporate temporal constraints into the matching process and to make the algorithm suitable for hardware implementation. For area based stereo based upon the multi-scale temporal stretch correlation algorithm, first make sure the **Image Selection** is set to *stereo images.* The processing order is then

```
im_rectify    (transform the image to parallel camera coordinates)
```

```
                manually select rectangular regions of the two images for processing
                using the  {\it left} and {\it right} {\it Tv} ROI facility.
canny           (edge detection and display)
geom2           (recover 2D geometry from left and right edges and display)
```

The resulting 3D geomery can once again displayed and manipulated using the threed Tv found on the stereo management tool.

9.3 Edge Geom Tool Buttons

This section describes each button on the edge tool

- **im rank** Pre-process the images to replace each greylevel value with the local rank (within a region typically 11x11 pixels). This process simulates the first order effect of change detection found in the primate retina in a way which produces data with approximately binomial errors. This is sometimes more suitable for input to correlation based stereo matching than raw greylevels.
- **im rectify** Transform both the left and right stereo images to cyclopean co-ordinates. Don't expect the images to still be centered in the *left* and *right Tv*s.
- **canny** Linked canny edges are obtained, to sub-pixel accuracy, for the selected region of interest. The process is two pass (implementing hysteresis); first the edges with an intensity gradient above a low threshold are identified; then linked edge strings that are entirely below an upper threshold are eliminated (the remaining edges are displayed in purple with blue termination points).

  Edge paramaters:

```
Sigma          The parameter of the gaussian convolution profile used
               to smooth the image prior to the gradient calculation.
               It essentially selects the scale of the edge detector.

Precision      The ratio of the smallest stored value of the guassian
               convolution profile with respect to the largest. Lower
               values give increased precision, larger convolution
               masks and increased computation time.

Lower thres    The lower contrast threshold (see above discussion).

Upper thres    The upper contrast threshold (see above discussion).

Length thres   A lower threshold on the lenght of connected edge strings.
               Shorter sstrings are eliminated.
```

- **edge rectify** Use current parallel camera geometry (recovered from the calibration files) to update the position of edge data to be consistent with it. The actual storage location of each image is unaffected. This process must be performed prior to stereo matching and 3D geometrical recovery.
- **edge derect** Reverses the effect of "edge rectify" to return position to the initial coordinate frame.
- **geom2** Performs geometrical approximation procedures over each of the linked edges strings identified in the left image.

  Geom2 Options :

```
2D Geom Fit     poly:   standard polygonal approximation algorithm.
              linear:   recursively identifies the current longest
                        straight section, segments it, and applies
```

```
                        itself to the remaining sections. The actual
                        line fit is done by orthogonal regression.
                        Sections between straight lines found by
                        "linear" are fit by "poly".
               conic:   recursively identifies the current longest
                        conic section, segments it, and applies itself
                        to the remaining sections. The actual conic fit
                        is done using the bias corrected Kalman filter.
                        Sections between conics are fit by linear. All
                        conics are coerced to be ellipses.
        linear/conic:   [default] first applies linear, then emplys a
                        few heuristics to identify groups of neighbouring
                        straight sections that could be conics and applies
                        conic to underlying edge string. Any conics that
                        are found are extended if possible into neighbouring
                        straight lines and/or conic sections (each new
                        section  must be absorbed as a whole). This results
                        in unique descriptions for each edge string as
                        either a straight line or conic section.

2D Options    join:     after initial fit, try to combine conic sections
                        with other conics and/or straight lines that were
                        not necessariliy neighbouring on, or even from,
                        the same underlying edge string.
```

Geom2 Parameters :

```
Low fit thres         Lower threshold or primitive fitting in pixels.
                      Can be sustained for short deviations of edge
                      strings from primitive.

Up fit thres          Upper threshold of primitive fitting in pixels.
                      Can not be violated.
```

9.4 Pick and Mouse Menus

Pick and mouse menus exist for each of *mono, left* and *right* Tvs. These are used to select pick and mouse activity of their respective Tv devices (see mono and stereo tools and other relevant sections of the user guide for details). They provide facilities to select, display and print edge data and geometry. Other tools (e.g. the mono and stereo tools) are also able to change these mouse and/or pick facilities. Selecting pick and mouse facilities sets the appropriate activity on the associated Tv tool is it exists.

The following pick facilities exist for each Tv device.

```
PICK:

MONO        edge: output relevant values of the selected edge.

LEFT        edge: output relevant values of the selected edge.

RIGHT       edge: output relevant values of the selected edge.
```

Mouse facilities are only currently implemented for left and right Tvs.

```
MOUSE:

LEFT
RIGHT         epi: use currently defined parallel camera geometry
                   to compute the pair of epipolar lines (in left
                   and right images) that pass through the current
                   mouse position and draw them over the extent of
                   the left and right images. Uses only the left
                   mouse button and can be dragged (on button release
                   the final epipolar pair is displayed until the next
                   repaint call to the Tv).

             rast: display the left and right image rasters (horizontal
                   image lines) that pass  through the current mouse
                   position on the left and right image Tv tools. Uses
                   only the left mouse button and can be dragged (on
                   button release the final epipolar pair is displayed
                   until the next repaint call to the Tv). Note that
                   zooming/shifting of left and/or right hand Tv does
                   not affect the position of the raster with respect
                   to the underlying image (although it will shift their
                   position with respect to the Tv itself).
```

Chapter 10

Corner Tool

10.1 Introduction

The *Corner Tool* is an interface to the routines in Tina system for performing corner detection and processing including stereo and reconvery of 2D and 3D point based scene descriptions (Tina memo 1993-001). Two processing modes are available mono and stereo; these relate to the *Mono Tool* and *Stereo Tool* data management system described in other sections of this manual. For the most part, image and feature display management are the responsibility of the *Stereo* and *Mono* Tools.

Parameter dialog boxes are provided for altering a number of corner detection and stereo matching parameter settings and thresholds. Pick and Mouse facilities of associated *Tv* devices can be manipulated to select, display and or print corner data, stereo data and epi-polar geometry in exactly the same way as for the *Edge Tool*.

10.2 Standard Useage

The usual sequence of button presses for stereo processing and the recovery of 3D geometry (assuming image data and calibration files have aleady been loaded in the *Stereo Tool*) follows.

First make sure thet the image selector is set to "stereo" images and that a region of interest for left and right images has been selected as required. Then

```
corner
rectify
stereo
geom
```

Temporal matches can be obtained in either mono or stereo modes. For mono mode simply run the corner detection on the selected region in the mono image, load the next image and repeat detection and then press "temporal". Matches are displayed in their detected locations in the two original images on the *Mono Tvtool*. The repetition of the stereo detection and matching process on a temporally similar set of images allows stereo/temporal matching to be performed. No display facilities are yet available for this process.

10.3 Processing Functions

– **corners.** The corner detector implemented here is our own version of the Plessey algorithm developed by Harris and Stevens. This involves the generation of a corner strenth image, followed by maxima detection and peak fitting to obtain the corner locations to sub-pixel accuracy. Although detected corners do not always correspond to intersection vertices identified

by edge fitting they still provide accurate positional information at reproduceable positions in the image from three dimensional objects. Other corner processors may give locations which are more consistent with edge detection processes.

```
sigma          : The parameter of the Gaussian convolution profile
                 used to filter the image prior to corner detection.
                 This effectively sets the minimum scale of detected
                 features.
precision      : The ratio of the smallest stored value of the Gaussian
                 convolution profile.
```

– **rectify.** Use the current stereo camera geometry to compute the rectified locations of each corner. This process must be performed prior to stereo matching and geometry recovery. Rectified locations are added as extra data to the existing structure so that the original location of each corner is preserved. There is thus no need for the equivalent derectification process in the *Edge Tool.*

– **stereo.** A correlation based matching algorithm that matches corner features which are consitent with the current parallel camera geometry. As this method is correlation based there is a fundamental requirement that the local image patch surrounding each corner is similar. Thus corners on extremal boundaries will normally not be sucessfully matched.

```
Stereo Parameters:

lowdisp     : The lower limit of the allowed disparity range in
              fractions of image width from the centre of each
              region of interest.
updisp      : The upper limit of the disparity range.
width       : The epi-polar band width used to define the valid
              matching area (set large for poor camera geometry).
correlation : The minimum cross correlation measure required between
              corners for them to be regarded as candidate matches.
uniqueness  : The minimum difference in correlation values between
              candidate matches before a match is labelled ambiguous.
              This parameter is particularly important in regions of
              many similar features where the likelihood of getting a
              missmatch is high.
```

– **temporal.** As corner features are well located in both directions on the image plane they can be matched between temporal frames with relatively weak assumptions about the relative transformations between images.

```
Temporal Parameters:

height        : Maximum vertical shift allowed between successive
                locations of a corner in the image plane relative to
                the centre of the region of interest.
width         : Maximum horizontal shift.
```

– **geom.** Compute the 3D location of uniquely matched stereo corners and make them available for display in the *threed Tv.*

10.4 Pick and Mouse Facilities

Pick and mouse menus exist for each of the *mono, left, right* and *threed Tv* tools. These provide facilities to select, display and print corner data and geometry. Other tools (eg *mono* and *stereo*) can also modify these facilities.

The following pick facilities exist for each *Tv* device

```
MONO     corner  : output measured parameters of selected corner.
         c match : display selected matched corners.

LEFT     corner  : output measured parameters of selected corner.
         c match : display list of candidate matches for selected corner.

RIGHT    corner  : output measured parameters of selected corner.
         c match : display list of candidate matches for selected corner.

THREED   print   : print measured parameters of selected geometric feature.
         choose  : keep selected geometric features.
         delete  : delete selected geometric features.
```

For the stereo *left* and *right Tv* tools the corner option, when used in conjunction with the middle mouse button, will add the most recently selected pair of selected corners into the stereo corner matching data structure thus facilitating the possibility of manual matching for difficult scenes or scenes without a good initial calibration.

Mouse facilities are implemented for left and right *Tv's*.

```
LEFT

RIGHT    epi     : use currently defined parallel camera geometry to compute
                   the pair of epi-polar lines that pass though the current
                   mouse position and draw them over the extent of both
                   images.  Uses only the left button which can be dragged.
                   Lines displayed on release remain until a redraw.
         rast    : display the left and right image raster at the current
                   mouse location.
```

Chapter 11

Calib Tool

11.1 Introduction

The calibration tool *Calib Tool* supports calibration of a stereo vision system. The tool comprises a calibration grid matcher, a number of calibration techniques and calibration monitoring facilities. In this software image plane errors are minimised iteratively and robustly from an initial estimate of the calibration (Tina memo 2000-009). The technique permits the selection of arbitrary parameters a-priori and can also deal with radial distortion effects. The methods also permit the estimation of calibration parameter covariances for the purposes of optimal combination. The same software can also be used for the more standard tile based calibration techniques, such as Tsai.

Parameter dialog boxes are provided for altering a number of algorithmic parameters. Calibration is carried out with a local version of the camera model which can be copied from and back to the global model used in the stereo vision modules within Tina.

The left and right cameras of a stereo system can now be calibrated using the wire-frame models (Tina memo 2006-007). These models have been extended to encode occluding boundaries (lines between conic cross-sections) and lateral feature shifting. Lateral shifting is important in order to obtain good alignments to features in curved objects lit by arbitrary illumination. Good alignment is essential for statistical testing of feature visibility.

The recommended strategy for wire frame model constrution is a s follows;

- manually generate a wire-frame .poly file, obtain multiple stereo images of object.
- manually align the model to images and calibrate.
- use output data to construct view files.

Automatic alignment by the model matcher will be progressively supported as more views are added. More information is available in the calibration demonstration directory.

11.2 The Camera Model

A non exclusive selection box is provided at the top of the tool to allow camera model parameters that are to be calculated to be defined. It includes:

```
F           : The focal lengths of the cameras.

Cx and Cy : The centre of the camera's imageing plane.

Ax and Ay : The aspect ratios.

dsrt        : radial (or other) distortion parameters.
```

The *params* button provides access to the numerical values of these selected parameters plus the parameters of the between camera stereo transformation. For some calibration tasks it is necessary to fix the stereo parameters and this can be achieved by setting them to zero values (the minimisation routines used do not modify parameters which are set to zero).

11.3 Tina Interaction

- **get calib.** Fetch the current calibration into the calib tool, if one exists.
- **set calib.** Replace the current Tina claibration with the one in the calib tool.

11.4 Input/Output

- **input cam.** Input a set of calibration files referenced by the *Calib Filename* text string (eg calib_file). There are three files that will be searched for and read if present.

  ```
  calib_file.l.cam  :  Full left camera parameter description.
  calib_file.r.cam  :  Full right camera parameter description.
  calib_file.s.cam  :  Stereo transformation and free parameter
                       covariances.
  ```

  In addition the inputted files are duplicated in

  ```
  calib_file_save.l.cam
  calib_file_save.r.cam
  calib_file_save.s.cam
  ```

  in order to prevent accidental loss.
- **output cam.** Output the calibration files specified by the *Calib Filename* text string.
- **store geom.** save a file of matched image coordinates (left and right) and associated 3D data (seven numbers per data point left image x,y, right image x,y, and 3D x,y,z) to the file specified by the *Base Filename* text string (eg calib_grid). The file is appended with ".cor".
- **restore geom.** read a saved data file specified by the *Base Filename* text string. The data is filtered on reading to check for consistency with the current data model. Data points are removed if both cameras have a reprojection error greater that the parameter "accuracy". This facility is mainly used for iteratively removing outliers from the calibration data during the calibration process.

11.5 Pre-Processing

- **init.** Free up all locally stored calibration structures including the 3D geometry structure created by the grid process the current calibration structures and the curent covariance matrix.
- **grid.** The grid matching routine is designed to locate the vertices of our in-house calibration tile, and match them to a set of 3D positions stored in the file specified by the *Base Filename* text string. Edges must first have been detected using the "canny" routine in the *Edge Tool*, it helps to run this process with slightly higher threshold parameters than normal to remove extraneous noise. Vertex matching is achieved by the following algorithm;

  a) The canny strings are approximated by straight sections (as in the *Edge Tool*) according to the "fit thres" parameter.

  b) Co-linear line segments are sought in the image using a perpendicularity threshold specified by "co thresh" (in pixels).

  c) Groups of co-linear lines are joined together to form longer sections if the endpoints are closer than "join thres" (in pixels).

d) Resulting sets of co-linear lines are compared against a set of stored 2D projective invariants for horizontal and vertical line intersections from the known grid model to identify the lines comprising the grid surface. This process is controlled by the "grid thres" parameter (allowed deviation from the reprojected model in pixels).

e) Identified lines are ordered and labelled starting from the top left hand corner of the grid. Intersections of mainly horizontal and mainly vertical lines are then taken to calculate the sub-pixel location of grid verticies in the image. These vertices are displayed. Faliure of the algorithm is signaled by an incorrect number of recovered horizontal and vertical grid lines.

- **corners.** Use matched corners from *Corner Tool* to construct local data structures with which to perform epi-polar calibration (see *EPI min*).
- bf model Running the "model" initialisation in the Calib Tool will test all results from the model matcher and choose the one with the maximum proportion of a set of sample features passing the hypothesis tests (pixDep = 10 see below). Alternatively, a specific match can be selected using "Model No". A previous alignment can be re-instated by loading the appropriate calibration. This transformation is then applied to the left and right cameras.

11.6 Calibration Methods

There are several calibration procedures available. The choice of these will depend on how the calibration data has been obtained and how much a-priori information needs to be taken into account.

- **Tsai.** This routine takes observed image locations and known 3D measuremets and fits a model to the left and right cameras separately using Tsai's calibration method (on grid data only). The technique does not determine image centres or aspect ratio's and does not deal with radial distortion effects. Though the algorithm can often be used to provide a good initial estimate it is neither optimal or robust and model optimisation should be finished using alternative techniques.
- **IP min.** This routine performs direct image plane minimisation of the difference between the observed and predicted locations of selected data. Left and right calibration is performed separately with respect to the specified "Model" camera parameters and the six parameters of camera transformation. The method used is an iterative minimisation technique which starts by choosing test values around an initial estimate according to the parameter "scale init" on the *Calib Params* menu. Convergence is determined by the *Calib Params* "c_test1" and "c_test2". The first parameter tests for convergence of a minimisation step, minimisation is restarted until the overall reduction in the error function is letts than the second parameter. The technique also excludes data which is inconsistent with the current calibration according to the value of the "accuracy" parameter. The technique is thus performing a robust fit which explicitly excludes outliers from the determination of the minimum.
- **IS min.** This routine performs direct image plane minimisation of the difference between the observed and predicted locations of selected data. Unlike the routine described above however, left and right cameras are calibrated simultaneously. This allows previously determined covariance estimates to be used to constrain the new solution in an optimal manner. Parameters are the same as for **IP min**.
- **EPI min.** This routine performs least-squares off epi-polar minimisation. The routine thus determines the left and right camera models which are most consistent with the observed stereo geometry. The absence of 3D information dictates that this method can only provide information on the ratio of the two focal lengths and the relative transformation between the camera co-ordinate systems up to a scale factor and this only whene there is a fair amount of perspective effects in the matched image data. A covariance estimate from a previous calibration must be used to constrain the minimisation process (see **init covar** below) when extra parameters are selected or the data is insufficient to uniquely determine the parameter set.

– **Model min.** This routine projects the features of a wireframe model (loaded in the **Matcher Tool**) from the view direction specified by the current calibration parameters. It then adjusts the parameters to get the maximum likelihood projection. This process is done for separate cameras and does not yet use the stereo camera covariance. It's main purpose is view and feature validation for automated model construction.

11.7 Covariance Computation

Optimal combination of calibration parameters requires the estimation of covariances from the combined effects of previous fits. This process is supported by the following functions.

– **init covar.** This routine removes the current covariance estimate and replaces it with a ”weak” estimate, too small to constrain subsequent fitting except in those circumstances where there is no constraint on the parameters (see **EPI min**). This process is sufficient to constrain the minimisation solution to one which is close to the initial estimate without unduly affecting the quality of the resulting fit residuals.

– **full covar.** This routine calculates the stereo camera inverse covariance matrix for the current set of *Model* paramters around the current estimate. This inverse is then used in all subsequent calibrations until it is either updated or cleared. The integrity of the calculated covariance is checked and the diagonal elements of the product with the inverse is prited in the main tool dialog window. All elements should be close to 1.0 or 0.0. Faliure can sometimes be rectified by modification of the ”condition” parameter. The magnitude of the calculated covariance is scaled by the ”accuracy” parameter which should be set to the expected feature location accuracy (estimated from the residual plots **epi-cerr**). This allows combination of data from different feature sources which may have differing intrinsic accuracies.

– **epi covar.** This routine calculates the stereo camera inverse covariance matrix for the current set of *Model* paramters around the curent estimate but only for contributions to the model constraint from off epi-polar errors. This routine should thus be used to update the covariance matrix only after minimisation with **EPI min**.

11.8 Calibration Errors

At each stage in calibration it is advisable to visualise the residuals on the data in order to determine the adequacy of the set of fitted model parameters in describing the data. Several utilities are provided by ”calib plots” for this purpose.

– **graph tool.** Pop up a new graph *Tv* for current use.

– **rdist.** Graph the radial residula errors in the graph *Tv*. Left camera residuals are in red and right camera residuals are in blue. Any effects due to radial distortion will manifest themselves as a systematic curving of these distributions. With a small amount of experience this plot can be used to visually varify the adequacy of a calibration.

– **epi-err.** Plot a histogram of errors perpendicular to the epi-polars. As this measure is completely independent of 3D model assumptions, this distribution can be used to estimate the mean location accuracy of calibration features.

– **x-err.** Plot a histogram of errors in the x direction (and therefore approximately along the epi-polars).

Additional information regarding statistical distributionc can also be viewed in the **Histogram Tool**.

The additional fucntionality is also provided.

– **3D geom.** Install the current 3D geometry used in the calibration for viewing in the Tina system 3D *Tv* tool.

– **model proj.** Project the current model view and display hypothesis results (see below).

11.9 Grid Calibration

The calibration grid should be place in front of the two cameras so that it is fully visible in both images. These images should then be preprocessed with the Canny operator (*Edge Tool*). The grid should be viewed so that the first element of the grid data file corresponds to the botton left hand vertex in both images. Illumination should not saturate the images as this can result in badly estimated (even systematically shifted) edge locations. The tile should be inclined at approximately 30 degrees to the cameras focal axis for best results (some degree of perspective distortion is necessary for good parameter estimation).

The vertex positions should be located using the "grid" process. Failiure to recover any full line of vertices across the grid signals incorrect termination, though a few missing vertices here or there can be tolerated.

Obtain an initial estimate of the calibration model (eg read in an old file). This should be either an initial guess of all parameters (in which case you can move on directly to "IP min") or a very good estimate of image centres and aspect ratios.

Use the Tsai algorithm to obtain an initial estimate of the remaining parameters.

Image plane minimisation should be attempted with a simple camera model (ie only focal length included from the model parameter choice menu). Note that when calibrating with a grid, image centres are nearly always very badly determined so these should be either switched off or constrained using a covariance matrix. On no occasion should it be necessary to include both the x and y aspect ratios in the model as either one plus the focal length parameter is sufficient to provide a full parametric description of a projective model.

After calibration the residual distributions should be examined. For good data radial distortion residuals should form a narrow band (less than 1 pixel across) down the centre of the plot. If recalibration is necessary this can proceed from the current estimate but may require the inclusion of more model parameters to obtain good results.

When a sufficiently good calibration is achieved the inverse stereo covariance matrix should be computed and written to file. Subsequent recalibration of the same fixed camera system can then be combined with this current estimate using the **IPS min** process. Copy the resulting calibration back to the Tina system for use in the stereo matching process as required.

The facility to use radial distortions has not yet been fully integrated into the stereo matching algorithms in Tina. The effects of this distortion can be visualised by calibrating with and without this degree of freedom in the model.

11.10 Calibrating Arbitrary Stereo Images

The initial stages for this calibration procedure are the same as for grid calibration. However, instead of using the the *Edge Tool* and grid matching calibration data is obtained from matched stereo corners in the *Corner Tool*. Read in an initial calibration estimate as previously. then initialise the full covariance matrix and minimise using **EPI min**. Visualise the residuals using **epi-er**.

11.11 Model Based calibration

Information contained in the "file.ffgnn" files supplements the basic wire frame model "file.poly" by specifying the half-conics, lines and boundaries visible from specific directions. This set of features is referred to as a cliche. Both files must be loaded via the Smm Matcher Tool.

The standard camera calibration data structure contains transformation parameters which specify the location of the optical centre of the camera in the co-ordinate system of the calibration reference. When applied to object model the transformation therefore corresponds to that which will project the model into the image. Model based calibration can be initialised in one of two ways. Firstly the wire-frame object can be manipulated in the Model Tv associated with the wire-frame model matcher until the view approximates the orientation of the object in the left image. Secondly, an

estimate of the transformation is available following a successful model matching with the Model Tool.

The resulting transformations for both cameras can be visualised by setting the Model Parameters, Project option to "all/print" and using the "model proj" function. This will project all features in the absence of specified view cliches. Use of the "cliche" option will project the features from the closest available cliche to the direction to the transformation. Points passing hypothesis tests are coloured green, those failing are blue.

11.11.1 Model Parameters

Feature No; specifies a single feature from the wire-frame model for display, a value of zero selects all features, and negative numbers identify occluding boundaries.

Starting from a close initial transformation, the "Model min" routine will adjust for the best alignment in both images. Debug plots for sample distributions of Likelihoods and hypothesis tests are made available for viewing in the Histogram Tool.

"gradient/variance" options specify the form of edge model used. The former being a step edge and the latter local image variance computed as a difference of Gaussians.

The "edge/orient" choice menu specifies the hypothesis and likelihood terms.

Optimisation is determined by minimising a cost function in three parts which are separately weighted by the; scale edge, scale angle and scale shift parameters. The theoretical value for each is 1. Other factors are:

Shift factor (1.0); base lateral shifting, as specified in ffgnn files, is multiplied by this factor when applied up to a maximum shift of 15 pixels.

pixSep (2.0); mean sample rate in pixels along lines and ellipses.

pixDiv (1); samples per pixel in lateral shift direction.

OrrErr (0.02); Quantitation of the image into pixels restricts orientation accuracy on measured points. This parameter specifies this limiting accuracy.

Hypothesis verification (not aliment) is controlled by;

hyp_limit (0.01); The test for point detection.

% thresh; specifies the proportion of a curve which must be present in order for it to be displayed.

11.11.2 Recommended settings

The default settings are theoretically motivated and generally adequate for most purposes, the parameters are present on the interface to support evaluation. However, localisation and hypothesis testing are distinctly different tasks requiring different parameters.

For calibration alignment, Edges = "variance", Boundary = "on", Model = "edge", Project = "cliche". Others set to default.

For hypothesis testing, Edges = "variance", Boundary = "on", Model = "edge and angle", Project = "all/print", PixSep = 1.0, scale angle = 0.5 for reflecting (chrome) objects. hyp_limit = 0.02, % thresh = 30. Features passing the hypothesis tests will be printed to standard output in a form suitable for direct inclusion into view files.

Chapter 12

PMF Stereo Tool

12.1 Introduction

The *PMF Stereo Tool* provides an interactive facility for performing stereo processing. It is designed to be used in conjunction with the *Edge Geom* and *Stereo* management tools to explore various edge based stereo matching strategies. This tool is an alternative to to the area based *Correlation Stereo Tool*. As a prerequisite the user should perform edge detection and rectification in the *Edge geom Tool*, the *PMF Stereo Tool* can then be used to complete the generation of 3D geometry. The user has the choice of either following the **Default** processing sequence or taking control of the various matching constraints. Manual matching will not be as fast as using the standard routines because these versions of the algorithm are not optimised. In particular use is not made of the "blocked" memory allocation facilities available in Tina (see Programmers Guide).

Essentially there are four stages to stereo matching.

- Form stereo index.
- Build match and match support structures.
- Compute match support scores for each match.
- Select strongest matches.

In addition disparity histogramming can be used to initialise local match ranges from the distribution of all possible matches.

Within stages 3 and 4 it is possible to one of a number of different modules. More than one support module can be used but only a single selection. After selection, alternative matches are no longer available and it is necessary to reset the matches and recompute support if an alternative stereo selection is required.

12.2 Initial Matching

- **index edges.** Form the stereo index, a raster based indexing scheme that forms lists of short edge strings broken at epi-polar tangencies. This ensures that each sting will cross each raster only once in parallel camera coordinates. Edges must be rectified prior to this stage. The stereo matcher will then limit potential matches to those strings which cross corresponding epi-polar rasters. It is possible for the same edge string to be indexed through adjacent rasters of the index if the rectification process produces aliasing of the edge string.
- **disp hist.** Perform an initial coarse scaled disparity histogram (using a subset of edge data), from which a conservative lower disparity range is recovered for each image region. The default otherwise is to use the whole disparity range defined by the extent of both images.

  Histogram options are;

```
Match    all mats  : let all matches contribute to the matching.
         abs or    : let only matches within the allowable range of
                     absolute orientations contribute to the histogram.
         orient    : let only matches within the allowable range of
                     absolute orientation and of the same contrast sign
                     contribute to the histogram.
         or+con    : let only matches within the allowable range of
                     absolute orientation and similar value of signed
                     contrast be included in the histogram.


Parameters :  Low disp (lower limit of fractional image width disparity)
              Up disp  (upper limit)
```

– **set matches.** Form potential matches between short edge strings accessed through the stereo indices accociated with left and right images. Matches are sought over the default disparity range or that recovered by **disp hist**. At most one match is recoverd for each short edge string from the left image with respect to each overall edge string from the right (allowance is made for edge strings that meander across the same raster several times).

  Matching options are;

```
Match    all mats  : construct all matches.
         abs or    : construct only those matches within the allowable
                     range of absolute orientation.
         orient    : construct only matches within the allowable range
                     of absolute orientation and of the same sign of
                     contrast
         or+con    : construct nly matches within the allowable range
                     of absolute orientation and similar value of signed
                     contrast.

Parameters :  Low disp (lower limit of fractional image width disparity)
              Up disp  (upper limit)
              Vert Disp (vertical raster disparity tolerated)
```

12.3 Cost Options

The following cost options are available, but not all of them can be used by the *Stereo Test Tool* at present. They are used to access the previously calculated and stored match support (next section).

```
null          : use default matching strength of match

match         : use current match weight

string        : use current string matching strength

sub str       : use current sub string matching strength

area          : use current area based matching strength
```

Initially the matching weight is set to the default and all other matches are set to zero.

12.4 Match Support

The following buttons compute and allow the exchange of match support. It is possible to use a number of them, and each of them more than once. Each uses the cost options (above) to determine which currently stored support values to accumulate.

- **ordered.** Use dynamic programming to set match strength of each match. Matches not included in the optimal cost path have strength set to zero otherwise they are reset to default matching strength.
- **string tot.** Accumulate support along globally matched edge strings and set matching strength of each included match.
- **figural tot.** Accumulate match support figurally along edge strings in the left image and set string matching strength of each match. Figural support ofr each match is detemined from the combination of support flowing in each direction along the left edge string from near neighbour compatible matches. Where compatibility is determined by disparity and disparity gradient limits.

12.5 Choosing Matches

The following buttons perform match selection. Only one of them can be used as they result in chosen matches is selected and all other potential matches are rejected. Each uses the cost options defined above to determine which currently stored support value to use as the basis of selection.

- **wta.** Use winner take all strategy (WTA) along rasters to select matches. Most strongly supported matches are fist chosen. Inconsistent matches are then eliminated. This continues until all matches are selected.

  Options;

  ```
  WTA     unique : Enforce uniqueness with respect to left and right
                   images. (Inconsistent matches are those which
                   violate uniqueness).
          order  : Enforce ordering constraint between left and right
                   images. (Inconsistent matches are those which
                   violate order).
  ```

- **DP order.** Use dynamic programming to enforce the ordering constraint along the epi-polar raster.
- **string tot.** Select matches on the basis of globally matched edge strings, with respect to each left hand edge string. Each partial edge string in the left image obtains a unique match subject to the condition that the most strongly supported match strings are selected first. This does not inforce order or uniqueness.

12.6 Mouse Activity

Pick and mouse menus exist for *Left* and *Right Tv*'s. These are used to select pick and mouse activities on their respective *Tv* devices.

Pick;

```
None currently available.
```

Mouse;

```
RIGHT    order : allows interactive examination of matches
                 that would be selected on a particular epi-polar
                 raster by the dynamic programming (order preserving)
```

```
selection algorithm. It does not actually select the
matches.  Only the left button is operative, down
and drag displays epi-polars and up overlays selected
matches using colour coding to discriminate matches.
```

12.7 Default stereo matching

- **stereo** A simple and effective stereo algorithm derived from PMF that works reasonably well.
  Stereo Options :

  ```
  Disparity range    uniform:   using whole displarity range everywhere
                                over the image.
                     histogram: [default] perform an initial coarse scaled
                                disparity histogram (using a subset of edge
                                data) from which a conservative lower disparity
                                range is recovered for each image region (again
                                coarsely quantised).
  ```

  Stereo Parameters :

  ```
  Lower disp            The lower limit of the disparity range.

  Upper disp            The upper limit of the disparity range.
  ```

- **init geom3** Delete the current geometry estimates (this facility is provided in order to permit summary of multiple 3D estimates, including those based on corner features).
- **geom3** Combined 2D geometry and stereo matches for the underlying edge strings to obtain 3D line sections. The fitting of disparity to lines is performed in the rectified coordinates. That is edges must be rectified prior to both **stereo** and **geom2** (the actual order of **stereo** and **geom2** is unimportant). The 3D geometry is with respect to the coordinate frame of the left camera of the imaginary camera geometry (it shares its origin with the true optical centre of the real left camera).
  Geom3 parameters :

  ```
  3D fit thres          Threshold on deviation from 3D primitive in
                        disparity space (pixels).
  ```

- **left** Transform 3D geometry from left hand rectified parallel camera frame to true left hand camera frame.

Chapter 13

Correlation Stereo Tool

13.1 Introduction

The *Correlation Stereo Tool* allows the correlation based matching of stereo image pairs. It is a direct substitute for the **PMF Stereo Tool**, based on more extensive development and testing. The algorithm was specifically developed with hardware implementation in mind (Tina memos 1994-002 and 1995-001).

The similarity measure used by the correlator is user selectable as is the correlation algorithm. The available algorithms are all based on finding similarity between small regions or blocks in the left and right images. The image blocks used for correlation can be the original images, or they can be derived from warped blocks of the original images. There are two methods of image warping techniques which can be used: stretch correlation and shear correlation. In addition to the correlation functions, there are various image preprocessing techniques which can be employed prior to correlation, typical image preprocessing for edge based stereo matching might include horizontal gaussian smoothing, and enhancement of non-horizontal edges. The range of preprocessing available covers a very large subset of the techniques investigated in the published literature, allowing a wide range of alternative approaches to be evaluated for specific data sets.

One key feature of this approach is that the results of stereo processing are available to subsequent analysis. This allows the algorithm either to be iterated in order to refine the set of matches found, or to be used in a temporal context, where the results of previous frames are used to constrain the next stereo match. This gives real benefits in the reliability of match results over single frame solutions (Tina memo 2003-009). More information and references to published literature is available in the stereo demonstration directory.

13.2 Standard Usage

A stereo image pair along with the appropriate camera calibration is read into Tina via the *Stereo Tool*. Regions of interest are setup via the *Tvtool* or read in via **Read roi**, these will define the subsequent working area within the image pair. The images are transformed to cyclopean co-ordinates and edges detected (see the **Edge Geom Tool**). The following minimal button sequence must then be used:

- **Preproc.** Perform edge detection and edge enhancement.
- **Correlate.** Perform Stereo Correlation on the preprocessed images and generate a disparity image map which corresponds to the left image.
- **proj disp** Store the results of matching in the 3D output geometry as disparity space measurements, for viewing in the **Threed** Tv.

This process is an alternative to using the default stereo matcher (**stereo**). The resulting matches can be used to determine quantitative 3D measurement using the **geom3** button and associated functions

(see below).

13.3 Stereo Correlation Matching

13.3.1 preproc params.

Preprocessing of the source images to enhance edge information is performed as well as edge detection. The enhancement technique used is determined by the **enhance** choice selection and the parameters set up by the **Cor params** dialogue box. Enhancement can be any of:

```
Dx gauss. Horizontal first differences followed by horizontal gaussian
          smoothing with distribution width = sigma1.

Dx.       Horizontal first differences.

DoG.      Difference of gaussians filtering with  DoG = k1 x G_sigma 1
                                                + k2  G_sigma 2.
grad      Summed squares of vertical and horizontal derivatives (needed for complete 3D data extract

None.     No preprocessing.
```

The **DoG** parameters are used by **preproc**.

The edge detection is performed automatically and the number of edge pixels within the block is used (in the absense of an input disparity image) to determine whether a block contains enough information to attempt correlation.

13.3.2 Correlate.

The images are correlated using the similarity measure specified by **cor_type**, this could be any of **dotprod**, **eucidean** or **mad** (mean absolute distance). The search **range**, block **height** and **width** are set up in the **cor params** dialogue box. In addition to the different similarity measures, the correlation algorithm can be either **shear**, **stretch** or **fast stretch**, the parameters of which can also be set up in the **cor params** dialogue box. The fast stretch algorithm is more efficient than the standard stretch algorithm but gives less flexibility in terms of output and similarity measure, however, for large values of **range** it speeds up the experiment consiserably.

correlate params The following parameters can be accessed via the interactive dialogue box labeled **Cor params**:

Height, **Width**. Dimensions of correlation blocks.

Stretch, **Compress**. Defines the limits of the stretch algorithm. Both must be positive or zero, and **compress** should not be bigger than **width/2**.

Shear. Defines the range of values for the shear algorithm. This number, must be positive.

EDelta. During the creation of the output disparity, a compatibility criterion exists whereby an edge must be present, to within $\pm Edelta$, when the disparity estimate is projected into the right image.

Range. The disparity search range. The search takes place over $initial \pm range$, where initial is supplied by either the input disparity image or is estimated from the regions of interest.

CtF Scaling The stretch correlation algorithm is run initially on a down sampled version of both stereo images, inorder to home in on the solution which is consistent across scales. This choice menu determines the greatest level of down sampling used.

13.3.3 disp drad.

Having obtained a disparity image from typically **Edges** or **Corners** the data can be viewed using **Display 3D**. The 3D projection of the data may contain points which are visibly incorrect. A filter for the incorrect data which uses the disparity gradient limit can be applied to the disparity image. The parameters of **Disp Grad** are accessible via the **Disp Grad Params** dialogue box.

disp grad params

The following parameters, used by **Disp Grad**, can be accessed by this dialogue box:

Limit. The smaller this value is, then the larger the amount of rejected data. Psycho-physics research suggests that a value of 1.0 is used in the human vision system as a limit for stereo fusion, and a value of greater than 2.0 along an epipolar represents order reversal.

Must Pass %. The disparity gradient test is binary with respect to the imposed limit, the percentage of the surrounding points with which the data point in question must pass is defined with this value. In normal scenes a point will not be expected to pass the disparity gradient test with 100

Nearest N. This number defines the N nearest points on which the disparity gradient test will be applied. The search path is a rectangular spiral outwards from the data point in question.

When setting up these parameters a tradeoff must be found between removing all of the incorrect points, which fail the disparity gradient test, and removing to many correct points which also violate the disparity gradient test. Bearing in mind that experimental error is increased both by including incorrect data and by discarding correct and potentially useful data points.

13.4 Default stereo matching

- **stereo** An effective stereo algorithm with default parameters as published.
- **init geom3** Delete the current geometry estimates (this facility is provided in order to permit summary of multiple 3D estimates, including those based on corner features).
- **geom3** Combined 2D geometry and stereo matches for the underlying edge strings to obtain 3D line sections. The fitting of disparity to lines is performed in the rectified coordinates. That is edges must be rectified prior to both **stereo** and **geom2** (the actual order of **stereo** and **geom2** is unimportant). The 3D geometry is with respect to the cyclopean frame of the left camera geometry.

  Geom3 parameters :

  ```
  3D fit thres          Threshold on deviation from 3D primitive in
                        disparity space (pixels).
  ```

- **left** Transform 3D geometry from left hand rectified parallel camera frame to true left hand camera frame.

Chapter 14

Matching Tool

14.1 Introduction

The *MatcherTool* provides 3D model creation and matching facilities. The model matcher also computes the transformation which takes the model into the scene. The model matcher bases its initial matching hypotheses upon partial congruencies identified between scene descriptions and subsets of features chosen from the model. Each such subset (or "group") of model features provides a matching context/cliche. This embodies certain viewpoint dependencies and allows the computational expense of exhaustive search to be avoided. Furthermore, in order to reduce complexity yet further, a single feature in the group, the focus feature, is required to have a good match at all times (Tina memo 2003-006).

Models and their matching contexts (focus features and associated groups) can either be read from files (see Appendix for a definition of the format of these files), generated at run time, or a combination of both (the model read from disc and the allowable matching contexts determined at run time). Once the model has been obtained the process of matching it against a current scene description (assuming it has already been obtained in the usual way using the Edge tool) has 2 stages. The first is to build a pairwise relations table for the model and scene; this contains information about the invariant relationships between pairs of features. The second is to perform a constrained search over the tables of the model and scene to identify cliques of above threshold cardinality between matching features. These are used to compute potential transforms and recruit further matches. The model matcher has specialised *File/View* facilities for reading/writing, and display/manipulation of scene and model data. Using the view facility and associated Tv devices (*model* and*scene*) the matched model can be displayed in the transformed position over the scene description. The matcher has 2 parameter cycles and toggle selectors for adjusting various pair wise relationships table and matching algorithm parameters.

14.2 Standard Usage

Initialise the various parts of the tool, installing the model and scence display Tv's, and read in a set of data (model, scene and cliches). To contiune with the matching process press the following sequence of buttons;

```
build tables  :build the pairwise geometric tables.
smm matcher   :match the geometric tables for the scene to those
               for the model and display consistent cliches.
comp trans    :match remaining scene data to 3D model and display
               the new set of matching features.
trans model   :compute the transformation of the model to the scene
               and display the model over the scene data.
```

14.3 Matcher File/View

The matcher's File/View tool is of a familiar design (see*StereoTool* and *MonoTool*).

Pick The matcher's File/View tool controls a pair of logical*Tv*'s called *model* and *scene* to display model and scene primitives respectively. A host of "pick" facilities are available for each through the *model* and *scene* pick menus. The 3D geometrical descriptions comprising model and scene are displayed with arrow heads indicating directionality. All pick facilities work on the these 3D geometrical descriptions.

Model

null

choose select geometrical primitives and delete remainder.

delete delete selected geometrical primitives.

direct select directedness of chosen primitives (straight lines only) in accordance with the end closest to the pointer.

pairs show the features with which the most recently selected primitive shares pair wise table relations (possibly all). In the current implementation the pick calls a repaint so the display is transient (3 seconds).

matches show the features (in the other*Tv* device) with which the most recently selected feature can match according to the conservative matching criteria used on focus features.

focus select the most recently picked feature to be the current focus feature.

group select the list of picked features to be the feature group associated with the current focus feature. In addition the mouse mode of *model* and *scene* can be used to add matches between features (only straight features at present). Simply pick scene and model descriptions alternately (in any order) using the left hand mouse button (the match is made against the most recent selection made in the other*Tv* device). Note that matches are direction specific and the mouse is able to select the direction of features when constructing matches.

Scene as model (above) but without "focus" & "group".

File Files with the following extensions:

Model .poly

Scene .poly

Cliches Old .ffgnn

Cliches New .ffg

input/output. Reading in a model clears the current cliche list and hence cliches must be read after models.

Directory Name. part of the pathname to use when reading/writing files.

Base Name. part of the pathname to use when reading/writing files.

14.4 Geometry

These buttons get geometry from Tina.

get model. Obtain the current model from the 3D geometry created by (or input to) the stereo and edge tools.

get scene. Obtain the current scene description from the 3D geometry created by (or input to) the stereo and edge tools.

14.5 Initialisation

These buttons can be helpful for initialising the matcher

init matches. Sets the current matchlist to null. Useful if performing interactive matching using the mouse facility.

init cliches. Sets the current cliche list to null. Useful if performing interactive cliche building using the **group** and **focus** pick facilities on the model's Tv tool.

14.6 Interactive Matching

This row of buttons are for interactive use of the model matcher.

add cliche. The current focus and group features (selected by the mouse) are added to the list of current context cliches.

comp trans. Use the current list of matches (obtained interactively and/ or as a result of model matching) to compute a transform (if possible within set tolerance) and apply that transform to obtain a new list of matches that are constant with it. Note that as a new match list is produced (and it is possibly null) then repeated application can give different results which may or may not converge. The computed transform is written to the text sub window of the main Tina tool.

trans model. Displays the object model transformed (according to the currently computed transform if one exists) into the 3D scene. The display is transient in that if the *sceneTvTool* activates a new repaint (as a result of a zoom say) the model will not be redisplayed.

Allowed Trans. option can be used to restrict the allowable transform.

unlimited

limited

14.7 Automatic Matching

The final row of buttons perform automatic matching functions.

build tables. Builds the pairwise relationships table for the current scene and model. The

smm matcher. Performs model matching using the current model and context cliche list against the current scene description. Pairwise relations must be set up prior to matching. If the model matcher is successful then the individual scene and model feature matches included in the best model match are highlighted (matched features are displayed the same colour). The

smm next. Continue to the next best match found by the model matcher.

Pair Wise Rels option is used to determine whether all entries in the table are to be filled for the scene or just the closest n non parallel relations (where n is the number of table entries). All table entries are always filled for the model.

Specific match results can be selected from the reulting list of matches using the 'go to N' facility (where N is the number specified).

Matched results can be evaluated (the best location evaluated and specific feature hypotheses verified) using the functionality available in the *Calib Tool*.

14.8 PWR Params

clique size The number of relationships to add to the pairwise relationships table for each scene feature (due to the reciprocal nature of such relationships the grand total of relations a line ends up involved in may exceed this number).

pos error The magnitude of a positional error threshold assumed for line data in mm.

rot error The magnitude of a rotational error threshold assumed for line data in radians.

Lenh thres Threshold on length of scene feature to include in model matching. Note that the current scene error is only updated by building the pairwise relations table.

14.9 Match Parameters

clique size The threshold cardinality of mutually consistent cliques identified in any one context to be allowed to go on to form hypothetical matches.

Max rot Threshold on allowable rotation (in radians) to use if the *Allowed Trans* toggle is set.

max trans Threshold on the magnitude of allowed translation to use if the *Allowed Trans* toggle is set.

Len ratio The threshold which must be exceeded between matches of focus feature.

Chapter 15

Pairs Tool

15.1 Introduction

The *Pairs Tool* allows the user to explore the use of geometric pairwise histograms for 2D object recognition (Tina memo 1995-004). The software can be used to automatically construct edge based representations of large numbers of objects and then recognise and locate these objects in cluttered scenes. The technique is view based and therefore matches closely the processes thought to exist in the primate brain (Tina memo 2000-002). The tool interacts with linear geometry in the *Mono Tool* (the mono scene). The tool also provides model database facilities. More information and references to the published literature is available in the pairwise demonstration directory.

15.2 Standard Usage

Once *mono* geometry data and an object database have been loaded (see below) the standard sequence of operations for recognition and location is as follows:

```
match lines   : match the stored line histogrames to the scene.
segment model : display the sections of data consistent with
                the chosen Model Name.
locate models : locate all examples of the chosen Model Name.
graph         : display the radial error from the probabilistic hough
                transform.
```

15.3 Scene and Model Data

- **canny** Run the standard canny with default parameters. These parameters can be updated using the edge tool routines if required.
- **Line Geom2** Run the standard 2D geometry generation routines with default paramters for extended lines only.
- **Edgel Geom2** Generate short 2D line geometry for individual pixels.
- **add model** The model file specified by the fields 'directory name' and 'model name' is added to the model database. All model files should be of type .poly and should only comprise linear geometry.
- **clear** The model database is cleared.

15.4 Single Line Functions

The user can pick single lines from the mono scene for further manipulation. To enter 'pick' mode the user must fist select 'geom' from the pairwise tool 'pick:mono' menu and then select 'pick' from the tv tool 'mouse' menu. Lines can then be picked from the mono scene using the left mouse button (picked lines are highlighted). Once the required lines have been picked the selection is confirmed with the middle mouse button.

- **histograms** A pairwise histogram is generated for each picked line. These histograms are placed onto the image stack for viewing with the *Imcalc Tool*.
- **match line** A pairwise histogram is generated for the last picked line. The best matching model histogram is then identified and the respective model is plotted over the model scene. From a single histogram match, the location of the model is constrained to four possible positions so the plotted model may need manipulating into the correct place (see Matched Model Functions below).

15.5 Complete Scene Functions

- **match lines** A histogram is generated for every scene line and then the best set of model histograms which match to each scene histogram are identified (see Match Parameters dialog box).
- **segment scene** All mono scene lines which have matched to the model specified in the field 'model name' are highlighted.
- **locate models** An attempt is made to locate any models which appear in the mono scene. For each model a location hough space is generated. Peaks in this hough space hypothesize the location of an instance of the model. For each peak in this hough space a separate orientation hough space is generated. The maximum peak in this hough space hypothesizes the orientation of the model. Finally located models are plotted over the mono scene *Tv*.

  If a model name is specified in the field 'model name' then any hough spaces associated with this model are placed onto the image stack.

15.6 Matched Model Functions.

A number of functions are available to manipulate and display matched models.

- **redraw** Any matched models - from either the 'match line' function or the 'locate models' function - are displayed in the mono scene.
- **rotate** The matched model from the 'line to model' function is rotated by 180 degrees and redisplayed. This allows the model to be moved between two of its four possible locations. (see 'match line' function above).
- **mirror** The matched model from the 'line to model' function is reflected along the line used in the match and redisplayed. This allows the model to be moved between two of its four possible locations. (see 'match line' function above).

15.7 Dialog Boxes

There are a set of possible pairwise histogram formulations which embody a varying degree of invariance characteristics including the standard: translation, rotation, scale (with stretch correlation, Tina memo 1996-004) together with robustness to line fragmentation and linear curve approximation. These variations are selectable according to the value set in the "pairs type" choice menu.

- **mirror** The original mirror symetric pairwise histograms. Generates a plot with dimensions : 0 - PI, 0 - +dbin_max.

  additional invariances: mirror symetry, line contrast.

- **rotate** Assumes (0 to PI) orientations of reference and object lines and generates a plot with dimensions : 0 - PI, -dbin_max - +dbin_max.

  additional invariances: line contrast.

- **directed** A special case of (rotate). Fixed (arbitrary) reference line orientation with comparison to intersection direction. Generates a plot with dimensions : 0 - 2PI, -dbin_max - +dbin_max.

  additional invariances: line contrast.

- **contrast** Orientation of reference and object line fixed by contrast.Generates a plot with dimensions : 0 - 2PI, -dbin_max - +dbin_max.

  additional invariances: none.

Various other algorithm variations can be set according to the parameters and choice variables defined in the menus *Match Params* (num_best_matches: The number of best matches which are identified during 'match lines'.), *Hough Params* (Parameters used in the construction of hough spaces and identification of peaks.)

Chapter 16

Colour Image Tool

16.1 Introduction

Most machine vision research is performed using grey-scale images. There are three main reasons for this. The first is practical: cheap colour cameras work by using a grid of red, green and blue filters arranged over the detector. Therefore, the RGB components of any given pixel were generated from slightly different spatial positions: this spatial error complicates any subsequent analysis. Cameras exist that contain optics to split incoming light into three separate paths, passing through red, green and blue filters and falling on separate detectors: professional television cameras are one example. However, these cameras tend to be much more expensive (a factor of 400 is typical) and therefore fall outside the budgets of most researchers. The second reason is theoretical: whilst it is simple to define an algebra for grey-scale images (as represented by the Imcalc tool in Tina), it is much more difficult to define a satisfactory equivalent for colour images. The third reason is biological: humans can operate perfectly well in a grey-scale environment, for instance watching a black-and-white movie, implying that colour perception is not essential in order to perform the majority of basic visual tasks. For these reasons, colour-based algorithmic functionality in Tina is limited.

There is one exception to the above: the three colour channels of an image can be treated as independent images, and data fusion performed for processes such as segmentation. Therefore, whilst the infrastructure of Tina currently provides little support for colour images (for example there is no colour Tv tool, or colour image structure) the Tina Colour Image tool provides support for input of colour images as a separate RGB fields, conversions to a number of other colour space, and colour segmentation by non-parametric density estimation in colour space, as described in Tina Memo no. 2001-015.

16.2 Colour Theory 101

Colour representation is a complex subject, so a short preamble on colour theory is provided. This has been highly simplified: for instance, no mention is made of defining the origin (the so-called "white point") of colour spaces. More in-depth explanations are readily available on-line: see for example the colour space FAQ at www.poynton.com.

16.2.1 Colour spaces

The human eye contains three types of colour-sensitive cells, known as cones, located on the retina. They are separately sensitive to red, green and blue light. Therefore, a given pixel in a colour image can be represented as a combination of three values. In simple terms, these specify the amount of red, green and blue light that needs to be emitted by the equivalent pixel on a computer monitor in order to display that colour. The human visual system operates by superposition of the signals from the cones, and so combines the red, green and blue light to give a perception equivalent to the original colour. Note that the actual light emitted by the monitor does not replicate the full spectrum of the original light captured

to produce the image: in order for the human visual system to perceive it as the same colour, it only needs to closely match the original intensities in the red, green and blue parts of the spectrum. Since light of these three colours can be added together to reproduce any colour, they are referred to as the additive primaries.

The additive primaries are only applicable to systems that work by emission of light. Systems that work by the absorption of light, such as oil painting, are based on dyes that absorb a specific part of the spectrum. They use a different set of primary colours: cyan, magenta and yellow, which are used in pairs. For example, in order to reflect red light from a white source of illumination, all but the red light must be absorbed, by combining dyes that absorb both cyan and yellow. The physics of the combination process is more complicated than that for emission-based systems, and strictly speaking is multiplicative, but these primary colours are usually referred to as the subtractive primaries.

We are deal here only with the additive primaries red, blue, and green. The colour of any given pixel is represented by a vector of three values, one for the intensity of each primary colour. The colour can therefore be considered as a point in a three-dimensional space, where the x-axis represents red, the y-axis green, and the z-axis blue. This space is known as the RGB colour space (or RGB colour cube). Clearly, we can then rotate to any set of three non-degenerate axes in the space, producing an alternative representation of colour that still spans the same space of possible colours (known as the gamut of the colour space). Various colour spaces have been designed for different tasks: Tina provides functions to convert images into a number of the more well-known colour spaces.

Considering the RGB colour space, the point (0,0,0) represents black, and the point (1,1,1) on the opposite corner of the cube white. All points on the axis between these two points represent shades of grey. The information encoded along this axis is called the luminance, represents the total intensity of light received from a scene. Grey-scale images of colour scenes are produced by recording only the luminance information. The actual colour (e.g. red, orange yellow etc.) is represented by a vector away from this axis in a plane orthogonal to it, and is called the chrominance. To a first approximation, luminance is dependent on the level of illumination of a scene, whereas chrominance is not. In many colour analysis tasks, such as colour segmentation, it is useful to discard the luminance information in order to remove the effects of varying levels of illumination, retaining only the chrominance information. Therefore, many colour spaces involve a rotation such that one axis of the space lies along the luminance axis. It is worthwhile to note that, whilst discarding the luminance axis of a colour space in order to normalise for illumination differences usually works to some extent, the actual situation is far more complicated: shadows, for instance, represent areas that may be partly illuminated by coloured light reflected from other objects in the scene, changing their chrominance.

The RGB Colour Space

The RGB colour space is commonly used in colour image formats since it directly represents the light that needs to be emitted by a TV or computer monitor in order to reproduce the colour to the satisfaction of a human observer. However, it has the drawback that the luminance information is split across all three axes.

The HSI Colour Space

The HSI colour space (hue, saturation and intensity) attempts to produce a more intuitive representation of colour. The I axis represents the luminance information. The H and S axes are polar coordinates on the plane orthogonal to I. H is the angle, specified such that red is at zero, green at 120 degrees, and blue at 240 degrees. Hue thus represents what humans implicitly understand as colour. S is the magnitude of the colour vector projected in the plane orthogonal to I, and so represents the difference between pastel colours (low saturation) and vibrant colours (high saturation). The main drawback of this colour space is that hue is undefined if saturation is zero, making error propagation in transformations from the RGB colour space more complicated.

It should also be noted that, although the HSI colour space may be more intuitive, is not “perceptual”, in the sense that small displacements of equal size in different parts of the colour space will be perceived by human observers as changes of different magnitude. Attempts have been made to define such colour

spaces: CIE-LAB and CIE-LUV are two examples.

The YIQ and YUV Colour Spaces

The YIQ colour space model is use in U.S. commercial colour television broadcasting (NTSC). It is a rotation of the RGB colour space such that the Y axis contains the luminance information, allowing backwards-compatibility with black-and-white colour Tv's, which display only this axis of the colour space. The chrominance information is contained in the I (orange-blue) and Q (purple-green) axes, which are roughly orthogonal. The reason for this arrangement is that the human visual system is much more sensitive to changes in the I axis than in the Q axis, allowing the Q axis to be transmitted with less fidelity, conserving bandwidth.

The television broadcasting model PAL, used in the UK, most of Europe, and some other places such as Hong Kong, uses a closely related colour space called YUV: the difference between the two is a 33 degree rotation of the chrominance axes.

The exact scaling of the chrominance axes in these colour spaces was defined to fit the emission spectra of the phosphors used in colour Tv screens in various parts of the world. These change over time as technology improves, making these colour spaces subject to change in terms of the conversion to and from RGB. In addition, the axes are not exactly orthogonal. Therefore, in general these colour spaces have no place in digital image processing.

The IJK Colour Space

The above colour spaces have drawbacks when used in colour segmentation: RGB factors the luminance information into all three axes, HSI is a polar representation, so that hue is undefined when saturation is zero, and error propagation is unstable for small saturation values, and YIQ and YUV use more-or-less arbitrary scalings. Therefore, Tina implements a colour space developed in-house specifically for colour segmentation, known as IJK. The I axis is the luminance axis of the RGB colour space. The J axis lies in the plane orthogonal to the I axis, and is in the direction of red in the original RGB colour space. The K axis is orthogonal to both the I and J axes, and so lies in the plane orthogonal to the I axis in the direction of blue-green. The advantages of this colour space are that the luminance information is separated out onto the I axes, all three axes are orthogonal, and the conversion to RGB (and so the propagation of errors from RGB values) is particularly simple.

16.3 The Tina Colour Tool

16.3.1 The Tv List

The Tina Colour Tool has three Tv's: one each for the red, green and blue fields of the currently loaded colour image. These can be installed onto tvtools in the usual way (selecting the Tv you wish to install from the Tv choice list, starting a new tvtool from the top level tinaTool window, and pressing ”install” on the tvtool).

16.3.2 Image Input and Output

Tina supports a single format, portable pixel map (PPM), for colour image input. This format is a member of the PNM family of image formats, which comprises PBM (1bpp monochrome images), PGM (grey-scale images) and PPM (colour images). Each stores image data in either ASCII or raw format. Therefore, the format is particularly simple in terms of writing input or output functions, but egregiously expensive in terms of disk space. Nevertheless, since the inception of the NETPBM package, most image conversion programmes will provide functionality enabling conversion to PNM from other formats (indeed this was one of the purposes of the NETPBM package), and so it should be relatively easy to convert images into this format for use in TINA.

In order to load a colour image into the Tina Colour Image tool, enter the path (either absolute or relative to the directory in which the tinaTool executable is located) into the directory field, and enter the file name into the filename field. Note that the file extension must be entered explicitly. Then press the "Input PPM" button to input the image. The image is split into its red, green and blue colour fields, which are displayed in the relevant Tv's.

The process can be reversed by pressing the "Output PPM" button. This will combine the red, green and blue fields currently loaded into memory into a ppm image, and output it to the directory and filename specified in the relevant fields. Ensure that you change the filename to something other than that of the original image you loaded, as the function will not warn you about overwriting.

16.3.3 Pushing and Popping Images to and from the Stack

The red, green and blue fields of the currently loaded image can be pushed to the stack using the first line of the "Colour space conversion" section of the tool. Select the fields you wish to push to the stack in the RGB check list, and then press the "RGB push" button. The images will be pushed in reverse order, so that for example if all three buttons on the check list are selected, the red field will end up on top of the stack, the green field will be second on the stack, and the blue field third. The images will then be available for manipulation in the Imcalc tool, or display in the Imcalc Tv's.

The process can be reversed using the "R Pop", "G Pop" and "B pop" buttons. Pressing these will take the top image from the Imcalc stack and load it into memory as the currently stored red, green or blue field. If one of the other fields has already been loaded (either by loading a colour image or by popping and image from the stack) then the size of the new field must be the same as that of the others already loaded.

16.3.4 Colour Space Conversions

The Tina colour tool supports conversion to the HSI, YIQ and IJK colour spaces. The results of the conversion will be pushed to the Imcalc stack, in the same way as the "RGB Push" function described above. For each colour space conversion, select the fields you wish to push from the relevant check list, and press the relevant "XXX Push" button. For example, if you wish to convert to the HSI colour space, but only want to push the H and S fields, select H and S on the check list and press the "HSI Push" button. Again, the images are pushed in reverse order so that the H image will end up on top of the stack, and the S image will be second on the stack. Colour space conversions do not affect the colour fields currently stored in memory.

16.3.5 RGB Normalisation

The RGB normalisation button provides a simple colour histogram equalisation method. It scales each of the currently loaded red, green and blue fields so that their pixel values lie between 0 and 1, by dividing each pixel by the sum of the red, green and blue values. This can be useful for display purposes, as it will equalise the contrast in the colour fields and so bring out detail in dark regions. This function replaces the red, green and blue fields currently loaded into memory with their normalised versions.

It is an oft-repeated fallacy that colour normalisation (or histogram equalisation in general) should be used as a precursor to segmentation. In fact, since colour normalisation clearly cannot increase the information content of the original image, it should have no effect on the segmentation result if the segmentation algorithm is taking proper account of the noise on the data. If you see a paper or presentation which states that colour normalisation does affect the result of their segmentation, it means that their algorithm has an implicit assumption of scale (probably a blurring kernel of fixed size somewhere in the code). Stretching the colour space by normalisation therefore changes the scale of the space relative to the assumed scale, and so changes the segmentation result.

The Tina colour segmentation algorithm takes account of the noise on the data, by scaling all measurements in the colour space by the local noise. Therefore, applying colour normalisation prior to segmentation will have no effect on the segmentation result to first order. Some small second order ef-

fects may be observed, as the noise scaling is only performed locally, but for most natural images these should not change the number of labelled regions by more than about 10%.

16.3.6 Colour Segmentation

Tina provides an implementation of the colour segmentation algorithm described in more detail in Tina Memo no. 2001-015, as presented at the British Machine Vision Conference in 2001. The algorithm works by non-parametric density estimation in the JK colour space. The red, green and blue fields are converted to the IJK colour space, and the I field discarded to remove illumination effects to first order. The JK colour space is then mapped with points, where the distance between the points is specified by the "resolution" field. All measurements in the colour space are scaled by the local noise, in order to convert to first order into a variance-normalised space and so take proper account of the noise. Hill climbing is then performed on this map of nodes, moving from each pixel to the nearest node, and then across the nodes in the direction of steepest gradient, until a local peak is found. A list of peaks is generated, and each is assigned a label, in order of the number of pixels assigned to that peak. Then the labels are propagated to all pixels assigned to each peak, and an image of labels generated.

The novel aspect of the Tina colour segmentation algorithm is the way in which the peak climbing is performed. The points used to map the colour space can be envisaged as tesselating the space with a set of polygonal regions known as Voronoid cells: each point is at the centre of a cell, and the boundaries of the cell are defined by lines normal to the vector between each point and its immediate neighbours. During the hill climbing stage of the algorithm, each step from point to point is tested to ensure that it only steps to a Voronoid neighbour i.e. does not jump completely across a cell. This ensures that the hill climbing will not jump across local minima, without requiring any implicit assumption of scale (see the discussion in section 3.4). Combined with scaling all distance measurements in the colour space be the local noise, this ensures that proper statistical account is taken of the data accuracy.

The noise scaling in the colour segmentation operates by maintaining a matrix of errors on each pixel. This matrix is regenerated whenever an image is loaded, a colour field is popped from the stack, or a normalisation is performed. This error matrix is then used to perform error propagation through the colour segmentation algorithm. The noise estimation process operates on the assumption that the noise on each colour field is uniform when it is loaded into the tool. This should be a reasonably good approximation for most colour images. However, if you load a colour image and then manipulate a field within Imcalc, before popping it back into the colour tool, you could in theory transform the colour field such that the noise is no longer uniform. This will interfere with the operation of the colour segmentation function.

The Tina Colour Segmentation Algorithm

The results of the segmentation will be determined by the resolution of the points used to map the colour space, as selected in the "resolution" field. This specifies the distance between the points in terms of the standard deviation of the noise on the data. Its purpose is to blur the space according to the estimated noise, in order to avoid identifying noise fluctuations as statistically significant peaks. Entering 3 as the resolution (i.e. 3 standard deviations) will ensure that all statistically different regions are identified in most natural images. However, this will also identify regions such as coloured shadows. Whilst this is statistically the correct result, most people prefer a result which is slightly under-segmented i.e. small regions have been merged back into larger regions of similar colour, to avoid identifying coloured shadows etc. Therefore, the resolution can be raised to 5 or higher to eliminate these small regions. Raising the resolution will also significantly reduce the computational time required.

In order to perform a colour segmentation, ensure that an RGB image has been loaded. Then select the resolution as described above. Finally, press the "Segment JK" button. The resulting labelled image will be pushed to the top of the stack, and can be displayed in the Imcalc Tv. Binary images of individual regions can then be generated using the label selection functionality described below, and may be used for masking the original image. A mean colour version of the labelled image can be output using the "Mean Col Output" button.

Label Selection

Once the colour segmentation is completed, and the labelled image displayed in the Imcalc Tv, the regions covered by each label can be selected by entering the label in the "Label" field, and pressing "select". This will generate a binary image where the pixels having the desired label are white, and all other pixels are black, and place this image second on the stack for display in the Imcalc2 Tv. The number of pixels selected will also be displayed in the text window of the top-level tinaTool. The original labelled image will not be disturbed, allowing repeated application of this button for study of the segmentation result.

The binary images can also be used as multiplication masks. Use the store function in Imcalc to store a copy of the mask, and push each of the red, green and blue fields to the stack. Multiply each field by the mask in Imcalc, and pop it back into the Colour Tool. The masked colour image can then be output as a ppm image, providing a colourful way to display the segmentation result.

Mean Colour Image Output

Mean colour output is another convenient way in which to display the results of colour segmentation. It will take all of the pixels assigned a certain label, and replace them with their averaged red, green and blue values, outputting the result as a PPM file to the directory and file names specified in the relevant fields. In order to use this function, the labelled image must be on top of the stack i.e. displayed in the Imcalc Tv: then simply press the "Mean Col Output" button to generate the ppm image. Ensure that you change the filename to something other than that of the original image you loaded, as the function will not warn you about overwriting.

16.4 Quick Reference

- Tv choice: sets the current Tv. The colour tool has three Tv's: one each for the red, green and blue fields of the colour image currently loaded.
- Directory field: directory name (relative or absolute) from which to load a colour image.
- File field: filename from which to load a colour image. The extension (.ppm) must be explicitly included in the filename.
- Input ppm button: inputs the PPM image specified by the directory and filename. The image is split into three: one image for each of the red, green and blue fields, and displayed in the Colour Tool Tv's.
- Output ppm button: outputs the red, green and blue fields currently loaded into memory, and displayed on the red, green and blue Tv's, as a raw-format ppm image, with the directory and filename specified in the relevant fields.
- R Pop button: replaces the current red field image with the image from the top of the stack (i.e. the image displayed in the Imcalc Tool Tv). If blue or green fields are already loaded, then the size of the red field must be the same as that of the other fields.
- G Pop button: replaces the current green field image with the image from the top of the stack (i.e. the image displayed in the Imcalc Tool Tv). If red or blue fields are already loaded, then the size of the red field must be the same as that of the other fields.
- B Pop Button: replaces the current blue field image with the image from the top of the stack (i.e. the image displayed in the Imcalc Tool Tv). If red or green fields are already loaded, then the size of the red field must be the same as that of the other fields.
- RGB check and RGB Push button: pushes the current red, green and blue fields to the stack, for display in the Imcalc Tool Tv's. The check list specifies which images will be pushed, and they are pushed in reverse order such that the left-most checked field is top-of-stack. For example, if all three check boxes are selected, then the red image will be on top of the stack, the green image will be second, and the green image will be third.

- HSI check and HSI Push button: converts the red, green and blue fields to the HSI (hue, saturation and intensity) colour space, and pushes the results to the stack. The check list specifies which images will be pushed, and they are pushed in reverse order such that the left-most checked field is top-of-stack. For example, if all three check boxes are selected, then the hue image will be on top of the stack, the saturation image will be second, and the intensity image will be third.
- YIQ check and YIQ push button: converts the red, green and blue fields to the YIQ colour space, and pushes the results to the stack. The check list specifies which images will be pushed, and they are pushed in reverse order such that the left-most checked field is top-of-stack. For example, if all three check boxes are selected, then the Y image will be on top of the stack, the I image will be second, and the Q image will be third
- IJK Check and IJK Push button: converts the red, green and blue fields to the IJK colour space, and pushes the results to the stack. The check list specifies which images will be pushed, and they are pushed in reverse order such that the left-most checked field is top-of-stack. For example, if all three check boxes are selected, then the I image will be on top of the stack, the J image will be second, and the K image will be third
- Normalise RGB button: scales the RGB colour images such that all pixels lie between zero and one, by dividing each pixel by the sum of the red, green and blue values. The results replace the currently loaded red, green and blue fields. This function is of use only for display purposes, or to check that the segmentation is taking proper account of the noise on the data.
- Resolution field: specifies how far apart, in terms of multiples of the standard deviation of the noise on the data, the points used to map the JK colour space will be placed during the segmentation. A value of 3 will provide a segmentation identifying all statistically different coloured regions for most natural images. Increasing the value will speed up the segmentation and under-segment the image: this can be useful for removing the effects of coloured shadows etc.
- Segment JK button: performs a colour segmentation of the loaded image. Each statistically different region is assigned an integer label, in order of the number of pixels assigned to that region (the most populous region is assigned the label 0, the second most populous the label 1, and so on). An image of the labels is pushed to the stack, for display in the Imcalc Tv. The number of points used to map the colour space, and the number of different regions labelled, are displayed in the text window of the top-level tinaTool as ”no. nodes” and ”no. peaks” respectively.
- Label field and select button: produces a binary image of the specified label, where all pixels assigned that label are white, and all other pixels black. The labelled image produced by the segmentation must be on top of the stack i.e. displayed in the Imcalc Tv, prior to using this function. The resulting binary image is placed second on the stack i.e. displayed in the Imcalc2 Tv, allowing repeated application.
- Mean Col Output button: outputs a mean colour version of the segmentation result, where each pixel in the image is replaced with the mean red, green and blue values of all the pixels having the same label. The labelled image produced by the segmentation must be on top of the stack i.e. displayed in the Imcalc Tv, prior to using this function. The result is output as a ppm image with the directory and filename specified in the relevant fields.

Chapter 17

SmartROI Tool

17.1 Introduction

The SmartROI Tool is designed to support the automatic location of arbitrarily complex polygonal boundaries in grey level image data to an average spatial accuracy of about one pixel. The software supports a range of techniques covering conventional snakes and the more sophisticated deformable template models. The specific choice of algorithm applied can vary between these extremes allowing either shape constraints with a conventional location potential for simple tasks, or averaged or covariant models of grey level profiles for more complex tasks. These techniques are based on eigen-vector approximation to sample data sets. The most appropriate (and most accurate) technique will be problem specific and a separate model will need to be built and tested for each task. The model construction process is often manually intensive, but once a trained model has been developed they can be applied automatically and are often accurate and reproducible as a way of identifying regions in images. These techniques have thus found considerable use in recent image processing tasks.

In order to provide a simple but generic approach it is necessary to make some assumptions regarding the expected model and its construction. The basic assumption here is that the model to be located is a simple region consisting of an inner and/or outer portion and that there is a preferential orientation axis and overall scale. We also assume that sensible landmarks can be located, either along perpendicular lines at regular intervals along the axis or radially. This model has been chosen on the basis of simplicity and has a surprising range of applicability for the majority of basic image location tasks. In particular, the use of a main axis and perpendicular line mark-up considerably simplifies the representation of the shape model by ensuring that unwanted errors in the 2D location of the set of points are efficiently removed in the eigen vector model.

The number of allowable degrees of freedom is limited to ten for simplicity of interface design, though the number required for most tasks is often considerably smaller than this. Once an object has been located the region of interest defined by the polygonal boundary is then available for use in any **Tv**, including the **imcalc Tv**, thus allowing simple region extraction via the **Imcalc Tool roi** facility for further processing and automated data extraction.

17.2 Graphical Display and Data Selection

The **current tv** specification determines which image data the smartROI tool will process (this is selected using Tv choice menus in other tools). This choice will mainly depend on the preferred configuration for analysis of particular data sets eg: temporal data and volumes should use **sequence** and simple static images should use **mono** or **imcalc**. Problems based on simple snakes which require the calculation of image potentials should preferably use the **imcalc Tv**, while also using the **Imcalc Tool** to construct the image potential. This process can be flexibly automated using the macro replay facility (see the **Macro Tool**).

17.3 Profile Input/Output

The **polyname** field is used for multiple tasks and file name extensions are appended depending upon the task. The current boundary mark up can be saved to the **polyname** (with no extension). Individual mark up files can be loaded and the boundary will be displayed in the specified **Tv** with the selected display options. Results of model building are stored to the files **filename.mml** and **filename.pca**. These are automatically written when the model is built (see below). Construction of the model requires a list of markups. This list is held in simple asci format in the file specified by **filename.blt**.

17.4 Mark-up

All deformable template approaches require the manual construction of example data sets with which to build the parametric model of shape and grey-level profile. The parameters of this boundary can be specified using the **Model Parameters** dialogue box, and these must remain fixed across any set of data intended to construct a single model. The selection of a particular mark-up type **p axis r axis** initiates the process of mark-up in the selected Tv. Mark-up requires a three button mouse. The user must define how many boundary boints are required and may place them where he/she wishes, provided that each mark-up is consistent. Template models cannot deal with redefinition of point order within a training set.

For **p axis** mark up, click and drag the left mouse button to establish a baseline. Now click the left button and drag again to define a rectangular boundary box around the required structure. At this point a set of parallel lines will be drawn across the region, one of which is blue. This blue line can be used as a guide for the location of a boundary point. Mark-up using the **r axis** scheme is similar, specifying the left and right points on the main axis and continuing in an anti-clockwise direction filling in the missing points.

- **make** The grey level profiles will be sampled from the selected image. When the mark-up is complete and all data points have been fully specified, output the boundary data to **polyfile**. This data is written out along with the rotated and scaled co-ordinate of the 2D model for use in later stages of model building.
- **outer2roi**

  This button takes the outer profile (if present) and converts it to a polygonal boundary for use as the region of interest in any *Tv*.
- **inner2roi**

  This button takes the inner profile (if present) and converts it to a polygonal boundary for use as the region of interest in any *Tv*.

17.5 Training

- **build pca**

  In order to generate a new template model, construct a simple asci file ("filename".blt) containing the file names of the required profiles. Specify the name of this file in the **filename** parameter. The model is built using the **PCA** routine and is automatically output to the **filename** model files on completion.

  The eigen values for the shape model will be written to the main **tinatool** dialog window and can be used as a guide for subsequent selection of the number of principle modes. As scale does not appear as a mode of variation zero modes of variation corresponds to scale change only. At the same time the mean shape and profile model is written to the selected single model file. The model builder will use the options **Normlise** and **Global** if selected.

  The **Normalise** option is intended for image data which has variable greylevel scale.

Use of a **Global** correlation model between both shape and grey-level parameters. This model choice has considerable computational overhead and should only be selected for problems where there is a strong correlation between grey level profile and shape and the location problem proves to be particularly challenging. Accurate determination of the extra covarinace parameters also requires significant increases in the size of the training data set.

- **input pca**

  The file ("filename".pca) which stores the parameters of a specific trained model are specified by the **filename** parameter string. The mean model ("polyname".mml) can be loaded from **polyname** using **input**. This flexibility is provided so that the allowed variation specified by the PCA parameters for one dataset can be quickly applied to another.

17.6 Search

Once a model has been built or loaded, an instance of the model can be located in the selected **Tv** image using the **search** function, which executes a downhill simplex optimisation to search for the model in the image which minimises the selected **Cost** function. Executing this routine again continues the search from the last solution.

Generally when automating the final system there is no harm in executing the search process twice to try to ensure convergence. Searching for objects through temporal sequences of images generally benefits from starting each search from the previous solution. Coarse to fine searches for both snakes and deformable templates and any permutation can be supported using the macro facility and combinations of trained models. Multiple objects or disjoint or hinged structures should be located using separate trained models for each section.

- **prof**

  This option makes the selection between snake localisation and localisation based on grey-level profiles. Simple snake location operates via optimisation of an image potential using the specified shape parameters only. When specifying this option the local image potential is optimised along a line tangential to the control points. When locating a grey-level template, the local template profile is acquired tangential to the local boundary. If this is not the case check the order in which the boundary points were specified for the model (see above).

- **mse**

  Use of mean squared error (mse) or sum of absolute differences (a robust statistic).

17.6.1 SmartROI Parameters

The parameters which control the model search process are in the **SmartROI params** dialog box.

- The **init** facility will reset the variables which define the offest from the mean model to zero. (ie: setting the model a specification back to the mean).
- The total number of mark-up points defining the current model is specified by **No.Points** parameter. The model supports inner profile points as those points not included in the outer boundary **No.Points - No.Outside**.
- The **Profile** parameter specifies the length of the grey-level profile used for modelling.
- Location of the model (mid-point of the main axis) is specified by the **tx** and **ty** parameters. The search step used during initial localisation in the Simplex algorithm are specified by the **dtx** and **dty** parameters.
- The **scale** and **theta** parameters specify linear axis size and orientation of the current boundary profile. The search step used during initial localisation in the Simplex algorithm are specified by the **dscale** and **dtheta** parameters. Localisation is also restricted to lie withing the specified **s min**, **s max**, **th max** scale and orientation ranges. If the initial scale estimate is outside this range

then the routine will not optimise and the location will be returned unmodified. This can often be a source of problem when using the software for the first time and should be watched out for.

- The N offest shape eigen values are specified by the enumerated variables together with their search step parameters **dw**N. The effect of changing a parameter can be directly visualised by **redraw**ing the relevant **Tv**.
- The initialiseation of the Simplex algorithm is determined by the **Initial Search** parameters. Large values will result in quicker but less precise localisation.
- The specified model will have **mdl modes** shape eigen vectors and **prf modes** grey level eigen vectors. A value of zero corresponds to mean value only.
- The maximum variation of the model away from the mean model is limited by the **Search Limits**.

17.7 Typical Use

The **Template Tool** has been designed for use in automatically locating deformable shape in noisy (generally medical) data sets. Using it well requires some familiarity with the data and a little thought as to what is inteneded to be achieved. Start by determining the best number of points needed to accurately define the boundary of the object and whether an inner contour is also needed. Then experiment with the data to try to determine a reproducable way of positioning boundary points as accurately as possible. draw up guidelines for features to look out for and try to define the location of the boundary in a simple and reproducable manner. If necessary images can be pre-processed by the **Imcalc Tool** in order to assist with this process (eg: enhancing edges etc.).

Once you have a good idea of what you need to do then proceed with the following steps;

- Mark up a series of example profiles which you feel span the space of possible shape and grey-level variation. The number you will need will depend upon the degree of variability in the data and cannot be specified a-priori, but even simple models will generally require tens of examples.
- Construct a list file for the data you wish to include in the model and train using the PCA model builder. Use a maximum number of parameters without global covariance at this stage.
- Test the performance of the model on unseen datasets varying the number of parameters used for the shape and grey level variation. Try to use a minimum of the number of shape parameters which accurately locates the boundary. Investigate the effects of robust optimisation and data normalisation to see if there are any particular advatages to using these options. Always take care that you use the same cost function options during search that were used during PCA analysis and never attempt to search with more parameters than were written out during the analysis process (you can however use fewer).
- Test on additional datasets and if the location process repeatedly fails for a particular image mark it up and add it to the dataset for retraining.

As the set of example datasets increases the posibility of building a global shape and grey-level model improves. This should only be done if the model has repeated difficulty with locating data. Eventually, (generally quite soon) you should converge on a model which gives reliable performance for this data set. Accuracies of better than a pixel are generally achievable at all points around the contour with some care even in noisy data. Significantly better performance than this is generally difficult to achieve due to the restrictions imposed by the eigen model and algorithms such as edge detection may be better if there is low noise and strong edge data is available. These techniques should not be considered as competetors among the solutions for boundary location. It is even acceptable to use templates in order to locate edges for more accurate measurement.

The posibilities for data pre-processing before building the deformable model are limitless, but an attempt should be made to work with data-sets which have the properties of uniform random errors (see the **Imcalc Tool**) as this is what is most consistent with the statistical assumptions behind greylevel modelling and least-squares based location algorithms.

The resulting tracking variables of orientation, scale and principle eigen-modes of the shape model make a good starting point as a reduced representation of the data for any subsequent anaysis such as statistical pattern recognition (eg: classification). However, these techniques are not expected to form the basis of a generic object recognition system as there is no robust way of automatically selecting or building models for arbitrary image data sets.

Chapter 18

NMR-Segment Tool

18.1 Introduction

The purpose of the NMR segmentation tool is to evaluate methods for the segmention of tissue types in MR images and image sequences. Methods are provided for the analysis of single or multiple MR images. Density models of grey-level and image slope are constructed and used to compute the most likely voxel volume contents (Tina memo 2003-005).

The algorithm is a Bayes classifier which uses the parameters determined using an EM process (Tina memo 2001-005) (or from by the file provided) to compute the likely composition of each pixel (voxel). The method assumes that pure tissues have grey levels drawn from a Gaussian distribution and that partial volume effects are linear. These assumptions are reasonable for the majority of NMR scans in healthy subjects. The algorithm is stabilised (taking direct account of expected image noise) in the regions of pure tissue and guaranteed to give probabilities between 0 and 1, but may give systematic positional biases for some image types due to tissue boundary/noise ambiguity (Tina memo 2001-009).

18.2 Tool Description

Model File The specified text file is used for intialisation of the segmentation parameters and for storage of results. The format of the file supports arbitrary numbers of tissues and partial volumes and image slope density parameters if they have been determined (see above).

input loads a new model parameter file (See Appendix).

output outputs a new parameter file.

18.2.1 Multi-D fit

Routines for the estimation of model parameters.

hfit-scale executes a simplex minimisation to estimate the parameters of a composite density model for the grey level frquency distribution (histogram). It operates on the image currently displayed in the **Sequence Tool** within the specified region of interest (ROI). The model comprises Gaussian peaks for pure tissues and uniform distributions for partial volume mixture pixels. Relative mean grey level and distribution widths are taken from the specified *Model File.* Optimisation then determines an overall scale and initial normalisation parameters. Other parameters are to be determined via use of the Expectation maximisation algorithm via the algorithms described below. The user is advised to histogram the image in order to ensure that fitting has been sucessful.

E-step computes the probability that each voxel is consistent with the fitted model based on grey level values only (Expectation step of the EM algorithm).

E-step grad computes the probability that each voxel is consistent with the fitted model based on grey level and image slope values (Alternative Expectation step of the EM algorithm).

M-mean computes new estimates for the mean grey level parameters of the model.

M-cov computes new estimates of the covariance terms for the model.

M-prior computes new estimates of the density parameters for the model.

M-k grad calc computes new estimates of the slope parameters for the model (Tina memo 2004-009).

prob

18.2.2 EM plot

The **Tissue Type** string variable is used to select the tissue type for static segmentation, (eg: CSF, WM in the given example).

Routines for model visualisation and the analysis of image data for generation of tissue maps and simulated (noise filtered) images.

1D hist Plot data for image displayed n the **Sequence Tool** *Tv* for the selected region of interest. Superimpose the current estimate of the density model.

2D hist Generate scatter plot image displayed n the **Sequence Tool** *Tv* and the next in the sequence. Place this image and the estimated 2D density distribution from the model in the **Imcalc Tool** for viewing.

model Generate a noise free estimate of the image from the complete set of probabilistic estimates of most likey tissue proportions within each voxel. This process is likely to remove both noise and low level second order physics artefacts such as coil-inhomogeneity and flow hyperintensities (Tina memo 2003-007).

prob computes the most likely tissue volume probabilities and places the resulting image “map” on top of the stack for viewing by the **Imcalc Tool**.

18.2.3 Multi-D Grad Calc

Routines for the qualitative evaluation of slope parameters.

slope image computes an image of sum squared image gradients normalised to image noise and places it on the image stack for view in the Imcal Tool*Tv*..

scat plot produces a scatter image of multi-dimensional normalised gradient verses grey level (for current **Sequence Tool***Tv* image).

grad model produces a image of computed probability densities for multi-dimensional normalised gradient verses grey level (for current **Sequence Tool***Tv* image).

18.3 Image segmentation example

One common use of the segmentation routines is to identify particular tissues to be used as a mask for analysis of other data sets.

1. construct and load an inital estimate of model parameters using the format gven above.
2. Calibrate the tissue density model using **hfit-scale**, **E-step**, **M-mean**, **M-cov**, **M-prior**. Check the adequacy of the fit in the **Imcalc Tool graph** Tv via use of the **1D hist** plotting routines. Iterate this process until convergence.
3. Select the required probability map and compute it using **prob**.
4. Threshold the resulting probability map in the **Imcalc Tool** as required.

5. Manually select connected sub-regions with the **mark->connect** mouse tool.
6. Mulitply binary mask with other registered images of the same subject in order to identify data within the required region.

Tissue segmenatation using the **rusinek** process is similar but requires two **hfit** operations in order to determine the full set of mean tissue values.

Chapter 19

NMR-Analysis Tool

19.1 Introduction

The purpose of the NMR segmentation tool is to extract quantitative measurements from temporal MR sequences. This includes applications for the measurement of blood flow, perfusion and BOLD analysis (Tina Memo 2001-001).

19.2 Tool Description

This section describes the purpose of each function in the tool and the associated parameters.

Basic data interaction is provided at the top of the tool.

measure allows the size of regions within the image to be measured. **line** allows a line to be drawn, returning its length. **region** allows a polygonal region to be defined, returning its area. **mask** calculates the area of the binary mask image. The units of these measures are either mm or pixels depending on whether image scale information is available. (Currently mm are only available for NEMA image types).

The **plot** functions produce graphical information from pixel locations. **flow/T** produces a flow-time graph at the pixel location, **conc/T** produces a concentration-time graph for the pixel and **gamma fit** fits a Gamma variant to the data. More detailed explanations of these functions are given in below. For each of these functions an image calculator graph tool must be available and dynamic timing information must have been loaded.

The next row of buttons **stim params**, **gamma_params** and **perm params** provides dialog boxes for the subsequent analyses.

19.2.1 stim params

The **Stimulus** variable choice is used to select the correlation function for segmentation. Selection of a "**Stimulus**" generates a graphical plot of the stimulus function currently in use (provided that an image calculator graph window to be available). The parameters *period/2*, *offset*, *iphase* and *deadtime* are used to control the caracteristic shape and timing of the time curve. **SQR** is a normalised square-wave with duration and initial phase set in the **Stim Params** dialog box. **ROI** allows the user to define a function from the image sequence. The user marks the region on the image visible on the **Sequence Tool Tv**, typically the first image in the sequence. Clicking on the **ROI** button causes the program to build the correlation function using the image data within this region. On each frame in the sequence the average of the grey-level data within the ROI is calculated and used as the function sample. Once complete the mean of the function is computed and then subtracted from each sample and finally the function is normalised such that its $L2$ *norm* sums to 1.

period/2 specifies the half-wave length of the square wave function **SQR**. The **offset** variable specifies the range of the phase search to be performed during the correlation. A value of 0 specifies in-phase detection only, i.e. no phase search. A value of 1 indicates that shifts of -1, 0 or +1 are allowable and similarly for other values. The correlation function is always performed over the minimum amount of data. **iphase** specifies the initial phase of the square-wave either +ve or -ve. The actual value is not important as the square-wave function is both mean subtracted and normalized.

19.2.2 gamma params

The parameters in this dialog menu provide inital estimates and scalings for the bolus fitting based perfusion analysis. The *minimum t*0 parameter defines the region of the data (stating from zero) used to determine the basline for the fitted curve. The *recirculation cut* parameter is used to define the cut off point for the curve fit (proportion of peak height) used to eliminate recirculation effects. The *recirculation period* parameter is used to define the interval over which excess of data due to recirculation is estimated.

The **region fit** button can be used to estimate *average mtt* and *minimum t*0 from all of the data within a region defined by a binary mask stored on the top of the *imcalc stack*. This should be done prior to regional analysis.

19.2.3 perm params

This dialog box specifies a data file used to calibrate permeability analysis, the constraints used during fitting and parameters determined by the calibration process.

19.2.4 Sequence

The **Sequence** choice variable is used to select the method of correlation. The mathematics behind these functions is described in the Tina memos and published papers.

compare initiates the comparison between the image sequence and the stimulus function. The stimulus function is selected using the stimulus choice variable and the comparison performed using the technique selected with the sequence choice variable. The resultant correlation image is stored on the top of the image stack.

The 3 correlation functions are given below. I_i is the image pixel at the current location taken from the i'th image from a sequence of N, μ_I is the mean value of the I image sequence, S_i is the i'th value from the stimulus function and μ_S is the mean value of the stimulus sequence.

$$\text{GBAM} = \frac{1}{N}\sum_i^N (I_i - \mu_I)(S_i - \mu_S) \tag{19.1}$$

$$\text{STIM} = \frac{\sum_i^N (I_i - \mu_I)(S_i - \mu_S)}{\sqrt{\sum_i^N (I_i - \mu_I)^2 \sum_i^N (S_i - \mu_S)^2}} \tag{19.2}$$

$$\text{FRIS} = \frac{\bar{S} \cdot \bar{I}}{\sqrt{\frac{1}{N}\sum_i^N (I_i - (\bar{S} \cdot \bar{I})S_i')^2}} \quad \text{where } S_i' = \frac{S_i}{\sqrt{\sum_i^N S_i^2}} \tag{19.3}$$

These statisitcal measures are our interpretation of the basic statistical approaches taken in the GBAM software, STIMULATE software and by Friston's group (Tina memo 2001-002).

19.2.5 Perfusion

This set of buttons provide regional analysis of temporal sequences for the extraction of quantitative parameters relating to voxel level perfusion of tissue. It is intended to provide support for the "net-flow" approach developed in our group (Tina memo 2001-003).

gamma fit fits a gamma variant to the timecourse at each pixel location. The selected parameter choice option **TTM, CBV, MTT, ERR, RCC** (time to mean, cerebral blood volume, mean transit time, residual error, or recirculation contribution) determines which of these parametric descriptions of the data to push onto the *imcalc stack*. The initital execution of **gamma fit** requires several seconds of processor time. All parametric images reulting from that analysis are subsequently available instantly upon selection with the choice menu.

19.2.6 Permeability

This set of buttons are used for the quantitative analysis of tissue permeability based upon the first pass method developed in our group. Action of the buttons is similar to the use of the perfusion technique, except the data also requires estimation of flip angles (**Flips** and absolute calibration **T1 Calib** before parametric images can be computed using **perm fit**. This software is still under development and latest developments have not been fed back into the library.

19.2.7 Test

These functions are auxiliary to the main operation of the tool. Only an outline of functionality is given as these functions are liable to change.

flow/t plots the average flow-time graph for the regions shown in the binary mask image at the top of the stack. For each image in the sequence the pixel intensities over the region are averaged and the result is plotted together with the corresponding scan time information.

report generates a patient report of the form shown below.

19.3 Sequence images segmentation examples

19.3.1 FMRI example

It is assumed that the FMRI sequence has already been loaded into the **Sequence Tool**. Ensure that the **NMR Analysis Tool**, the **Imcalc Tool** and two **Tv** tools (one installed under sequence the other under imcalc) are all open.

1. Ensure that dynamic timing information is present.
2. Check the **Stim Params** options. Ensure that the **hwave** option correctly specifies the length of the expected 'on' and 'off' periods. Change the value of **offset** to the range of phases to be searched, or zero if no phase shifting. Check the **iphase** parameter and ensure it represents the correct start phase.
3. Click the **SQR** option on the Stim. Func list.
4. If desired, open another **Tv** and install it under imcalc graph. By changing the params, and clicking **SQR** the stimulus function can be modified until the correct form of the function is visible.
5. Select the desired correlation function from the **Sequence** correlation function list.
6. Click **compare** to initiate the comparison. The resulting correlation image will be placed on the top of the image stack and will thus appear in the imcalc **Tv** window.
7. The correlation image may then be thresholded to extract a binary mask of the 'active' and 'inactive' regions using the **thres** button in the **Imcalc Tool**.

8. The **mask->connect** mouse option can be used to identify those activations regions of interest, cleaning the mask.
9. The activation mask can be reused to define the stimulus function by selecting **Mask** from the **Stimulus** options. In this way, the square-wave function can be used to 'bootstrap' the process. Once a rough estimate of the location of activations is found, capturing examples using masks reduces the restrictions of comparison to specific mathematical functions and provides an estimate of the most significant response curve in the temporal data. This process can be interpretted as an principle component analysis using the power method.

19.3.2 Perfusion segmentation example

It is assumed that the FMRI sequence has already been loaded into the **Sequence Tool**. Ensure that the **NMR Analysis Tool**, the **Imcalc Tool** and two **Tv** tools (one installed under sequence the other under imcalc) are all open.

1. Load an imagefrom the temporal sequence into the **Imcalc Tool**.
2. Binarise the image in order to specify the required analysis (fit) region.
3. Use the **region fit** process to determine suitable global fitting parameters.
4. Fit all data using **gamma fit**.
5. Select additional parametric images as required using the **Perfusion** choice menu.

Chapter 20

Coreg Tool

20.1 Introduction

The TINA coregistration tool automatically calculates the rigid transformation required to align two images or image volumes of the same scene. For instance, if two MR volumes of a subject's brain have been acquired during different scanning sessions, then the coregistration tool allows the user to automatically calculate the transformation model required to align the volumes, allowing direct regional comparisons. The tool is divided into two panels: the "simple coreg tool" and the "advanced coreg tool". The results of the alignment are displayed in a dialog box, the "AIR Parameters Dialog Box". Coregistration results can be stored in binary AIR files: the format is compatible with an automatic image registration program called AIR.

20.2 The Simple Coreg Tool

20.2.1 Loading Data

Data for the coreg tool is loaded via the sequence tool. Two volumes are required: the target image volume i.e. the volume the user wishes to align to, and the source image volume i.e. the volume that will be manipulated with the parameters of the transformation model in order to align it to the target image volume. The Tina coreg tool calculates rigid coregistrations, where the transformation model consists of a translation, a rotation, and a scaling. Since this tool is used primarily to align medical data sets, which typically consist of $\approx$ 100000 voxels, the maximum physically-meaningful accuracy can be achieved with only a small sample from the data. Therefore, in order to reduce the processor time required to perform a coregistration, only the data on three orthogonal planes projected through the image volumes are used. These three planes are specified by the centre point at which they intersect, which is entered by the user into the "Centre x, y, z" fields in the simple coreg tool, and are displayed in the three coreg Tvs.

In order to load the data, first specify the target image volume and load it via. the sequence tool. Then specify the centre point in the simple coreg tool. Press the repaint button on any of the three coreg Tvs to display the three projected planes. The aim is to select three data-rich projections: alter the centre point and repeat until satisfactory projections are obtained. Setting the centre point to the actual centre of the image volume is usually a good starting point, although it should be remembered that the alignment will be most accurate at the centre point, and so if the intention is to examine only a particular structure, a centre point within that structure should be specified. Non-integer values can be entered as a centre-point: the projections displayed in the Tvs will be produced using the interpolation routine specified by the "Reslice" choice fields (described below). Tri-linear interpolation (the "linear" choce) is typically used as this represents a reasonable compromise between speed and accuracy.

Once satisfactory projections have been obtained, the data is latched. This stores the three projections displayed in the coreg tool Tvs, allowing a second image volume to be loaded. Latching of the data is achieved using the "Latch" choice fields. These also specify how the data will be displayed when both

volumes are loaded. The choices are

- image: Display only the three planes through the image volume currently loaded into the sequence tool. If both target and source volumes have been loaded, selecting this choice will discard the target volume, resetting the coreg tool.
- anaglyph: Display the target images in green and the source images in red.
- edges: Display the results of edge detection on the two image volumes (target image edges in red, source image edges in blue) on top of the planes projected through the current sequence.
- chequer: display a chequerboard pattern, with alternating squares taken from the source and target volumes.

The "anaglyph" choice is typically used. When the data is latched, the threshold specified in the AIR parameters dialog box is applied: voxels with grey-levels lower than the specified value will be ignored during the coregistration. This can be used to ignore the air space around MR images with certain modalities, or to eliminate certain image structures. However, in general the threshold should be set to a value lower than the lowest grey-level in the image volumes, ensuring that all of the image data is used in the coregistration. When the latch choice is selected, the number of voxels in the image volume that lie above the threshold will be displayed (as a percentage of the total number of voxels) in the text window of the top-level tinaTool. The displays in the three Tvs will also show the voxels that lie above the threshold in green, on top of the three projections in black-and-white, showing which structure have been selected.

Setting the latch choice back to "image" at any point will discard the three projections through the target image volume stored in memory, and display the three projections through the image volume currently loaded into the sequence tool in the three coreg Tvs. This resets the coreg tool. If no sequence is currently loaded in the sequence tool, empty images will be displayed in the coreg Tvs.

Once the target images have been latched, the source image volume can be loaded via. the sequence tool. Pressing repaint in any of the three coreg Tvs will display the source image volume on top of the target image volume using the method specified by the latch choice. For example, if "anaglyph" was selected, the target image volume will be displayed in green, and the source image volume in red.

20.2.2 Manual Coregistration

Coregistrations can be preformed manually using mouse interactions with the three coreg tool Tvs. Once both data volumes have been loaded, select the "Mouse" drop-down list using a right mouse click in the Tv, and select "zoom" using a left mouse click. Do this in all three Tvs in turn. The source image volume can then be manipulated using the mouse. With the mouse pointer over one of the Tvs, holding down the left mouse button and moving the mouse will translate the source image with respect to the target image. The source image will be displayed as a wire-frame during this process. On releasing the left mouse button, the displays in all three Tvs will be updated with the translation that has been applied. The centre mouse button will apply rotations in a similar fashion (this functionality may be achieved by holding down both the left and right mouse buttons on certain systems with two-button mice). The right mouse button applies scalings. Right click and hold, then drag to draw a rectangle. The source image volume will then be scaled to fit inside this rectangle: if the rectangle lies within the Tv, the source image volume will be scaled down; if the rectangle is bigger than the Tv, the source image volume will be scaled up.

20.2.3 Automatic coregistration

Tina can perform the coregistration automatically, by optimising a metric that measures the similarity between the two image volumes. Two metrics are available: a chi-squared metric based on the alignment between edges in the images, and the Mutual Information measure (see Advanced Coreg Tool below). Both methods use simplex optimisation, a local optimiser. Therefore, a rough alignment must be achieved before automatic coregistration, to prevent the optimisation becoming trapped in a local minimum. This

rough alignment can be achieved either by hand, using the manual methods specified above, or by loading in an AIR file specifying the rough alignment. If such an AIR file exists, enter its path in the AIR file field, and press “input” in the simple coreg tool. The values specified in the AIR file will be loaded into the AIR parameters dialog box, and the result of applying the transformation to the source image volume will be displayed in the three coreg Tvs.

Prior to coregistration, select the degrees of freedom that the transformation model will possess using the “Model” check buttons. Any combination of translation, rotation and scaling can be selected.

During coregistration an interpolation algorithm is used to resample the source image volume on the voxel grid of the target image volume. The interpolation routine to be used is selected via. the “reslice” choice buttons. The routines available are

- Nearest: take the grey-level value of the voxel in the source image volume that is closest to the required position
- Linear: performs a trilinear interpolation
- Sinc 5/7: performs renormalised sinc interpolation (see Tina memo 1999-005) using either a 5x5x5 or 7x7x7 voxel kernel.

The chosen interpolation algorithm is also used to produce the displays in the three coreg Tvs, to produce the stored projections through the target image volume when the data is latched, and to reslice the data after coregistration (see below). In general, tri-linear interpolation provides a good balance between speed and accuracy. Sinc 5 interpolation will produce a more accurate result, but will require several minutes to perform an automatic coregistration. Note that the interpolation method can be freely changed at any time: for instance, the coregistration can be performed using tri-linear interpolation for speed, then the data can be resliced using sinc5 interpolation for increased accuracy.

In order to perform the automatic coregistration, press the “Coreg” button. The edge-based similarity metric will then be optimised, the resulting transformation model parameters displayed in the AIR parameters dialog box, and applied to the source image in the three coreg Tvs. The values of the similarity metric before and after optimisation, and the number of iterations performed, will be displayed in the text window of the top-level tinaTool. Due to the nature of simplex optimisation, more accurate results (subject to a law of diminishing returns) can be achieved by re-running the automatic coregistration (by pressing “Coreg” multiple times: little increase in accuracy will be obtained after the third run). The calculation of the similarity metric is dependent on the “border” and “blur” fields, described in the AIR parameters dialog box quick reference below. In general, a border of 5 is sufficient if a rough alignemnt has been achieved before coregistration. A “blur” scale of 0.75 should be used for images of the same scene (same subject for medical image volumes). Note that, although coregistrations can be performed between different scenes (different subjects) the statistical interpretation of the similarity metric in this case is questionable, regardless of the similarity metric used. The blur scale should be set to a higher value (typically 3) in such coregistrations to accommodate the differences between the data (the source image volume) and the model (the target image volume).

The edge-based similarity metric may have problems coregistering image volumes in which equivalent edges have opposite signs. This situation may occur when coregistering MR and CT images. In this situation, use the “Modality” choice in the advanced coreg tool (see below).

20.2.4 Outputting the Results

The results of the coregistration can be output in one of two ways: writing out a binary file (the AIR file) containing the transformation model, or re-slicing the source image volume. Pressing “push” in the simple coreg tool will output the projection through the source image displayed in the currently active coreg Tv: pressing “seq reslice” will reslice the whole source image volume, producing an image volume where the individual images are at equivalent positions to those in the target image volume. In either case the images will be entered onto the imcalc tool stack, and can be viewed in the imcalc tool Tvs. Pressing “stack-seq” will delete the current sequence and copy the results stored in the imcalc tool stack into a new sequence, which can then be viewed or output via. the sequence tool.

Alternatively, the transformation model can be output as an AIR file. Enter the desired path and filename, either as an absolute or relative path (relative to the directory in which the tinaTool was run), into the "AIR file" field, and press output. AIR files are in a binary format, and are compatible with the image registration program called AIR. AIR files can then be input in later sessions by pressing "input". The transformation model parameters can also be displayed in the text window of the top level tinaTool, with the rotation parameters displayed as angle cosines, by pressing the "dump" button.

20.3 Simple Coreg Tool Quick Reference

- Help button: spawns a text window containing online help for the coreg tool.
- Advanced button: spawns the advanced coreg tool.
- Tv choice: sets the current tv. The coreg tool has three tv's: x, y, and z, which display three planes through the data volumes, defined as the planes that pass through the location specified by the centre point. Only the data in these planes is used to calculate the similarity metric used in the coregistration.
- Centre: defines the point in the reference image volume at which the x, y, and z planes intersect (see Tv choice).
- Up/down buttons: adjusts the centre point by one voxel in the x, y, or z direction, as specified by the current tv choice.
- Factor: specifies a zoom factor between the two image volumes.
- Zoom button: repaints the Tvs according to the specified centre and zoom factor.
- Reslice choice: specifies the interpolation algorithm used to resample the source image volume on the voxel grid of the target image volume, both for display in the coreg Tvs, and for calculation of the similarity metric during optimisation. The choices are:
  - Nearest: take the grey-level value of the voxel in the source image volume that is closest to the required position
  - Linear: performs a trilinear interpolation
  - Sinc 5/7: performs renormalised sinc interpolation (see Tina memo 1999-005) using either a 5x5x5 or 7x7x7 voxel kernel.
- Push button: pushes the image displayed in the current coreg Tv to the imcalc tool stack.
- Latch choice: specifies how the coreg Tvs display the volumes. The choices are as follows:
  - image: display only the three planes through the image volume currently loaded into the sequence tool. If both target and source volumes have been loaded, selecting this choice will discard the target volume, resetting the coreg tool.
  - anaglyph: display the target images in green and the source images in red.
  - edges: display the results of edge detection on the two image volumes (target image edges in red, source image edges in blue) on top of the planes projected through the current sequence.
  - chequer: display a chequerboard pattern, with alternating squares taken from the source and target volumes.
- Model choice: specifies the degrees of freedom included in the transformation model: and choice of translation, rotation and scaling can be selected.
- Seq reslice button: reslice the current sequence according to the transformation model (see model choice), using the specified interpolation algorithm (see reslice choice), pushing the results to the imcalc stack.
- Seq norm button: apply coil inhomogeneity correction (see Tina memo 2000-004) to the current sequence: this is specific to MR data.

- Set scales button: apply the scales specified in the sequence tool to the current image volume.
- Coreg button: perform the automatic coregistration, applying simplex optimisation to a chi-squared measure of similarity between the edges in the two image volumes. The parameters of the optimised transformation model are displayed in the AIR parameters dialog box, and applied to the source image volume displayed in the three coreg TVs. The value of the similarity metric before and after coregistration, and the number of loops performed in the optimisation, are displayed in the text window of the top-level tinaTool.
- AIR file: specifies a file containing the transformation model. A binary file format is used.
- Input button: input a transformation model from the specified AIR file (see AIR file), load the values into the AIR parameters dialog box (see AIR Parameters Dialog Box), and apply the transformation to the source image, displaying the results in the three Tvs.
- Output button: output the current transformation model, as displayed in the AIR parameters dialog box (see AIR Parameters Dialog Box), to the specified AIR file (see AIR file). This can be used to store the results of the coregistration for future sessions.
- Swap button: invert the current transformation model. If the result of coregistering image volume A to image volume B has previously been stored as an AIR file, this button allows the result to be displayed with the data in the reverse order (i.e. a coregistration of image volume B to image volume A.
- Dump button: output the current transformation model to the text window of the top-level tinaTool, displaying the rotation as angle cosines rather than a rotation matrix.
- Params button: spawn the AIR parameters dialog box.

20.4 The AIR Parameters Dialog Box

The AIR parameters dialog box displays the current transformation model parameters, together with basic information about the image volumes. Manual alterations to the fields in this dialog box can be output to an AIR file, or applied to the volumes displayed in the coreg tool Tvs by pressing the “zoom” button in the simple coreg tool. The meaning of each field is given in the quick reference section below.

20.5 AIR Parameters Dialog Box Quick Reference

- Standard: the path of the target image volume.
- Dimensions: the dimensions (number of voxels in the x, y, and z directions) of the target image volume.
- Set S button: copy the path of the target image volume to the filename field of the sequence tool.
- Scale: the voxel sizes of the target image volume.
- Reslice: the path of the source image volume.
- Dimensions: the dimensions (number of voxels in the x, y, and z directions) of the source image volume.
- Set R button: copy the path of the source image volume into the filename field of the sequence tool.
- Data: value appended to the AIR filename.
- Dec button: decrement the data value (see data).
- Inc button: increment the data value (see data).
- Scale: the scaling matrix of the current transformation model.

- Er: the rotation matrix of the current transformation model.
- Et: the translation matrix of the current transformation model.
- Threshold: specifies a threshold applied to both the source and target image volumes: data with grey-levels lower than this threshold are not sampled either for display on the Tvs or calculation of the similarity metric. This allows the air space around MR images of certain modalities to be ignored.
- Blur scale: specifies the standard deviation of a Gaussian blurring applied to the data before calculation of the simple coregistration similarity metric, allowing the suppression of small-scale structure.
- Border: specifies a border around the source image volume (in voxels). Data from this region is not sampled during calculation of the similarity metric. The value should be set to the maximum expected offset between the two image volumes that will be applied during coregistration, ensuring that the region of image data sampled will not change due to changing overlap of the image volumes: this form of data stationarity is essential for the calculation of chi-squared similarity metrics (see Tina Memo 2004-001).

20.6 The Advanced Coreg Tool

The advanced coreg tool provides automatic coregistration using the Mutual Information (MI) similarity measure, together with access to lower-level parameters in the coregistration. Although the MI coregistration can be used by following the instructions below, the other functions available here are only used for research into coregistration. See Tina Memo 2004-001 for more information.

The MI coregistration has been found to give a more accurate determination of the parameters of a coregistration, particularly the rotation parameters. In order to invoke it, load and latch the target volume, then load and roughly align the source image volume, as with the simple coreg tool. Select the choices in the advanced coreg tool as follows:

- MI choice: Trad.
- Angles choice: either of the quaternion representations.
- Max. ig. choice: Off
- Limit bins choice: Off.
- Bin size choice: 1σ.
- Hist smooth: Gauss.
- Power: 4.0.
- Main peak remover: Off.

Then press the "MI auto" button to perform automatic coregistration using the MI metric in place of the edge-based metric. Note that the "modality choice" button and "blur" field do not apply to MI-based coregistration: the functionality of all other buttons and fields in the simple coreg tool and AIR parameters dialog box are unchanged.

Chapter 21

SeqROI Tool

21.1 Introduction

The purpose of this tool is to allow the user to mark around 3D structures (using closed 2D splines), to display these in 3D, and allow them to be saved and loaded, recording the volumes of the structures marked (see Tina memo 2000-010). These regions can then be applied to other processing in order to summarise parametric measurements within regions (or simply to measure volumes). The software is currently restricted to be able to manipulate up to 6 separate volumes.

In order for the tool to work properly the sequence of images you are considering needs to be loaded and shown in a *SequenceTv*.

21.2 Graphics and Splines

- **3D rep**

  To visualise the mark-ups in 3D, install a Tv using this button, to produce a *threed Tv*.

- **Draw Spline**

  Click on Draw Spline and then $Mouse->mouse$ in the *SequenceTv*. Use the left mouse button to position the points of the spline, and the middle button to create the spline.

- **Edit Spline**

  Click on **Edit Spline** and then $Mouse->mouse$ (if not already selected) in the sequence Tv. To move a point on the spline, click with the left button on the spline point and drag to required position. To insert a point, click with middle button; to delete a point, click on the point with the right button. Note that because of the spline representation, there must be three remaining points in the spline.

- **Remove Spline**

  Click on **Remove Spline** to remove the spline in the given VOI for that slice.

- **Import from Poly**

  If any Tv panel has a polygonal region of interest marked out, clicking on this button results in the poly_roi being converted to a spline and represented on the sequence Tv and threed Tv.

- **Export to Imcalc**

  Use this button to convert a spline to a poly_roi and display in the imcalc Tv. I think you need to have an image in the imcalc first.

21.3 The VOL: Buttons

It is possible to simultaneously display 6 different volumes of interest using the **SeqROI Tool**. Note that you need to decide which VOL you are in before you do anything. For example, it is not valid to have VOL=0, to draw splines, then click on VOL=1, and then print vol to save the splines, as Braintool will write out the splines in VOL 1, not the splines you've drawn in VOL 0.

- **Empty VOL**

  Removes all of the splines in the given VOL.

- **Print vol**

  This writes out an ascii file containing the volume of the voi, and details of all the control points of all the splines in the given volume. The file you write to is given by the Directory and Filename fields. Note that no extension is needed, Braintool automatically adds .voi# to the filename, where # is $0->5$, according to which VOL is chosen.

- **Read vol**

  This reads in the voi specified by the *Directory*, *Filename* and VOL fields, and displays the splines in the sequence and threed Tv's.

Chapter 22

The DODECANTS Tool

22.1 Introduction

Distinctive patterns of accelerated cerebral atrophy are a feature of a large number of neurodegenerative and dementing disorders such as Alzheimer's disease, frontotemporal dementia, Parkinson's disease and others. Therefore, analysis of these patterns can be used for diagnostic decision support. Cerebral atrophy can be measured either through measurements of grey and white matter volumes, or measurements of the volume of the cerebro-spinal fluid (CSF). Since the interior volume of the cranium is approximately fixed throughout adulthood, increases in CSF volume provide an accurate marker for decreases in grey and white matter volume. However, the CSF has markedly different physical properties to the other tissues present in the cranium, and so it is possible to design MR pulse sequences that exhibit high grey-level separation between CSF and the other tissues present. This leads to more accurate segmentation, and thus more accurate volume measurements, than would be possible if grey and white matter volume were measured.

The DODECANTS tool implements an updated version of the functionality described in [8]. The aim of the algorithm is to map the distribution of CSF volumes in the cranium at a coarse level, and to perform nearest-neighbour classification of the distributions in previously unseen dementia patients through comparison with the distributions in a set of patients for whom reliable diagnoses are available. These classifications can then be used for diagnostic decision support. Since the interior volume of the cranium is approximately fixed throughout adulthood, increases in CSF volume provide an accurate marker for decreases in grey and white matter volume. However, since the grey-level separation between CSF and the other tissues present is higher than the separation between grey/white matter and bone, fat and air, for the MR imaging sequence used here, the signal-to-noise ratio for the segmentation process is higher and thus the segmentation is more accurate.

The DODECANTS tool combines functionality from several other TINA tools. The analysis procedure consists of four stages: registration, segmentation, CSF volume measurement and normalisation, and nearest-neighbour classification. First, all images under analysis are registered to a consistent coordinate system using rigid Mutual Information (MI) based registration [1, 2], as described in TINA Memo nos. 2001-013, 2003-002 and 2004-001. Segmentation of the CSF is achieved using the EM-based algorithm described in TINA Memo no. 2004-009 [7] (see also [6], [4], [10], and [5] for details of the development and testing of this algorithm). The CSF volume maps are then multiplied with a set of binary masks, drawn by hand in the standard coordinate system. The masking has two purposes: to delete non-CSF fluid spaces (e.g. eyes, sinuses) and to enforce a consistent inferior boundary to the measurement space, defined by drawing a line in the mid-sagittal section parallel to the horizontal axis that passes through the junction of the calvarium and the tentorium cerebelli. The anterior, posterior, lateral, and superior boundaries of the CSF space are automatically identified by locating the extremes of the CSF.

The CSF space is then divided into twelve equi-sized rectangular volumes, defined by planes which divide the space into anterior, central and posterior thirds, lateral halves, and superior and inferior halves, and the CSF volume in each of these regions is measured by counting the number of pure CSF voxels, and the number of partial volume voxels containing more than 50% CSF by volume, and multiplying

with the voxel dimensions. The purpose of the arbitrary division of the space is to allow analysis of patterns of cerebral atrophy independent of any prior hypothesis regarding spatial distribution. The volume measurements are then normalised for variation in head size and for normal age-related atrophy, using the procedures described in TINA Memo no. 2004-002 [3]. Head size normalisation is accomplished by dividing all volume measurements by the total intracranial volume (TIV), computed from the volume of the bounding box on the CSF space as described in TINA Memo no. 2004-002, which is in turn computed by finding the product of the maximal extents of the CSF in the anterior-posterior, lateral, and superior-inferior directions. Normalisation for age-related atrophy is accomplished using a Weibull cumulative distribution functional fit to volume measurements from 70 normal volunteers, as described in TINA Memo no. 2004-002.

The twelve normalised CSF volumes are then used to calculate five reduced variables,

$$W_1 = \frac{\sqrt{M} - \sqrt{F}}{\sqrt{2}}$$

$$W_2 = \frac{\sqrt{M} - \sqrt{B}}{\sqrt{2}}$$

$$W_3 = \frac{\sqrt{F} + \sqrt{M} + \sqrt{B}}{\sqrt{3}}$$

$$W_4 = \frac{\sqrt{P} - \sqrt{S}}{\sqrt{2}}$$

$$W_5 = \frac{\sqrt{U} - \sqrt{L}}{\sqrt{2}}$$

where F is the sum of the anterior four volumes, M is the sum of the central four volumes, B is the sum of the posterior four volumes, P is the sum of the left six volumes, S is the sum of the right six volumes, U is the sum of the superior six volumes, and L is the sum of the inferior six volumes. Left and right in this case are the left and right sides of the image, not necessarily of the patient. These variables are relative, and so errors in certain stages of the analysis, such as misregistration, will be removed to first order by this procedure. Finally, a k-nearest-neighbour classifier is run on the reduced variables, using a leave-one-out approach to classify each data set on the basis of the others. These diagnoses can be used to estimate the sensitivity and specificity of the technique.

It should be noted that the DODECANTS tool is still under active development. Therefore, the interface has been optimised for maximum functionality, rather than simplicity, and so some of the procedures described below are complex. In addition, checks have not been implemented for all memory-related failure modes (e.g. missing input files), and so the tool may crash if misused.

22.2 Installation

In order to use the TINA DODECANTS tool, you must already have compiled and installed the TINA libraries. See the TINA website

```
http://www.tina-vision.net
```

for instructions. The DODECANTS tool forms a part of the main TINA distribution, and is included in the example 2 toolkit located in

```
tina-tools/toolkits/example2
```

Once the main TINA libraries have been compiled, cd into this directory and type

```
make
```

The DODECANTS tool includes functionality from an number of other TINA tools (the Sequence tool, the Coreg tool, the Imcalc tool and the Segmentation tool), and so information on the progress of the algorithm is displayed across a number of tool windows and TV's. In order to simplify the process of setting up the required windows, a macro has been produced. In order to use this macro, use the command

```
./tinaTool -f oct_test
```

in order to run the toolkit.

22.3 Use of the DODECANTS Tool

22.3.1 Preparation of the Data List

In order to allow rapid processing of large numbers of data sets, the DODECANTS tool requires a list of the data sets to be analysed as input. This list should be in text format and should look like the following:

```
No of data sets
Path to rough tair file
No of slices      Path to standard brain image
No of slices      Path to subject brain image
Label     Disease code      Age
```

"No of data sets" is the number of data sets in the list. "Path to rough tair file" is the absolute path to the file containing the initial starting point for registration (see Section 3.2 for details). "No of slices" and "Path to standard brain image" are the absolute path to the MR image volume used to define the standard coordinate system and the number of slices in that volume (see Section 3.2 for details). Following these three lines, there are two lines for each subject in the data list: "No of slices" and "Path to subject brain image" are the number of slices in the subject MR image volume and the absolute path to that volume; "Label" is a simple name that will be used to identify the subject in any output files; "Disease code" is a single digit code (from 1 to 9) used to represent the disease that the patient is suffering from; and "Age" is the age of the patient in years. An example is given below.

```
2
/home/pab/data/rough.tair
49 /home/pab/data/normal_brain
49 /home/pab/data/patient1
alice 4 61
49 /home/pab/data/patient2
bob 1 66
```

The label should be a text string with no spaces or special characters, and the disease code can be arbitrary: it will only be used to cluster the data in the KNN classifier and to prepare files listing the colours applied to each data point in the output files for display in xgobi. The colours applied are as followed:

```
1: SkyBlue
2: Orange
3: Red
4: Green
5: Yellow
6: Blue
7: White
8: White
9: Grey
```

Our in-house use of the labels is as follows:

```
1. Normal control
2. Alzheimer's disease
3. Vascular dementia
4. Fronto-temporal dementia
5. Lewy Body disease
6. Schizophrenia
7. Possible Alzheimer's disease
8. Possible Vascular dementia
9. Undefined
```

The DODECANTS tool requires that all of the data sets to be analysed are in ANALYZE format. However, TINA is capable of loading a variety of medical image formats and converting them. Therefore if, for example, you wish to analyse data in DICOM format, start a tinaTool, load each image volume into the Sequence tool, and output it as an ANALYZE image volume. Due to the restrictions of the ANALYZE format, the images must be cast to integer variable type prior to output: this can be accomplished by pressing the "int" button on the "image type" choice list in the sequence tool prior to output. See the TINA User's Guide for more information on the use of the Sequence tool

22.3.2 Registration

The DODECANTS tool is designed to use a single MR image volume to define a standard coordinate system, allowing comparisons of regional CSF volume measurements across groups of subjects. An in-house standard has been used in the work conducted within the TINA core group, and is available at

```
http://www.tina-vision.net/tina-main/tarballs/DODECANTS/atrophy_standard_brain.tar.gz
```

If users of the DODECANTS tool wish to use the same standard, then this data set should be downloaded and extracted, and the path to the norm.img file entered into the data list (without the .img extension). The file is in ANALYZE format. More widely used coordinate systems (MNI Brainweb, Taliarach etc.) could be substituted, but users should be aware that this will require preparation of the masks used in the segmentation stage. The masks required for the TINA standard are contained in the tarball listed above, in the file norm_bincut. These should be left in the same directory as the standard brain image.

The TINA MI registration algorithm uses simplex optimisation: since this is a local optimisation routine, a rough alignment of the source data set to the target is required as a starting point. The rough alignment should be prepared in advance, and the results stored in a .tair file, following the instructions given in the TINA User's Guide. It need only bring the images into a very rough alignment, and so can be prepared by manual rotation, translation and scaling. The rough alignment will be applied to all data sets listed in the data list: if the data sets are themselves very poorly aligned (e.g. consist of a mixture of data sets with axial and coronal slices) then they should be split into several data lists, and analysed separately.

Once the rough .tair file and data list have been prepared, the registration stage can be started. Run a tinaTool as described above, using the oct_test macro to set up the required windows. Then enter the path to the directory into the "Directory" field of the DODECANTS tool, and the name of the data list file (with any extension) into the "Data List" field. Then press the "Bulk Coreg" button in the DODECANTS tool. The tool will then run through all data sets listed in the data list, registering them to the standard brain image, and writing the results to .tair files in the same directory as the original data. For example, if the data set is called alice.img, the registration result will be called alice.tair_mi.

Since the registration uses a local optimisation, it may fail on a small number of data sets. Therefore, prior to segmentation, the registration results should be checked. With the path and name of the data list entered into the relevant fields of the DODECANTS tool, press the "CC start" button. This will initiate the coregistration checker. Then press the "CC next button". This will load the standard brain, the first data set in the data list, and the registration result into the Coreg tool, and display them in the three Coreg Tv's. The result can be visually inspected. Pressing the "CC next" button again will

advance to the next data set in the data list. When the end of the data list is reached, terminate the coregistration checker by pressing the "CC end" button.

If the registration has failed on any of the data sets, (i.e. the data set is obviously not aligned to the standard brain), it can be repeated using the Coreg tool whilst still running the coregistration checker, by loading in the rough .tair file from the Coreg tool and performing some manual alignment followed by another attempt at automatic alignment. Remember to remove the previous .tair_mi file and write out the updated .tair file, using the naming convention described above, once a satisfactory alignment has been achieved. See the TINA User's Guide for further instructions on the use of the Coreg Tool.

22.3.3 Segmentation

The CSF segmentation consists of two stages: fitting a partial volume model to the histogram of the grey-levels in the image volume under analysis, and production of a volumetric map of the CSF in the images. The separation of the model fitting from the actual segmentation allows the models to be checked, and any fit failures corrected.

CSF segmentation is performed by optimising a model describing the pure tissue and partial volume contributions present in the images under analysis. The initial model must be prepared prior to segmentation, and its location specified in the DODECANTS tool interface. Instructions on how to prepare these models is given in the TINA User's Guide, in the chapter describing the segmentation algorithm. The model consists of parameters such as the number of pure tissues present in the images, their mean grey levels and standard deviations, and a-priori probabilities describing their frequency of occurrence in the images, and so each MR image type requires a different model. However, the parameters are stored in plain ASCII text files, allowing the initial model for the optimisation to be written manually, and some sample initial models are provided on the TINA website at

`http://www.tina-vision.net/tina-main/tarballs/segmentation/`

For example, the following initial model file was designed for use on T1 IRTSE images, and contains descriptions of three tissues (white matter, grey matter and CSF):

```
3 1 0.00000000000000000001
-1800 1.0
-650 1.0
-120 1.0
0.0067
0.0143
0.0167
200    600    0
600    3500   2000
0      2000   3500
CSF GM WM
```

The first line lists the number of tissues included in the model (3), the number of images to be segmented (1) and the threshold used to reject outlier pixels (small). The "number of images to be segmented" parameter allows multi-dimensional data to be used i.e. more than one MR image type of the same region. However, the DODECANTS tool assumes that only one image volume is available. The next three lines list the mean grey levels of the tissues in the model and the priors used to represent their frequency of occurrence in the images under analysis. The following three lines are the diagonal elements of the inverse covariance matrix (i.e. the inverse variances of the model components). The next three lines are a matrix describing the frequency of occurrence of partial volume voxels: zeros in the matrix imply that the two tissues have no common boundaries. The final line lists labels for each of the tissues in the model. See the chapter of the TINA User's Guide describing the segmentation algorithm for more details.

The DODECANTS tool assumes that only a single MR image volume is available for each subject under analysis, since this is the most common scenario in practice. It applies the TINA segmentation routine by concatenating the sequence into a single image, and applying the segmentation routine to both the image grey levels and the gradients, using the procedure described in TINA Memo no. 2005-013. It had been found empirically, particularly in young subjects with small CSF volumes, that the CSF peak in the image histogram can be obscured by partial volume contributions from voxels containing mixtures of CSF and other tissues. Therefore, the tool includes functionality to select sub-regions of the image volume containing large amounts of CSF, thus enhancing the CSF peak in the image histogram. This consists of selecting two rectangular sub regions from the images, and fitting only the data in those regions. This procedure has the additional benefits that less data is used in the segmentation, reducing the processor time required, and the selected region includes only grey matter, white matter and CSF, so only a three tissue model is required. However, this model must still be written to correspond to the MR image type being used.

The coordinates of these regions can be accessed by pressing the "Params" button in the EM Segmentation section of the tool. The parameters have been set up to select regions around the anterior and posterior limits of the ventricles, where the largest concentrations of CSF are found. Users may wish to experiment with these parameters. In order to display the selected regions, enter the pathname of the standard brain image volume into the "Image file" field of the Sequence tool, enter the number of slices (49) into the "End" field, and select "ANLZ" on the "File:" choice list. Then press "Load" to load the standard brain volume, which will be displayed in the Sequence tool Tv. Next, start the "Params" dialog box of the Coreg tool and enter -2500 into the "threshold" field. This will ensure that all voxels are displayed. Enter the mid-point of the standard brain sequence (128.0, 128.0, 24.5) into the "Center x", "y", and "z" fields of the Coreg tool, then press "zoom" and "anaglyph". The standard brain image will then be displayed in the three Coreg tool Tv's. Finally, press the "Display Box" button in the DODECANTS tool, followed by "init" in any of the three Coreg Tv's, to display the regions selected through the segmentation parameters dialog box. In order to clear the Coreg displays, press "Del Seq" in the Sequence tool, followed by "Image" in the Coreg tool. The parameters of the segmentation region selection can then be manipulated, and the above procedure repeated to display the altered regions.

The limitations of image display in TINA dictate that the images prepared by the "Display box" procedure are padded with zeros to keep their dimensions consistent with those of the original sequence. This produces a large peak at zero in the histogram, and so will affect the model fitting stage of the segmentation. Therefore, an alternative procedure must be applied to display the histogram of the selected region and to set up the initial model. Prepare a data list with only one data set listed, and enter the location into the DODECANTS tool as usual. Then press the "Image prep" button. This will prepare a set of non-padded sub-images in the Sequence tool. Press the "Seq− >one" button in the DODECANTS tool to concatenate these into a single image. Press the "push" button to copy this image to the Imcalc stack, and press "init" in the Imcalc TV to display the image. A histogram can then be produced by selecting "hist" in the "Imcalc mice" choice list, then clicking and dragging in the Imcalc TV. The histogram of the selected region will be displayed in the Imcalc graph Tv. This will allow parameters such as the mean grey levels of each tissue to be determined. Then a rough guess at the initial model can be generated, based on the example given above. In order to display the result, press the "NMR Segment" button in the top-level tinaTool window to launch the segmentation tool. Then enter the pathname of the model file into the "Model File" field, and press input. Finally, press "1D hist" in the NMR Segment tool to display the model components, overlaid on the image histogram, in the Imcalc Graph Tv. The initial model can then be refined and redisplayed manually, until a satisfactory initial model is generated. In addition, pressing the "hfit scale" button in the NMR Segment tool will scale the model parameters to improve the fit to the histogram. Combinations of these operations should be used to produce a satisfactory initial model. Once this has been done, enter the desired pathname for the finalised initial model into the "Model file" field of the NMR Segment tool and press "Output" to save it to disk.

In order to run the segmentation stage of the algorithm, start a tinaTool as described above, and enter the directory and filename of the data list into the relevant fields. Then enter the filename of the initial tissue model into the "initial model" field. The tool assumes that the initial model will be in the same directory as the data list. Enter any required parameters into the segmentation parameters dialog box. Start the model optimisation stage by pressing the "EM: model" button. The tool will then run through each data set in the list, fitting the initial model to the data, and output the optimised models to text

files in the same directories as the data sets. For example, if the data set is called alice.img, the optimised model will be called result alice_EM_model.

In order to facilitate the identification of any model fitting failures, the "Model output" button will run through a data list, writing out the means and standard deviations of the pure tissue components of the models to a single text file, using the name given in the "Output files" field of the DODECANTS tool. What to do about failed models is up to the user: for example, segmentation could be repeated using a different definition of the regions, or a different initial model, or a mean of all of the successfully fitted models could be produced and substituted for the failed models.

Once the optimised models have been checked, press the "EM: seg" button to run through the data list, picking up the optimised models, and segmenting the CSF. The algorithm assumes that the binary masks required for detection of the eyes and sinuses from the CSF maps will be contained in an ANALYZE file, located in the same directory as the standard brain image volume, with the filename name_bincut where name is the filename of the standard brain image volume. The CSF maps will be multiplied with these masks, and output as ANALYZE files alongside the original data sets. For example, if the original data set is called alice.img, the masked CSF maps will be called alice_masked_EM.img. These images can be loaded into the Sequence tool for visual inspection: see the chapter of the TINA User's Guide on the Sequence tool for further information.

22.3.4 CSF Volume Measurement

Once the registration and segmentation stages have been completed, the CSF volumes can be counted. Enter the directory and filename of the data list into the relevant fields. Then choose a filename for the output file that will contain the volume measurements, and enter it into the ".tvs file" field. Ensure that the filename has the .tvs extension. Finally, press the "Bulk CSF count" button. The tool will then run through the data sets listed in the data list, measuring the CSF volumes and some other important parameters, and output them to the .tvs file. This file will contain records that look like the following:

```
alice 1 66.000000
--a 9961.531250 --b 16343.578125  --c 9568.833008
-+a 9009.450195 -+b 19038.785156  -+c 7531.889160
+-a 9074.429688 +-b 18499.179688  +-c 9097.030273
++a 11967.398438 ++b 23259.582031  ++c 10958.814453
x mm 122.108154, y mm 103.918434, z mm 157.280487
```

The first line consists of the label, disease code and age as given in the data list. The next four lines give the CSF volume measurements in each of the twelve sectors of the CSF space (in mm^3), in the order:

anterior left superior	central left superior	posterior left superior
anterior left inferior	central left inferior	posterior left inferior
anterior right superior	central right superior	posterior right superior
anterior right inferior	central right inferior	posterior right inferior

Left and right here refer to the image plane as seen on the monitor, not necessarily to the left and right hands of the subject. The final three lines give the maximal extents of the CSF space in the x (lateral), y (anterior-posterior) and z (inferior-superior) directions.

Many users will be interested only in overall CSF volume, and so the .tvs file can be rewritten into a format more suitable for reading into other analysis programs. Enter the directory and filename of the .tvs file into the "directory" and ".tvs file" fields of the DODECANTS tool and enter the desired output file name into the "Output files" field. Then enter the number of records in the .tvs file (i.e. the number of data sets listed in the original data list) into the "KNEAR" field, and press the "TVS$-$ >V12 button. The .tvs file will be rewritten into columns in the output file, in the following order:

```
subject disease code
```

```
subject age
--a volume (normalised for head size)
--b volume (normalised for head size)
--c volume (normalised for head size)
-+a volume (normalised for head size)
-+b volume (normalised for head size)
-+c volume (normalised for head size)
+-a volume (normalised for head size)
+-b volume (normalised for head size)
+-c volume (normalised for head size)
++a volume (normalised for head size)
++b volume (normalised for head size)
++c volume (normalised for head size)
total CSF volume (not normalised)
CSF bounding box volume
total intracranial volume
total CSF volume (normalised)
```

The head size normalisation is accomplished through division by the total intracranial volume (TIV) [9]. The total intracranial volume is calculated from the volume of the bounding box on the CSF space, as described in TINA Memo no. 2004-002. Thus, all measurements are quoted as proportions of the TIV.

22.3.5 KNN Classifier

The final stage of the algorithm is the normalisation for age-related atrophy and the KNN classifier. The classifier can also be run just to get the age-normalised CSF volumes: in fact, it is anticipated that this will be the approach adopted by most users.

In order to run the classifier, enter the directory name and filename of the .tvs file, and a name for output files, into the relevant fields of the DODECANTS tool. Then enter the number of records in the .tvs file into the "KNEAR field". Finally, press the "Run classifier" button. The tool will then run through the .tvs file, picking up the CSF volume measurements, normalise them for head size and age related atrophy (see TINA Memo no. 2004-002), compute the reduced variables W1 to W5 (see above), and run a leave-one-out KNN classifier on the resultant data.

There are two switches to control aspects of this process. The normalisation for age related atrophy corrects the CSF volumes by multiplying by the ratio of the average CSF volume in normal subjects at a standard age to the average CSF volume in normal subjects at the subject's age. This standard age is calculated from the mean of the ages of the subjects listed in the .tvs file. However, the "Fix mean age" switch allows this mean age to be entered manually. This allows the mean age to be fixed across analyses of multiple data lists, allowing the results to be compared directly. The second switch, "Optimise scales", dictates whether to optimise the kernel size used in the KNN classifier to give the best diagnosis, or to use those determined in our own work (see [8]).

The output will be contained in five files, called name, name.v12_norm, name.dat, name.colours and name.row, where name is the filename entered in the "Output files" field. The name.dat file contains rows of the variables W1 to W5 (see above), followed by the subjects age, for each subject. The name file contains a list of the patients, with their disease code as specified in the original data list and the most likely disease code as determined by the nearest neighbour classifier. This is followed by two matrices. The first is a matrix of original diagnosis (columns) against the predicted diagnosis (rows). The second is the same matrix, but summed over the probabilities associated with the predicted diagnoses, rather than quantised to the most likely diagnosis. Note that both matrices have rows and columns for the non-existent disease code 0. The name.v12_norm file has the same entries as the .v12 file described above, but with the volume measurements normalised for age-related atrophy. The name.row file lists the subject labels from the original data list, and the name.colours file lists colours associated with each disease code. These last two files are used for data display in xgobi: if the name.dat file is loaded into this program, then the points will automatically be labelled and coloured using these files.

22.4 Quick Reference

- Directory: the path to the directory (absolute or relative) in which the data list is located.
- Data list: the name of the data list.
- Initial model: the path (absolute or relative) of the initial model file used in the segmentation.
- .tvs file: The path (absolute or relative) of the file which will be used to output the volume measurements.
- Output files: The path of various output files: this has multiple uses within the tool.

1. MI Coregistration:
   - Bulk coreg: Starts coregistration of the data sets listed in the data list
   - CC start: Starts the coregistration checker (visual inspection of the registration result).
   - CC next: Advances the coregistration checker to the next data set in the data list.
   - CC end: Ends the coregistration checker.
2. EM segmentation:
   - Params: Launches the dialog box containing the segmentation parameters.
   - Display Box: Displays the region specified by the segmentation parameters on the standard brain data set.
   - Image Prep: Takes the currently loaded sequence and replaces it with the sub-regions defined in the params dialog box, placing the result in the sequence tool.
   - Seq− >one: Takes the currently loaded sequence and concatenates it into a single image, placing the result in the sequence tool.
   - One− >seq: Reverses the effect of the Seq− >one button.
   - EM model: Starts the model optimisation stage of the segmentation.
   - EM seg: Starts the segmentation based on the optimised models.
   - Model Output: Outputs the main parameters of the optimised models to the file specified in the “Output files” field.
   - Bulk CSF count: Counts the CSF volumes and outputs them to the .tvs file specified in the “.tvs file” field.
   - TVS− >V12: Reads in a .tvs file and rewrites it into columns, for ease of loading into other analysis programs.
3. KNN Classifier:
   - Scales: launches the dialog box containing the scale parameters for the nearest neighbour classifier.
   - Fix mean Age: “On” fixes the mean age used in age-related atrophy normalisation to the age specified in the “mean age” field: “Off” calculates it from the mean age of the subject group.
   - Mean age: see above
   - Optimise scales: “On” will optimise the kernel sizes used in the KNN classifier: “Off” will use those specified in the code.
   - KNEAR: The number of subjects listed in the .tvs file.
   - Run Classifier: Reads in the .tvs file specified in the “.tvs” file field and runs the KNN classifier in a leave-one-out fashion. A set of output files are generated with various extensions appended to the pathname given in the “Output files” field. These files are designed to be displayed using xgobi. However, other display programs could be used.

Bibliography

[1] P A Bromiley, M Pokric, and N A Thacker. Computing covariances for mutual information coregistration. In *Proceedings MIUA'04*, pages 77–80, 2004.

[2] P A Bromiley, M Pokric, and N A Thacker. Emprical evaluation of covariance estimates for mutual information coregistration. In *Proceedings MICCAI'04*, pages 607–614, 2004.

[3] P A Bromiley, N A Thacker, and A Jackson. Trends in brain volume change with normal ageing. In *Proceedings MIUA'05*, pages XX–XX, 2005.

[4] M Pokric, N A Thacker, M L J Scott, and A Jackson. Tina memo 2001-009: Multi-dimensional medical image segmentation with partial voluming. Technical report, Imaging Science and Biomedical Engineering Division, University of Manchester, 2001.

[5] N A Thacker. Tina memo 2004-006: Parameter estimation for em mixture modelling and its relationship to likelihood and eml. Technical report, Imaging Science and Biomedical Engineering Division, University of Manchester, 2004.

[6] N A Thacker, M Pokric, and A J Lacey. Tina memo 2004-009: Model selection and convergence of the em algorithm. Technical report, Imaging Science and Biomedical Engineering Division, University of Manchester, 2001.

[7] N A Thacker, M Pokric, and D C Williamson. Tina memo 2004-009: Multi-dimensional medical image segmentation with partial voluming and gradient estimation. Technical report, Imaging Science and Biomedical Engineering Division, University of Manchester, 2004.

[8] N A Thacker, A R Varma, D Bathgate, S Stivaros, J S Snowden, D Neary, and A Jackson. Dementing disorders: Volumetric measurement of cerebrospinal fluid to distinguish normal from pathological findings; feasibility study. *Radiology*, 224(1):278–285, 2002.

[9] J L Whitwell, W R Crum, H C Watt, and N C Fox. Normalisation of cerebral volumes by use of intracranial volume: Implications for longitudinal quantitative mr imaging. *American Journal of Neuroradiology*, 22:1483–1489, 2001.

[10] D . Williamson, N . Thacker, S R Williams, and M Pokric. Tina memo 2002-006: Partial volume tissue segmentation using grey-level gradient. Technical report, Imaging Science and Biomedical Engineering Division, University of Manchester, 2002.

Chapter 23

Talairach Tool

23.1 Introduction

The Talairach Tool provides an interface to the Talairach stereotaxic atlas (Talairach & Tourneaux, 1988), commonly used in fMRI to label regions of the brain.

23.2 Tool Description and Use

23.2.1 Prerequisites to using the Altas

In order to sucessfully use the atlas, a sequence of images should be present in the Sequence Tool. In addition, these images should either have been co-registered to the standard Talairach brain, or the tair file containing the co-registration parameters should be loaded into the Coreg Tool.

The gzipped tar file “Taltar.gz” (available from the TINA tarballs repository at www.tina-vision.net/tarballs) should be unzipped and expanded (using tar zxvf Taltar.gz under linux/unix). A sensible location to do this is in /usr/local. The file expands into a directory called Taliarach [sic].

In the Talairach Tool, the “Talairach Atlas” field should contain the full pathname of the directory containing the Talairach atlas, plus an X for the beginning of the filename (eg, /usr/local/Taliarach/X).

23.2.2 Obtaining Atlas Descriptions for Single Pixels

Using the Sequence Tool, scroll through to the slice of interest. Click on the “Sequence Pick:” “taliarach” button on the Talairach Tool and then left click on the Sequence Tool Tv. The x,y,z-position in the Talairach Atlas are displayed in the “x coord”, “y coord” and “z coord” fields on the Tool, and a position label (giving 5 descriptions from coarse to fine, eg lobar to Brodmann area) is given in the text window of the Tina Tool. See section 23.2.4 for a complete list of the regions subdivided into the 5 levels of description.

23.2.3 fMRI functionality

The buttons (Search Parameters, GO!, Activations, Grey Matter) at the base of the tool implement a limited fMRI-related functionality.

Search Parameters

Press this button to bring up a dialog box for entering x, y and z positional variance parameters on the x y and z coordinates. At present, this is redundant.

GO!

Press this button to obtain the atlas description at the co-ordinates specified in the x, y and z co-ordinate fields.

Activations

Press this button to produce a list of co-ordinates (in Talairach space) in the Tina Tool text window of the pixels in the image sequence which are above the threshold value, specified by the “Threshold” field in the Talairach Tool.

Grey Matter

Press this button to produce, in the Imcalc, an image of the grey matter pixels (as defined by the atlas) which are above the threshold (specified by the “Threshold” field) in the currently displayed image in the Sequence Tool.

Press to Finish

Press this button to delete from memory any loaded database files.

23.2.4 Talairach Regions

```
0 No data

Label 1

1 Inter-Hemispheric
2 Left Cerebrum
3 Right Cerebrum
4 Right Cerebellum
5 Right Brainstem
6 Left Brainstem
7 Left Cerebellum

Label 2

8 Posterior Lobe
9 Anterior Lobe
10 Frontal-Temporal Space
11 Limbic Lobe
12 Medulla
13 Pons
14 Midbrain
15 Sub-lobar
16 Occipital Lobe
17 Temporal Lobe
18 Parietal Lobe
19 Frontal Lobe

Label 3

20 Posterior Cingulate
```

21 Anterior Cingulate
22 Subcallosal Gyrus
23 Sub-Gyral
24 Transverse Temporal Gyrus
25 Uncus
26 Rectal Gyrus
27 Fusiform Gyrus
28 Inferior Occipital Gyrus
29 Inferior Temporal Gyrus
30 Insula
31 Parahippocampal Gyrus
32 Lingual Gyrus
33 Middle Occipital Gyrus
34 Orbital Gyrus
35 Middle Temporal Gyrus
36 Superior Temporal Gyrus
37 Superior Occipital Gyrus
38 Precentral Gyrus
39 Inferior Frontal Gyrus
40 Cuneus
41 Angular Gyrus
42 Supramarginal Gyrus
43 Cingulate Gyrus
44 Inferior Parietal Lobule
45 Precuneus
46 Superior Parietal Lobule
47 Middle Frontal Gyrus
48 Paracentral Lobule
49 Postcentral Gyrus
50 Precentral Gyrus
51 Superior Frontal Gyrus
52 Medial Frontal Gyrus
53 Uvula of Vermis
54 Pyramis of Vermis
55 Tuber of Vermis
56 Declive of Vermis
57 Culmen of Vermis
58 Cerebellar Tonsil
59 Inferior Semi-Lunar Lobule
60 Fastigium
61 Nodule
62 Uvula
63 Pyramis
64 Tuber
65 Declive
66 Culmen
67 Cerebellar Lingual
68 Hippocampus
69 Extra-Nuclear
70 Lentiform Nucleus
71 Amygdala
72 Hypothalamus
73 Red Nucleus
74 Substania Nigra
75 Claustrum
76 Thalamus

77 Caudate
78 Cerebro-Spinal Fluid

Label 4

79 Gray Matter
80 White Matter

Label 5

81 Brodmann area 1
82 Brodmann area 2
83 Brodmann area 3
84 Brodmann area 4
85 Brodmann area 5
86 Brodmann area 6
87 Brodmann area 7
88 Brodmann area 8
89 Brodmann area 9
90 Brodmann area 10
91 Brodmann area 11
92 Brodmann area 12
93 Brodmann area 13
94 Brodmann area 17
95 Brodmann area 18
96 Brodmann area 19
97 Brodmann area 20
98 Brodmann area 21
99 Brodmann area 22
100 Brodmann area 23
101 Brodmann area 24
102 Brodmann area 25
103 Brodmann area 27
104 Brodmann area 28
105 Brodmann area 29
106 Brodmann area 30
107 Brodmann area 31
108 Brodmann area 32
109 Brodmann area 33
110 Brodmann area 34
111 Brodmann area 35
112 Brodmann area 36
113 Brodmann area 37
114 Brodmann area 38
115 Brodmann area 39
116 Brodmann area 40
117 Brodmann area 41
118 Brodmann area 42
119 Brodmann area 43
120 Brodmann area 44
121 Brodmann area 45
122 Brodmann area 46
123 Brodmann area 47
124 Caudate Tail
125 Caudate Body
126 Caudate Head

127 Dentate
128 Ventral Anterior Nucleus
129 Ventral Posterior Medial Nucleus
130 Ventral Posterior Lateral Nucleus
131 Medial Dorsal Nucleus
132 Lateral Dorsal Nucleus
133 Pulvinar
134 Lateral Posterior Nucleus
135 Ventral Lateral Nucleus
136 Midline Nucleus
137 Anterior Nucleus
138 Mammillary Body
139 Fourth Ventricle
140 Optic Tract
141 Anterior Commissure
142 Corpus Callosum
143 Third Ventricle
144 Medial Globus Pallidus
145 Lateral Globus Pallidus
146 Nucleus Accumbens Septi
147 Medial Geniculum Body
148 Lateral Geniculum Body
149 Subthalamic Nucleus
150 Lateral Ventricle
151 Putamen

152 No Data

Chapter 24

Flow Tool

24.1 Introduction

The Flow Tool provides functionality for post-processing the resultant images of the "Perfusion" analysis of the MR Analysis Tool. It estimates the absolute fractional Cerebral Blood Volume (CBV) from the raw CBV maps, and derives the Net Mean Transit Time maps (NMTT) from the Time to Mean (TTM) maps. The Net Cerebral Blood Flow (NCBF) is calculated from these maps. Details of the algorithms are described in Tina Memo 2002-001.

The Tool is divided into three sections. First is the initialisation of the image volumes, next is the actual calculations on the images to obtain NCBF values, and finally there are functions for producing histograms of regional NCBF, based on the Talairach Atlas.

24.2 Pre-requisites for Use

Before using the Flow Tool, sequences of CBV and TTM maps need to be created using the MR Analysis Tool. These sequences should contain the same number of images, and be of corresponding slices. These sequences should ideally contain at least 5 contiguous slices, a minumum of three is required.

To use the Regional histogram functionality, the Coreg Tool needs to be open, and either the co-registration of the CBV/TTM maps to the Talairach atlas brain needs to be performed, or the tair file containing the registration parameters should be opened in the Coreg Tool. Also, the Talairach Tool, specifying the location of the Talairach atlas database files needs to be open.

24.3 Using the Tool

24.3.1 Initialise Image Volumes

Load the CBV images into the Sequence Tool. Please ensure that the x, y and z scales in the Sequence Tool are as they were in the original data from which they were estimated (if they are not, insert them manually, and click on "First"). Press the "CBV volume" button on the Flow Tool to ensure the sequence is held in memory. Next, load the TTM images into the Sequence Tool. Please ensure the TTM sequence has the same number (and same correspondence) of images as the CBV sequence, and ensure that the x, y and z scales are correct. Press the "TTM volume" button to hold these images in memory.

24.3.2 Perform Flow Calculations

The flow calculation buttons are intended to be pressed in order, as indicated by the arrows linking them on the tool. The calculations are not all concatenated into one button in order that the user can save the

output images of the various processing stages. However, please do not press the buttons out of order.

Process CBV

Press this button to normalise the CBV values of the entire volume to the value of a voxel which is 100% blood. In order to calculate this value, a maximum intensity projection (MIP) image of the entire volume is created. The square-root image of this undergoes tangential smoothing to remove potential fit-failures (please see Tina Memo 2002-001 for more details) and this image is displayed on the Imcalc Tool Tv. The square-root of the value of the 100% voxel is displayed in the Tina Tool text window. This value should be somewhere in the region of 1000-2000. If it is very much greater than that, check through the CBV volume and delete any high-valued pixels which are fit failures.

Process TTM

Next press this button to produce the NMTT images. These are displayed in the Sequence Tool Tv (press init on the Tv if they fail to appear). Note that there are two fewer images in this sequence than in the TTM sequence, as no NMTT image can be calculated for the upper and lowermost images in the sequence (as slices above and below the slice of interest are required for calculation of the NMTT). If you wish to output these images, it is a good idea to set the start and end values on the Sequence Tool to the correct values.

NMTT Masks

The NMTT images contain high, spurious values at the interface between the background and brain. Pressing this button produces masks to remove these high values. The binary masks are displayed in the Sequence Tool Tv, press init on the Tv if they fail to appear. Note that as for the NMTT maps, there are two fewer images in the sequence than in the original TTM sequence.

Get Flow Vol

The Net Cerebral Blood Flow (NCBF) values are computed from the CBV and masked NMTT maps. The NCBF is in units of millilitres of blood per 100g tissue per minute. In order to produce maps with pixel value distributions consistent with the Gaussian distribution, the images undergo a logarithmic transformation, and these images are displayed in the Sequence Tool Tv.

24.3.3 Regional Histograms

As explained above, before running the histogram functions to obtain regional log flow estimates, it is necessary to either co-register the flow volume to the Talairach atlas brain, or to load in a tair file containing the registration parameters into the Coreg tool. In order to see the plots of the histograms, an Imcalc Graph Tv needs to be opened.

Init Hist.

Pressing this button creates the histograms into which the log(NCBF) values will be placed. Three sets (for the whole brain and left and right hemispheres) of 153 histograms are created, covering gross brain regions, lobes, sub-lobar structures and whether the voxel is in grey or white matter. Please see section 23.2.4 in the description of the Talairach Tool for a list of the regions.

Calc Flow Hists

Pressing this button allocates the log(NCBF) values for the entire sequence to the relevant regional histograms. Note that the histograms are not mutually exclusive; one voxel value will be entered into

more than one histogram because the regional labels are hierarchical.

Plot Hist

Having created the histograms, use the “Hist” selection to choose whether to display a histgram for the whole, left or right of the brain. Insert the number of the histogram (see section 23.2.4) into the “Hist no:” field, then press the “Plot Hist” button. A histogram ofthe region is displayed in the Imcalc Graph TV, and the region description label and mean of the values in the histogram is displayed in the Tina Tool text window.

Chapter 25

Cortical Thickness Tool

25.1 Introduction

This tool enables the estimation of region-specific cerebral cortical grey matter thickness from T1 weighted inversion recovery MR images. The tool currently relies on the assumption that CSF has on average a lower image intensity than grey matter, which has a lower intensity than white matter.

25.2 Tool Description

This section describes the buttons and parameters of the tool, please see the next section for a description of how to use the tool.

25.2.1 Cortical Thickness Tool

- **Tv: 3D rep** Use this button to initialise a TvTool as a 3D veiwer. This is required for drawing **3D lines** (see below).
- **Mouse: seq** Hold down this button and select the `Profile` option. Left click on the Sequence Tv at a GM/WM interface (which you should already have determined using the **Get GM/WM** button). If the position you have chosen coincides with the boundary already determined, the program calculates the direction of the surface normal through the grey matter and the point along this line at which an opposing GM edge is found. If an Imcalc Graph Tv is open, a profile plot of grey matter probability along this line is plotted. If an Imcalc Tv and Imcalc2 Tv are open, 2D interpolated sections of pixel values, extending 2 pixels either side of the line are plotted, where if the line is considered to move in the z direction, then the planes represented are x-by-z and y-by-z.
- **Cort params** Brings up the parameters dialogue box
- **Help** At present this does not work.
- **Test Boundary** Use this to view the results of the GM/WM boundary detection. Install a Mono Tv, push the slice of interest from the Sequence Tool into the Imcalc Tool and pop it into the Mono Tool. Press the Test Boundary button; the results should appear in pink overlaid on the Mono Tv. Adjust the Canny parameters and Grey/White Midpoint (see below) as necessary.
- **Cortical Thickness Algorithm:**
  - **Get GM vol** In order to run the cortical thickness algorithm, it is necessary to load in both a segmented grey matter volume and the original T1 images. Pressing this button stores the currently loaded sequence (which should be the GM segmented volume) in memory so that the T1 volume can be loaded without losing the segmented volume.

- **Remove Skull** Install an Imcalc Tv, then press this button. This produces a mask for each slice which excludes the CSF/skull boundary in the GM/WM boundary detection part of the algorithm, as CSF/skull partial volumes have similar intensity values to grey matter. In practice, this is not really necessary because the Talairach atlas regions will tend to exclude the CSF/skull boundary, so any erroneous thicknesses due to this boundary will be ignored. If you do use it, you need to watch the images which appear in the Imcalc Tv. These show (flashing) views of each slice of the brain. It is assumed that there is a continuous band of grey matter encompassing the rest of the brain, and you should see the images fill with red, but the brain should remain black/purple. If any part (particularly around the occipital lobe) does not have a continuous band of GM, and part of the brain fills with red, you will need to increase the **Skull Seg** parameter value in the parameters dialog box, perhaps in increments of 0.05.
- **Get GM/WM** Pressing this button results in the GM/WM boundary being determined for all slices of the brain.
- **Get Thickness** Takes the GM/WM boundary, determines the 3D surface normal at each voxel on the boundary, traverses the GM segmented volume along this direction up to the **search extent** defined in the parameters dialogue box. If a true edge or a dip is found, the distance traversed to that edge/dip is inserted into a histogram representing the region it is in, according to the Talairach atlas (Please see Tina Memo 2004-007 for more details). In order for this to work, you also need to open the Coreg Tool and specify and input an Air File for the registration parameters to register the image volumes to the Talairach Atlas. In addition, you need to open the Talairach Tool and specify the location of the Talairach atlas, as well as the start of the filenames (the files all start with an X, so you need to specify `/pathname/`X, eg, /usr/local/Talairach/X).
- **Apply Median Filter** This button removes the effect of any spuriously long thickness values, by taking each voxel on the GM/WM boundary, and comparing the thickness value associated with it with the median value of a window of 5 boundary voxels (with the voxel of interest at the central position). If the difference is greater than the **Edge Limit** parameter (in the parameters dialogue box, default of 4mm), then the value of the voxel under consideration is set to the median. In practice this produces very few false positives and false negatives, but does remove spurious results.

• **Plot Regional Thickness Histograms**

- **Hist: Whole/Left/Right** Choose whether you want to view the histogram of the given structure (defined by **Hist. No.** as described below) for the left hemisphere, right hemisphere or both (whole) hemispheres.
- **Hist no:** Choose the number of the histogram you wish to view. (See section on Histogram numbers and regions defined for a list).
- **Single Hist.** If an Imcalc Tv Graph is installed, the histogram of the thickness values in a region is displayed, and summary statistics, comprising the median, number of entries in the histogram, lower quartile and upper quartile, are shown in the tinaTool panel.
- **All Hists.** Plots the histograms of all the regions, but more importantly writes the summary statistics of all the regions to the tinaTool panel so they can be copied and pasted to other applications.
- **Remove Hists.** Removes the histograms from memory.

• **Graphics Results** This allows 2D and 3D visualisation of the cortical thickness results.

- **2D Overlay** For the current slice in the sequence tool, displays a 2D overlay of the lines through the GM starting at the boundary defined in this image. Please ensure you set the Sequence Tv as the current Tv, otherwise the overlay will appear on a different TvTool. Note that this is a 2D representation of 3D volume, hence there will be some lines which do not appear to end at valid boundaries. These simply end at out of plane positions. If you wish to interrogate these lines further, please use the Mouse Seq Profile button described above.

  - **3D Lines** For all voxels on the GM/WM boundary, plots a 3D visualisation of all the lines through the GM. Please ensure the "threed" TvTool is the current Tv. This can be installed using the **Tv:3D rep** button described above.

- **GM/WM Boundary Placement Checks** It is possible to calculate the difference in grey level between the assumed **GM/WM midpoint** value and a bootstrapped estimate of the true boundary value if it is assumed that the voxel values either side of the boundary (using the 3D surface normal direction to the boundary) by some distance defined using the **extent** parameter in the dialogue box are in pure tissue. The average of the intensity values at these positions, minus the GM/WM midpoint values gives the offset values.
  - **Calculate Offset** Calculates the offset as described above, and inserts them into histograms according to region (so, again, requires that an air file registering the dataset to the Talaiarch Atlas has been input in the Coreg tool and that the location of the Talairach lut files has been defined in the Talairach tool).
  - **Plot Single Offset** Plots the histogram of offset values for a region in the Imcalc Graph Tv and writes the offset value of the peak of the histogram to the tinaTool panel.
  - **Plot All Offsets** Plots histograms of offset values for all regions in the Imcalc Graph Tv and writes peak offset values for all regions to the tinaTool panel.

25.2.2 Cortical Thickness Parameters

- **Canny Parameters** The canny parameters given here are the same as in the Edge Tool. Hysteresis thresholding is part of the Canny algorithm, whereby first the edges with an intensity gradient above a low threshold are identified; then linked edge strings that are entirely below an upper threshold are eliminated.
  - **Sigma** The parameter of the gaussian convolution profile used to smooth the image prior to the gradient calculation. It essentially selects the scale of the edge detector.
  - **Precision** The ratio of the smallest stored value of the gaussian convolution profile with respect to the largest. Lower values give increased precision, larger convolution masks and increased computation time.
  - **Lower Thresh** The lower threshold (see above).
  - **Upper Thresh** The upper threshold (see above).
  - **Length Thresh** A lower threshold on the length of connected edge strings, where strings shorter than this value are eliminated.
- **Skull Segmentation Parameters**
  - **Skull Seg** The skull segmentation process involves a couple of erosion/dilation operations, the first of which requires user-defined thresholding to ensure that the white matter of the brain is not removed too. This value should be between 3.0 and 4.0, if it is set outside this range, the value used will be 3.05.
- **Cortical Thickness Parameters**
  - **Grey/White Midpoint** The average of the mean tissue values for the grey and white matter.
  - **Grey Mean** The grey matter mean tissue value.
  - **Search extent** The maximum search extent when finding the opposing grey/white matter boundary. The maximum search extent for finding a grey matter/CSF boundary is half of this. The default value is 20mm. This parameter is also used when performing the bootstrapped grey matter offset calculation, when a value of approximately 2mm is sufficient.
  - **Edge limit** When applying the Median Filter to the thickness values at each position on the GM/WM boundary edge string, this value is the maximum difference allowed between the central edge position value and the median of the five currently being windowed.
  - **Fraction** The fractional volume on the GM volume probability maps at which a transition from GM to another tissue takes place. It is assumed to be 0.5.

25.3 Standard Usage

This section is intended to provide a step-by-step description of how to obtain regional cerebral cortical thickness estimates using the cortical thickness tool. It is however assumed that the user has a copy of the Talairach standard brain and the Talairach look up tables; has already registered the T1 volume to the Talairach standard brain and has saved the air file containing the registration parameters.

- Open six TvTools. Open the Coreg, Sequence, Imcalc, Cort. Thick., and Talairach Tools. Install the TvTools as `z coreg`, `y coreg`, `x coreg`, `sequence`, `imcalc` and `graph`.
- Set the sequence tool start and end values to 0 and 38, set the file type to AIFF, set the Image File name to /`your_pathname`/Rbrain## (where your_pathname is the path of the directory containing the Talairach brain). Load the sequence.
- On the coreg tool, press the Latch image button. Set the Centre x, y, z and factor parameters to 32, 32, 18.5 and 0.25 respectively. Click on the Coreg tool zoom button.
- On the Coreg Tool, press the Reslice linear button, then press the Latch Anaglyph button.
- Insert the full pathname to your air file containing the registration parameters of your T1 volume to the Talairach brain in the Air File field.
- Next load your grey matter segmentation volume into the sequence tool, making sure the start, end, and file type parameters are correct. Press the Load button.Once the volume is loaded, ensure that the x, y and z parameters are correct. If they are not, insert them, and click on the "first" button to get them into memory.
- Press the Get GM Vol button in the Cortical Thickness tool.
- Next, load the T1 sequence, making sure the start, end and file type parameters are correct. Please also make sure that the T1 volume and probability volume are the same dimensions and have the same number of slices. Press the Load button. Once the volume is loaded, please ensure the x, y and z parameters are correct, and adjust them if not.
- Press the Coreg input button.
- Press the Cort params button on the Cortical Thickness tool to open the Cortical Thickness Parameters dialogue box. Set the grey/white matter midpoint and grey mean parameters to the values you have previously obtained.
- In the Talairach Tool, set the Talairach Atlas field to the path of the directory containing the atlas look up tables. Also (and this is most important) add the X part of the filenames to the path. eg, if the files are in /usr/local/Talairach, you would insert /usr/local/Talairach/X.
- If you want to perform the "skull stripping" press the Remove Skull button on the Cortical Thickness Tool.
- Next, press the Get GM/WM button, followed by the Get Thickness button, then the Apply Median Filter button. The median cortical thickness in each region has now been estimated.
- To output the median thickness, number of entries, lower and upper quartile ranges of the regional histograms (both for the whole brain, left hemisphere and right hemisphere), press the All Hists. button.
- Individual histograms can be viewed by inserting the number of the histogram you wish to view (see next section for numbers and labels) and clicking on Single Hist.

25.4 Histogram Numbers and Labels

```
0 No data
1 Inter-Hemispheric
2 Left Cerebrum
```

3 Right Cerebrum
4 Right Cerebellum
5 Right Brainstem
6 Left Brainstem
7 Left Cerebellum
8 Posterior Lobe
9 Anterior Lobe
10 Frontal-Temporal Space
11 Limbic Lobe
12 Medulla
13 Pons
14 Midbrain
15 Sub-lobar
16 Occipital Lobe
17 Temporal Lobe
18 Parietal Lobe
19 Frontal Lobe
20 Posterior Cingulate
21 Anterior Cingulate
22 Subcallosal Gyrus
23 Sub-Gyral
24 Transverse Temporal Gyrus
25 Uncus
26 Rectal Gyrus
27 Fusiform Gyrus
28 Inferior Occipital Gyrus
29 Inferior Temporal Gyrus
30 Insula
31 Parahippocampal Gyrus
32 Lingual Gyrus
33 Middle Occipital Gyrus
34 Orbital Gyrus
35 Middle Temporal Gyrus
36 Superior Temporal Gyrus
37 Superior Occipital Gyrus
38 Precentral Gyrus
39 Inferior Frontal Gyrus
40 Cuneus
41 Angular Gyrus
42 Supramarginal Gyrus
43 Cingulate Gyrus
44 Inferior Parietal Lobule
45 Precuneus
46 Superior Parietal Lobule
47 Middle Frontal Gyrus
48 Paracentral Lobule
49 Postcentral Gyrus
50 Precentral Gyrus
51 Superior Frontal Gyrus
52 Medial Frontal Gyrus
53 Uvula of Vermis
54 Pyramis of Vermis
55 Tuber of Vermis
56 Declive of Vermis
57 Culmen of Vermis
58 Cerebellar Tonsil

59 Inferior Semi-Lunar Lobule
60 Fastigium
61 Nodule
62 Uvula
63 Pyramis
64 Tuber
65 Declive
66 Culmen
67 Cerebellar Lingual
68 Hippocampus
69 Extra-Nuclear
70 Lentiform Nucleus
71 Amygdala
72 Hypothalamus
73 Red Nucleus
74 Substania Nigra
75 Claustrum
76 Thalamus
77 Caudate
78 Cerebro-Spinal Fluid
79 Gray Matter
80 White Matter
81 Brodmann area_1
82 Brodmann area_2
83 Brodmann area_3
84 Brodmann area_4
85 Brodmann area_5
86 Brodmann area_6
87 Brodmann area_7
88 Brodmann area_8
89 Brodmann area_9
90 Brodmann area_10
91 Brodmann area_11
92 Brodmann area_12
93 Brodmann area_13
94 Brodmann area_17
95 Brodmann area_18
96 Brodmann area_19
97 Brodmann area_20
98 Brodmann area_21
99 Brodmann area_22
100 Brodmann area_23
101 Brodmann area_24
102 Brodmann area_25
103 Brodmann area_27
104 Brodmann area_28
105 Brodmann area_29
106 Brodmann area_30
107 Brodmann area_31
108 Brodmann area_32
109 Brodmann area_33
110 Brodmann area_34
111 Brodmann area_35
112 Brodmann area_36
113 Brodmann area_37
114 Brodmann area_38

115 Brodmann area_39
116 Brodmann area_40
117 Brodmann area_41
118 Brodmann area_42
119 Brodmann area_43
120 Brodmann area_44
121 Brodmann area_45
122 Brodmann area_46
123 Brodmann area_47
124 Caudate Tail
125 Caudate Body
126 Caudate Head
127 Dentate
128 Ventral Anterior Nucleus
129 Ventral Posterior Medial Nucleus
130 Ventral Posterior Lateral Nucleus
131 Medial Dorsal Nucleus
132 Lateral Dorsal Nucleus
133 Pulvinar
134 Lateral Posterior Nucleus
135 Ventral Lateral Nucleus
136 Midline Nucleus
137 Anterior Nucleus
138 Mammillary Body
139 Fourth Ventricle
140 Optic Tract
141 Anterior Commissure
142 Corpus Callosum
143 Third Ventricle
144 Medial Globus Pallidus
145 Lateral Globus Pallidus
146 Nucleus Accumbens Septi
147 Medial Geniculum Body
148 Lateral Geniculum Body
149 Subthalamic Nucleus
150 Lateral Ventricle
151 Putamen
152 No data

Appendix A

File Formats

This appendix describes the various file formats used by the Tina system to store intermediate results, calibration and other information on disc. For more generic storeage of arbitrary data structures the programmer is referred to the *serialisation* process described in the programmers guide.

A.1 AIFF Images

The following specifies the header of the Aivru Image File format.

```
         Field           Values            Meaning.
no       name

1      magic          unsigned short    byte reversal code.
2      header_length  short             length of header (bytes)
3      image_type     short             unsigned char etc.
4      height         short             image height (pixels)
5      width          short             image width  (pixels)
6      lx             short             low x of ROI
7      ly             short             low y of ROI
8      ux             short             upper x of ROI
9      uy             short             upper y of ROI
10     source_id      short             id. of camera (if any)
11     process_id     short             processing performed
12     date           char[8]           dd/mm/yy (if any)
13     time           char[8]           hh:mm:ss (if any)
14     title          char[80]          image title (if any)
```

This header has been derived according to requirements of over a decade of machine vision research. In contrast to standard file formats such as GIF it supports a wide variety of image types and arbitrary image co-ordinates, including image regions.

Notes:

- The header is usually 512 bytes long.
- Fields 1-9 must be present, but the other are at the descretion of the programmer.
- The following imae types are currently supported.

```
0  char
1  unsigned char
```

```
2  short
3 unsigned short
4 int
5 unsigned int
6 float
7 double
8 complex
9 ptr
10 vram0
11 vram1
12 vram2
13 vram3
```

The *ptr* filed is included only for completess and will not actually store the associated data. In order to write more complex Tina image data structures to disc the *serialisation* mechanism is recommended.

- The data for an image consistes of a sequence of bytes of the image data within the ROI in raster scan order.
- Date and time are not null terminated.
- The byte reversal magic field should always have the value 8516 (hex). This field may be used to check that the image is aiff format.
- The title field is null terminated text.

A.2 Edge Files

An initial 4 byte integer indicates the number of edges in the file. Edge data is stored in binary format as quadruples of floats (row, column, contrast and orientation) in raster order. Row and column are sub-pixel locations of the edge with the centre of a pixel defined at (x+0.5,y+0.5). The edge data structure can be used for generic representation of well located image features. Orientation and contrast measures are feature dependant.

A.3 Geometry File formats

Geometry files are stored as ASCI to facilitate user modification. Currently only points lines and conics are output. All data is in 3D, 2D features have their 3rd ordinate set to zero. This facilitates transparent exchange of 2D and 3D data structures from file (ie 2D data can be read as a planar geometrical object in the *Stereo Tool*).

Straight lines are represented in the following overdetermined fashion.

```
label        entry identifier
visibility   number of times the entry has been seen
length
end1         3 vector end point
end2         3 vector end point
direction    3 vector from end1 to end2
```

Ellipses are described as follows.

```
label        entry identifier
centre       3 vector ellipse centre
x-axis       3 vector conic axis.
```

```
y-axis        3 vector conic axis.
theta         major axis of elipse w.r.t ellipse coordinates.
alpha         length of major axis
beta          length of minor axis
t1            angular start point (radians)
t2            angular end point (radians)
```

A.4 Calibration Data

Calibration data for each camera is stored as ASCI to facilitate user modification. The data is stored as follows;

```
(image width) (image height)
(pixel size) (aspect ratio)
(centre_x) (centre_y)
3x3 rotation matrix
3 vector co-ordinate translation of camera origin
(number of distortion parameters) (distortion parameters)
```

Notes:

- one of the aspect ratios will generally be set to 1.0.
- the absolute coordinate frame of the camera is determined by the coordinate system of the data used during calibration.
- the origin of the camera is at the optical centre.
- the specification of distortion terms is optional and the distortion model used within Tina is programmer definable.

A.5 Matcher Files

A.6 OLD format

The matcher functions have associated context files which are used to store the associations between components of the scene and model as follows;

```
(initial data count)
FF n GF1 GF2 ..... GFn
```

where FF is the focus feature; n in the count of group features that form the context of that focus feature adn GFi are those features. All elements of the file are integers.

A.7 NEW format

The view based context files are listed as follows

```
(number of views)
V x y x N X
G M1 M2 ( a1 a2 a3 a4 ) [ b ] { c1 c2 d}
```

Where x, y, z is the view direction to the camera in the cooridnate system of the model.

N is the number of views.

X currently unused.

G specifies visibility of a geometry feature (E, or S) form the wireframe defined in the .poly file, with number M . Occluding lines (O) linking two ellipses are specified with both $M1$ and $M2$.

a_n are the allowed inward and outward lateral shifts (two for lines, four for ellipses) measured in millimetres.

b is the fisher information associated with localisation (currently unused).

c_n are the fraction of detectable locations expected to pass the detection hypothesis tests ($c > 0.5$ denotes 'mostly' visible).

A.8 Multi-Dimensional MR model

The current file format (for 4 images and 6 tissues) is as follows;

```
6 4   0.00000000000000                                % no. tissues, no. images, and P bknd
-11.18261 55.49973 20.53443 54.27743 214547738.6      % tissue 1 mean grey levels and %f output
-75.64249 656.3270 141.9607 320.3454 9782790526.2     % tissue 2
193.374 1603.260 773.691 1726.193 28136396881.3       % tissue 3
-1683.5 1410.6 1213.6 419.7 32192990488.70            % tissue 4
-679.3 1207.2 485.7 729.2 3181260321.3                % tissue 5
-158.4 927.4 265.3 545.7 818664305.7                  % tissue 6
0.014911 0.007032 -0.004771 -0.002422                 % tissue 1 (sgn sqrt) covariance matrix
0.007032 0.014436 -0.003948 -0.008038
-0.004771 -0.003948 0.033452 0.003107
-0.002422 -0.008038 0.003107 0.026677
0.009240 0.003762 -0.003949 -0.003866                 % tissue 2
0.003762 0.006712 -0.004440 0.001529
-0.003949 -0.004440 0.012580 -0.006741
-0.003866 0.001529 -0.006741 0.006901
0.007343 0.001840 0.002799 -0.003006                  % tissue 3
0.001840 0.006496 -0.003195 -0.000892
0.002799 -0.003195 0.007765 -0.004166
-0.003006 -0.000892 -0.004166 0.004639
0.003635 0.002003 0.004718 0.002054                   % tissue 4
0.002003 0.008971 -0.004602 0.003131
0.004718 -0.004602 0.009199 0.002793
0.002054 0.003131 0.002793 0.006415
0.009779 0.003218 0.005548 -0.003730                  % tissue 5
0.003218 0.012222 -0.005402 -0.006993
0.005548 -0.005402 0.011663 -0.005920
-0.003730 -0.006993 -0.005920 0.011071
0.012656 0.003622 -0.000290 0.004644                  % tissue 6
0.003622 0.015108 -0.001568 -0.006451
-0.000290 -0.001568 0.018347 -0.008170
0.004644 -0.006451 -0.008170 0.014645
5151.760 526.832 0.000 283.186 390.908 0.00           % matrix of density terms
526.832 2513.431 767.515 0.000 0.000 0.000            % 0 entries not used
0.000000 767.515687 202.769 0.000 0.000 0.000
283.186 0.000 0.000 66.907 1221.059 0.000
390.908 0.000 0.000 1221.059 4415.727 4726.13
0.000 0.000 0.000 0.000 4726.131 2484.088
air_bone skin_muscle fat CSF GM WM                    % tissue labels
```

```
0.319973 0.740320 1.000000 0.343430 0.432863 1.000000   % matrix of slope terms
0.740320 1.069539 0.440569 1.000000 1.000000 1.000000   % if present
1.000000 0.440569 1.585834 1.000000 1.000000 1.000000
0.343430 1.000000 1.000000 2.376265 0.408074 1.000000
0.432863 1.000000 1.000000 0.408074 0.734229 0.243779
1.000000 1.000000 1.000000 1.000000 0.243779 0.499773
```
