# Supplementary material for: TINA manual landmarking tool: software for the precise digitization of 3D landmarks
Source: Front Zool. 2012 Apr 5;9:6. doi: 10.1186/1742-9994-9-6 (PMC3353871; doi:10.1186/1742-9994-9-6)
Supplement: Additional file 7 — List of landmarks used for test of accuracy. Landmarks were assigned to the three landmark types 1, 2, and 3 (see text for details). The first three landmarks were used to define the median plane (PL1-3), landmarks 4 and 5 to define the horizontal axis (A1-2). [file 1742-9994-9-6-S7.DOC]

| **Landmark number** | **Landmark name** | **Landmark type** |
| --- | --- | --- |
| 1 | Anterior-most median tip of maxilla (PL1) | 2 |
| 2 | Intersection of frontals and parietals (PL2) | 1 |
| 3 | Anterior-most median point of foramen magnum (PL3) | 2 |
| 4 | Anterior-most point of molar row crowns (A1) | 2 |
| 5 | Posterior-most point of molar row crowns (A2) | 2 |
| 6 | Posterior-most median point of skull | 3 |
| 7 | Dorsal-most median point of skull | 3 |
| 8 | Posterior median end of suture between nasals | 1 |
| 9 | Posterior median end of suture between parietals | 1 |
| 10 | Posterior-most point of otic capsule | 3 |
| 11 | Ventral-most point of otic capsule | 3 |
| 12 | Lateral-most point of zygomatic arch | 3 |
