# Supplementary figures and images for: TINA manual landmarking tool: software for the precise digitization of 3D landmarks
Source: Front Zool. 2012 Apr 5;9:6. doi: 10.1186/1742-9994-9-6 (PMC3353871; doi:10.1186/1742-9994-9-6)

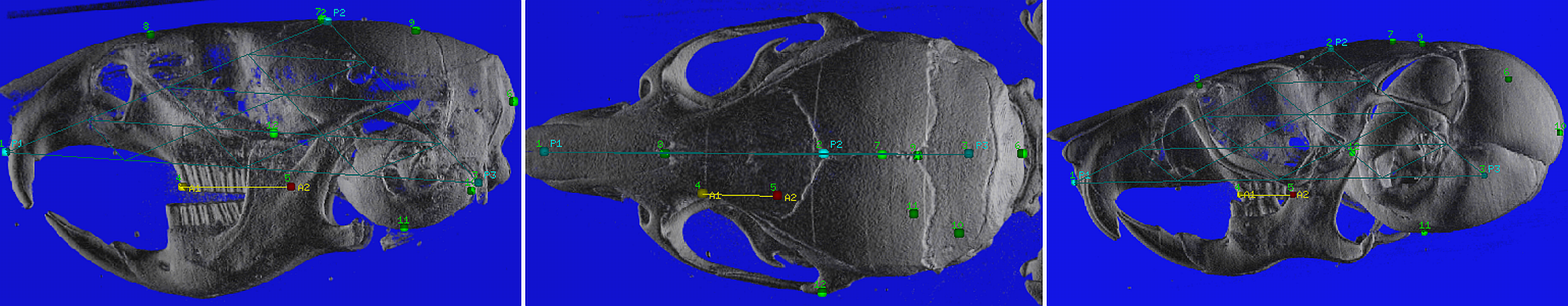

Supplement: Additional file 8 — Landmarks used for accuracy test. Skulls, landmarks, planes, and axes used in the test for accuracy of repeated measurements. Left: Microtus (lateral view), middle: Mus (dorsal view), right: Pachyuromys (lateral view). [file 1742-9994-9-6-S8.TIFF]

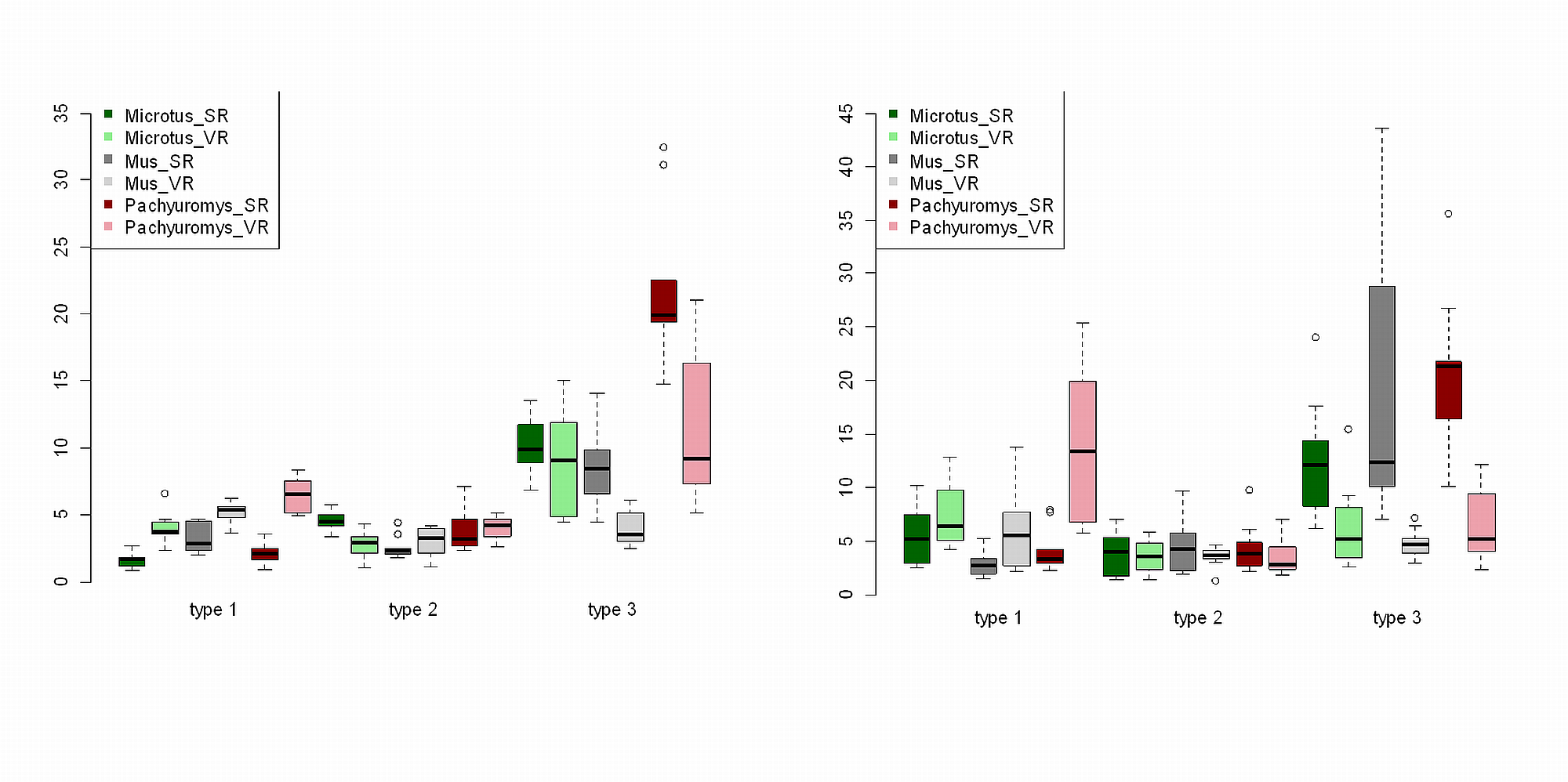

Supplement: Additional file 10 — Precision of landmarks with different methods. The distance in voxels from the median of the respective landmark position was taken and grouped for each landmark type (see text for details). Left: experienced user, right: inexperienced user; SR: surface rendering, VR: volume rendering. [file 1742-9994-9-6-S10.TIFF]
