# Supplementary material for: TINA manual landmarking tool: software for the precise digitization of 3D landmarks
Source: Front Zool. 2012 Apr 5;9:6. doi: 10.1186/1742-9994-9-6 (PMC3353871; doi:10.1186/1742-9994-9-6)
Supplement: Supplementary file 6 [file 1742-9994-9-6-S9.DOC]

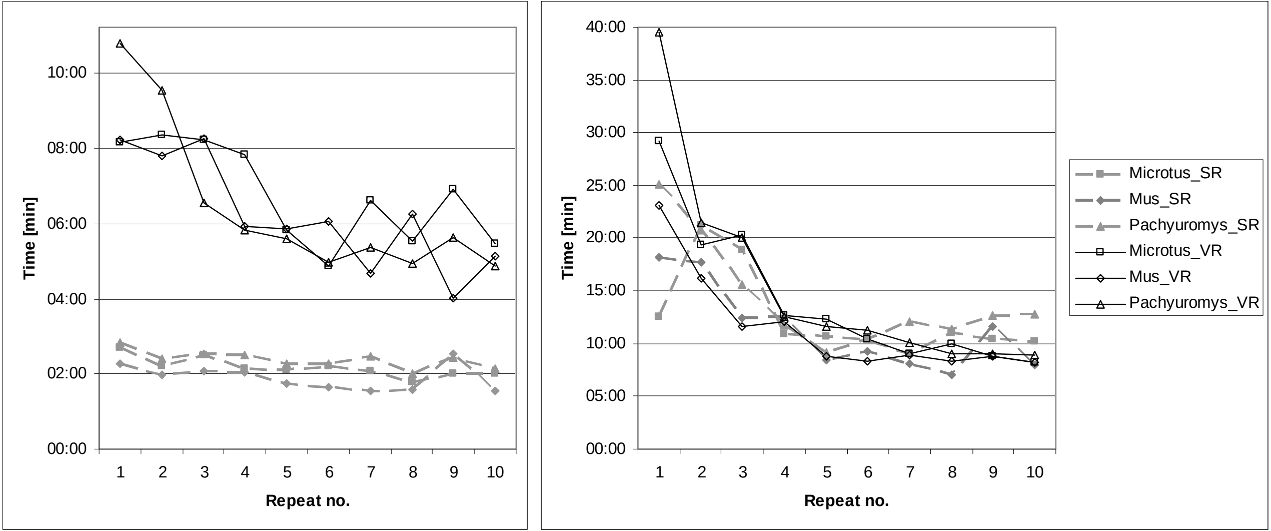

10:00
08:00
06:00
04:00
02:00
00:00
Time [min]
1 2 3 4 5 6 7 8 9 10
Repeat no.
40:00
35:00
30:00
25:00
20:00
15:00
10:00
05:00
00:00
Time [min]
1 2 3 4 5 6 7 8 9 10
Repeat no.
Microtus_SR
Mus_SR
Pachyuromys_SR
Microtus_VR
Mus_VR
Pachyuromys_VR
